# Mutations in *Rht-B1* Locus May Negatively Affect Frost Tolerance in Bread Wheat

**DOI:** 10.3390/ijms23147969

**Published:** 2022-07-19

**Authors:** Gabriella Szalai, Mihály Dernovics, Orsolya Kinga Gondor, Judit Tajti, Anna Borbála Molnár, Magdalena Anna Lejmel, Svetlana Misheva, Viktória Kovács, Magda Pál, Tibor Janda

**Affiliations:** 1 Department of Plant Physiology and Metabolomics, Agricultural Institute, Centre for Agricultural Research, ELKH, Brunszvik u. 2., H-2462 Martonvásár, Hungary; dernovics.mihaly@atk.hu (M.D.); gondor.kinga@atk.hu (O.K.G.); tajti.judit@atk.hu (J.T.); anna.b.molnar@gmail.com (A.B.M.); magdalena.lejmel@gmail.com (M.A.L.); kovacs.viktoria@atk.hu (V.K.); pal.magda@atk.hu (M.P.); 2 Department of Plant Ecophysiology, Institute of Plant Physiology and Genetics, Bulgarian Academy of Sciences, 1113 Sofia, Bulgaria; s_landjeva@mail.bg

**Keywords:** cold acclimation, flavones, GCxGC-TOFMS, LC-ESI-HR-MS, metabolomics, oligosaccharides, polyamines

## Abstract

The wheat semi-dwarfing genes *Rht* (*R*educed *h*eigh*t*) are widely distributed among the contemporary wheat varieties. These genes also exert pleiotropic effects on plant tolerance towards various abiotic stressors. In this work, frost tolerance was studied in three near-isogenic lines of the facultative variety ‘April Bearded’ (AB), carrying the wild type allele *Rht-B1a* (tall phenotype), and the mutant alleles *Rht-B1b* (semi-dwarf) and *Rht-B1c* (dwarf), and was further compared with the tolerance of a typical winter type variety, ‘Mv Beres’. The level of freezing tolerance was decreasing in the order ‘Mv Beres’ > AB *Rht-B1a* > AB *Rht-B1b* > AB *Rht-B1c*. To explain the observed differences, cold acclimation-related processes were studied: the expression of six cold-related genes, the phenylpropanoid pathway, carbohydrates, amino acids, polyamines and compounds in the tricarboxylic acid cycle. To achieve this, a comprehensive approach was applied, involving targeted analyses and untargeted metabolomics screening with the help of gas chromatography/liquid chromatography—mass spectrometry setups. Several cold-related processes exhibited similar changes in these genotypes; indeed, the accumulation of eight putrescine and agmatine derivatives, 17 flavones and numerous oligosaccharides (max. degree of polymerization 18) was associated with the level of freezing tolerance in the ‘April Bearded’ lines. In summary, the mutant *Rht* alleles may further decrease the generally low frost tolerance of the *Rht-B1a*, and, based on the metabolomics study, the mechanisms of frost tolerance may differ for a typical winter variety and a facultative variety. Present results point to the complex nature of frost resistance.

## 1. Introduction

The introduction of certain reduced height (*Rht*) genes into bread wheat played an important role in the ‘Green Revolution’, and their popularity with breeders is still unbroken. These alleles result in the semi-dwarf stature of plants with increased grain yield [1]. To date, 23 dwarfing genes have been described in wheat. Among them, *Rht-B1b*, earlier called *Rht1*, and *Rht-D1b* (earlier designated as *Rht2*) are located on the 4BS and 4DS chromosome arms, respectively, and are still extensively used in wheat breeding. At present, approximately 70% of wheat varieties worldwide contain at least one of these genes [2]. The Rht-B1c allele, earlier called Rht3, led to a dwarf phenotype, and its drought tolerance has been characterised earlier [3,4].

Global climate change, which is accompanied by progressively increasing temperatures and the occurrence of more frequent drought periods and temperature extremes, poses new challenges for breeders. Warming temperatures may also influence the type of freezing stress that winter cereals experience over winter. In many regions, it may easily happen that due to a relatively warm autumn period, plants cannot reach their potentially highest level of freezing tolerance, so a sudden freezing may be more harmful than if they would have been exposed to colder hardening temperatures. In spite of intensive research on the molecular characteristics of the *Rht* genes and their effects on yield [2,5,6,7], their effects on the stress tolerance in wheat plants are still less understood. 

Besides the primary effects of the wheat *Rht* genes on plant height, lodging resistance, and hence, grain yield, pleiotropic effects have been reported. So, earlier results showed that wheat plants carrying *Rht-B1c* and *Rht-B1b* alleles performed better under drought conditions than the tall lines carrying the wild-type allele *Rht-B1a* lines [3,4,7], having better membrane integrity and enhanced osmoregulation, and furthermore, exhibiting more efficient antioxidant defence. Dwarf *Rht-B1c* plants were also characterised by more stable photosynthesis with delayed non-stomatal limitation, and better water use efficiency under drought conditions than *Rht-B1a* plants [8]. Enhanced cadmium tolerance was also demonstrated in the *Rht-B1c* dwarf genotype [9]. This could be at least partly due to a metabolic shift from the synthesis of polyamine and proline to toxic metal chelator molecules, such as phytochelatins [10].

In wheat, the wild counterparts of the reduced height genes encode DELLA proteins (DELLAs). In plants, DELLA proteins (DELLAs) function as transcriptional repressors in the gibberellic acid (GA) signalling pathway, where GA promotes their degradation via the ubiquitin-proteasome pathway [11,12]. Mutant alleles at the *Rht* loci encode modified DELLA proteins, which are unable to interact with the GA receptor, GID1 (GA-INSENSITIVE DWARF1), resulting in reduced GA responsiveness even at adequate hormone levels, which may lead to poorer growth capacity [6,13]. The role of DELLAs in interactions between the GA and abscisic acid (ABA) signalling and metabolic pathways has also been described [11]. However, the exact mode of action of DELLAs in stress responses is still poorly understood.

Freezing tolerance is one of the most important physiological and agronomical characteristics of winter cereal varieties. This is a multigenic trait, affected by various stress-related mechanisms, including the acclimation of the photosynthetic machinery, the adjustment of redox homeostasis, antioxidant capacity and hormonal balance, inducing protective compounds, such as osmolites, polyamines, etc. [14,15,16,17]. Exposure to low, but non-freezing, temperatures induces several transcription factors, for example products of the C-REPEAT BINDING FACTOR (CBF) genes, which regulate the induction of the cold-regulated (COR) genes. Furthermore, exposure to low temperatures is required for the vernalisation processes in winter wheat. Earlier studies suggest that the main vernalisation gene, VRN-1, is required for the initiation of the regulatory cascade that down-regulates the cold acclimation pathway [18]. However, the effects of DELLA-encoding *Rht* alleles on the freezing tolerance of wheat have not yet been intensively studied. It was established in *Arabidopsis* that DELLAs collaborate with the C-repeat binding factors (CBFs) to restrain plant growth in response to low temperature stress [19,20]. The complexity of frost tolerance has been reviewed recently by Ding et al. [21].

Metabolomics approaches, especially untargeted ones, might offer a general tool to focus on pathways not previously considered when searching for genes related to frost/freezing tolerance. A recent GC-MS-based study was able to annotate 38 low molecular weight compounds that might contribute to or be related to frost tolerance [22]; however, LC-MS based untargeted metabolomics has rarely been applied in this field [23]. Indeed, despite the evident advantage of the high compound identification rate of GC-MS, LC-MS is able to detect metabolites with a far wider molecular weight range, without the need for inherent or derivatization-coupled volatility. Although LC-MS-based untargeted metabolomics are also subject to bottlenecks, including software- and database-related issues [24], they have been proven to serve wheat science, e.g. in the detection of wheat fraud [25], in the study of wheat leaves exposed to *Fusarium* [26] and in cases of low nitrogen stress [27]. 

Since semi-dwarf Rht-B1b and dwarf *Rht-B1c* plants have been found to have better drought tolerance than the tall wild-type genotypes, and since a relationship has been demonstrated between osmotic stress tolerance and freezing tolerance, we hypothesised that certain Rht genes may also have an influence on the development of freezing tolerance in wheat plants. Therefore, the present work aimed to determine the level of freezing tolerance of wheat lines carrying the alleles *Rht-B1b* (semi-dwarfing) and *Rht-B1c* (dwarfing) compared with that of the tall, wild-type *Rht-B1a* allele in the background of a facultative wheat variety, and further to compare this with the response of a typical winter variety. Certain cold acclimation-related processes were also examined, focusing mainly on metabolomic aspects, in order to find possible explanations for the different levels of freezing tolerance.

## 2. Results

### 2.1. Rht-B1b and Rht-B1c Alleles Reduced the Freezing Tolerance of Wheat

**Figure 1:**
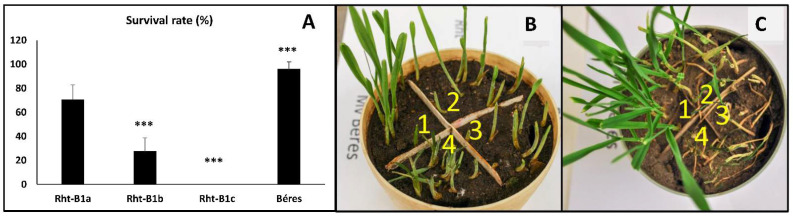
Survival rate at −10 °C after 3 days of recovery period at 20 °C (**A**). Representative photos demonstrating the survival of wheat genotypes (1: Mv Béres; 2: Rht-B1a; 3: Rht-B1b; 4: Rht-B1c) after 1-day freezing at −10 °C (**B**) and after −11 °C (**C**). Photos were taken after 3 (**B**) or 10 days (**C**) of the recovery period at 20 °C. ***: significant at *p* < 0.001 level.

In the first set of experiments, the effects of the Rht-B1b and Rht-B1c alleles were investigated compared with the control Rht-B1a plants. Since this genotype is of the facultative growth type and is characterised by relatively low frost tolerance, another reference variety, the winter type Mv Béres was also used in this part of the experiments. After growing at 22/20 °C for 2 weeks, the plants were cold hardened at 5 °C for 12 days. In order to differentiate between the four genotypes, all of them were sown in each pot, and they were exposed to −10 °C or −11 °C freezing temperatures in a frost chamber. Due to inhomogeneity within the freezing box, only comparison within the same pot was possible. The whole freezing experiment was repeated five times, and similar trends were observed all the time. Figure 1 shows the survival percentage at −10 °C (Figure 1A) and representative photos of the plants during the regrowth period which followed the 1-day freezing. Both subfigures (Figure 1B,C) clearly show that Mv Béres had the highest level of freezing tolerance, exhibiting fast, stable recovery. In contrast, Rht-B1c was the weakest and was unable to recover. The wild-type Rht line (Rht-B1a) showed a slow but stable recovery. Figure 1B shows that the semi-dwarf Rht-B1b after freezing at −10 °C exhibited initial regrowth, which was manifested in the survival percentage (Figure 1A). However, after a few days, most of these plants stopped growing, and they also died. When the freezing test was carried out at −11 °C, all the plants of this genotype died, without initial growth (Figure 1C). These visual observations confirmed that the reference genotype Mv Béres had a higher freezing tolerance than the Rht-B1a line. Furthermore, the presence of the Rht-B1b and especially the Rht-B1c allele led to a reduction in the freezing survival rate. So, the freezing tolerance of the investigated genotypes decreased in this order: Mv Béres > Rht-B1a > Rht-B1b > Rht-B1c.

### 2.2. Polyamines, Ascorbate Peroxidase and Flavonols in ‘April Bearded’ near Isogenic Lines (NILs)

In order to obtain a better understanding of the effects of Rht alleles on the freezing tolerance of wheat plants, in the next set of experiments the levels of certain stress-related compounds, proved to be related to frost resistance, were analysed in wild-type Rht-B1a and mutant Rht-B1b and Rht-B1c dwarf lines. Although the most important part for survival is the crown, the processes in the leaf during hardening are responsible for the synthesis of protective compounds. Among the free polyamines, SPD (25–30 µg g^−1^ fresh weight) was the most abundant compound, followed by SPM (30 µg g^−1^ fresh weight) and PUT (7–11 µg g^−1^ fresh weight) under control conditions (Figure S1). The degradation product DAP and CAD, a polyamine synthesized in a different pathway from that of PUT-SPD-SPM, could only be detected at much lower levels (3 and 1 µg g^−1^ fresh weight, respectively). In general, exposure to cold hardening at 5 °C for 12 days significantly increased the content of PUT (five-eightfold), SPD (threefold) and CAD (four-sevenfold), and decreased that of DAP and SPM to half in the leaves. However, no substantial differences between the genotypes could be detected.

Since the ascorbate peroxidase (APX) activity in the leaves usually increases during the cold hardening period, this was also measured in the three ‘April Bearded’ NILs (Figure S2). Growth at 5 °C for 12 days did indeed increase the activity, but like the native polyamine levels, no significant differences were found between the lines.

The flavonol contents, including kaempferol, quercetin, myricetin and rutin, did not differ considerably between the ‘April Bearded’ NILs, either in the control or in the cold-hardened plants (Figure S3). Growth at 5 °C substantially increased the quercetin levels in all the studied ‘April Bearded’ genotypes.

### 2.3. Expression of Certain Stress-Related Genes

**Figure 2:**
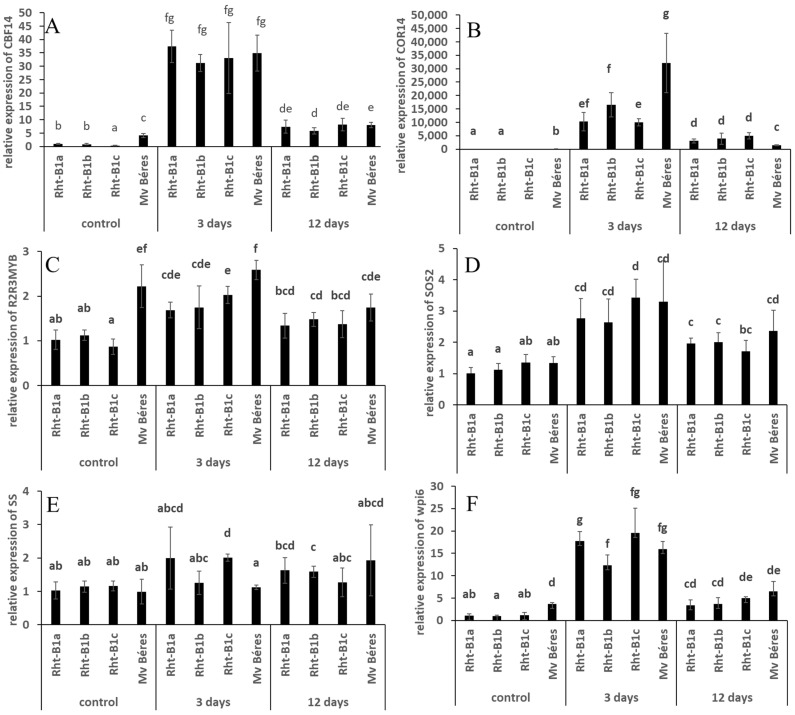
Effects of 3 or 12 days of cold hardening at 5 °C on the CBF14 (**A**), COR14b (**B**), R2R3MYB (**C**), SOS2 (**D**), SS (**E**) and wpi6 (**F**) genes in the leaves of different young studied ‘April Bearded’ genotypes and the Mv Béres variety. Control values represent data for unhardened plants. The relative gene expression was determined with the 2-ddCt method. Different letters indicate significant differences at the *p* ≤ 0.05 level.

Since the compounds expected to be stress-related did not exhibit genotype dependence during the cold hardening period in the ‘April Bearded’ NILs, the expression levels of certain stress-related genes were also determined in control and cold-hardened leaves, and at the beginning of the freezing period (Figure 2). CBF14 is a cold-inducible gene encoding a CBF (C-repeat/DRE binding factor) transcription factor, which plays a role in the regulation of certain cold-regulated genes [28]. Under control conditions, the expression level of the CBF14 gene was slightly but significantly lower in Rht-B1c and higher in Mv Béres than in the other genotypes. However, the expression level substantially increased after 3 days of cold treatment at 5 °C, and the differences between the genotypes disappeared. As the cold treatment continued, the high expression level subsided, and was only slightly higher after 12 days than in the control plants. COR14b is also a cold-inducible gene, which may be directly related to freezing tolerance in various plant species [18]. Its expression level is very low under control conditions: no PCR product could be detected by RT-qPCR in Rht-B1c. The expression levels substantially increased after 3-day cold treatment, especially in Mv Béres. However, after longer exposure to cold, the expression levels dramatically decreased in all the genotypes. R2R3MYB encodes a MYB transcription factor. MYBs are involved in various regulatory processes, including responses to environmental stress factors and secondary metabolic pathways [29]. Under control conditions, the expression level in Mv Béres was significantly higher than in the studied ‘April Bearded’ genotypes. Three days of cold hardening mainly induced gene expression in the ‘April Bearded’ plants. At the second sampling time during cold hardening, the expression of the gene again showed a tendency to decline. TaSS encodes a sucrose synthase gene with a role in carbohydrate accumulation [30]. There were no differences in the expression levels of the TaSS gene in control samples, and although its expression increased slightly in Rht-B1c, longer cold treatment did not generate substantial changes. The Tawpi6 gene encodes a cold-inducible protein located in the plasma membrane. In control plants, the expression of Tawpi6 was higher in Mv Béres than in the studied ‘April Bearded’ genotypes. However, after exposure to cold, which induces its expression, this difference disappeared, leading to similar values for all the genotypes tested. Similarly, to the other genes, its expression level was higher after short-term cold treatment (3 days) than after longer cold exposure. The SOS2 (Salt Overly Sensitive 2) gene encodes a protein kinase involved in maintaining ionic homeostasis, especially at high salinity [31]. Interestingly, the expression level of this gene also increased after short-term cold exposure. However, as for many of the other cold-responsive genes investigated, their expression levels did not differ significantly between genotypes.

### 2.4. Untargeted Metabolomics Analyses Using GCxGC-TOFMS

Since none of the previously investigated processes related to frost tolerance could unambiguously explain the differences between the studied ‘April Bearded’ genotypes, the plants were subjected to detailed metabolomic analyses. In the first step, samples were collected from control, unhardened plants and after 2, 7 and 12 days of cold treatment. Using the GCxGC-TOF MS set-up, various groups of methanol/water-soluble small molecules were analysed, and amino acids, sugars, organic acids, and certain related compounds were detected (Table S3). Among the 13 amino acids detected, the most significant changes were found in the levels of glycine and proline. The amount of glycine was twice as high in Mv Béres than in the ‘April Bearded’ NILs under control conditions, but during cold acclimation the ‘April Bearded’ NILs accumulated much more glycine than Mv Béres did, the highest amount being detected in Rht-B1c after 12 d at 5 °C. Dimethyl-glycine, the precursor of glycine-betaine, tended to increase during the cold acclimation period in all the genotypes, and in Mv Béres it was slightly higher than in the ‘April Bearded’ NILs, where its values did not differ. There was no difference in the proline level in unhardened control plants, but its level dramatically increased, especially in the Rht-B1a plants, after 2 days at 5 °C. The lowest level was detected in Rht-B1c plants. The same tendencies were found throughout the 12 days of acclimation. Sixteen carbohydrates were also identified (Table S1). The amount of fructose dropped dramatically after 7 days of acclimation, while elevated levels of glucose, mannose, cellobiose and maltose were detected after 7 days, all of which decreased to the initial level later; however, there was no significant difference between the genotypes. Four unidentified trisaccharides (T1, T2, T3 and T4) were also found during the acclimation period. In the control plants, their levels were very low or below the detection limit, while during the 12 days of acclimation, a huge accumulation could be detected. In the case of T1 and T4, there were no significant differences between the genotypes. The amount of T2 was below the detection limit again after 12 days in Rht-B1c plants, when the highest level was detected in Mv Béres. In the case of T3, the highest level was found in Mv Béres after 7 days, but it had dropped below the detection limit in Rht-B1c and Mv Béres by the 12th day. Among the compounds involved in the citrate cycle, the amounts of iso-citrate, 2-keto-glutarate and oxaloacetic acid were below the detection limit. An increase in aconitic acid could be observed after 7 days and in fumaric acid after 12 days. This was more pronounced in the ‘April Bearded’ NILs than in Mv Béres in the case of fumaric acid. The amounts of succinic acid decreased during the first 7 days of acclimation, but returned to the initial level by the 12th day in the ‘April Bearded’ NILs. The level of malic acid decreased during cold acclimation, but there was no difference between the genotypes. A temporary increase was also found in the methylsuccinate level after 2 days in all the genotypes tested. The level of ferulic acid was below the detection limit in the control plants, but during cold acclimation a low but constant level could be detected in all the genotypes (Table S3).

### 2.5. Untargeted Metabolomics Analyses Using LC-TOF/MS

Untargeted LC-MS analyses on the metabolic responses of the ‘April Bearded’ NILs and the reference Mv Béres genotype when exposed to low temperatures were performed on methanolic extracts prepared from leaves collected 0, 2, 7 and 12 days after the beginning of cold hardening. As can be seen in Table S2, the number of differentiating metabolites ranged from 200 to 600, 8–65 of which possessed an intensity level high enough to be considered for MS/MS analysis. The latter is required for the assignment of molecules, as database-derived identification sets often contain an impossibly large number of potential hits. Both in positive and negative modes, the highest number of components differentiating between the ‘April Bearded’ NILs almost always appeared on the second day, indicating that differences develop after 2 days.

Among the compounds analysed with MS/MS, it proved possible to identify 25 molecules, eight polyamine derivatives and 17 flavonoids (Table 1). These 25 compounds and their in-source adducts/fragments usually made up a considerable part, sometimes 100%, of the entities selected for MS/MS analysis on the basis of their adequate intensities (Table S2). This suggests that these compounds represent a highly abundant set of molecules that exhibit a response when the wheat genotypes in question are exposed to cold stress.

Principal component analysis (PCA) was also carried out on the datasets arising from negative and positive mode experiments, taking into account either all the entities (2461 in negative and 11,064 in positive ion modes), only the 17 flavonoids (i.e. flavones), only the eight polyamine derivatives, or all the flavonoids and polyamines. This kind of sorting was supported by the observation that the two ionisation modes not only provided highly different numbers of detected molecules but also subgroups of molecules (i.e. polyamines) could either be detected in only one of the modes or in both modes, greatly influencing the outcome of PCA analyses.

**Figure 3:**
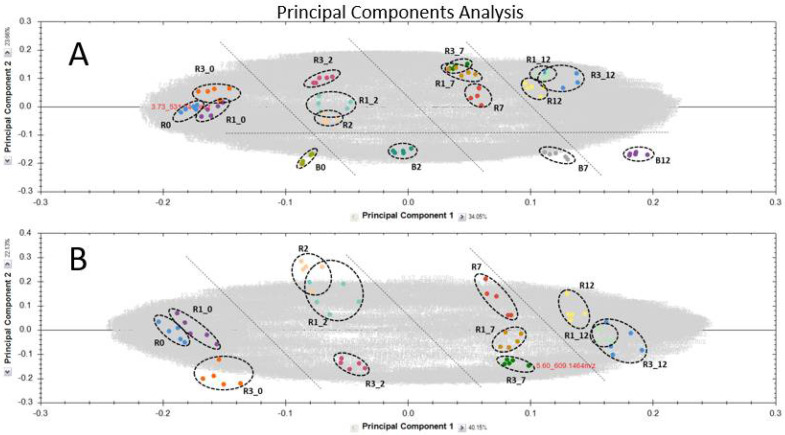
Principal component analysis (PCA) for all the molecules including (**A**; >11,000 compounds) or without (**B**; >2400 molecules) the Mv Béres genotype in ESI-mode. (R: Rht-B1a R1: Rht-B1b R3: Rht-B1c; B: Mv Béres; 0, 2, 7 and 12 days of hardening at 5 °C).

In addition, in order to evaluate effects specific to the Rht group, PCA analyses were done with or without the inclusion of the Mv Béres genotype. Figure 3A,B represent the PCA plots for all the detected compounds and for the sample groups. In negative mode: both the genotypes and the treatment days were clearly differentiated, and the exclusion of Mv Béres data further enhanced this separation. However, when the data for the PCA plots were used from positive mode acquisitions, it was not possible to distinguish between the three studied ‘April Bearded’ genotypes in terms of treatment days, even when the Mv Béres data were excluded (data not presented).

**Figure 4:**
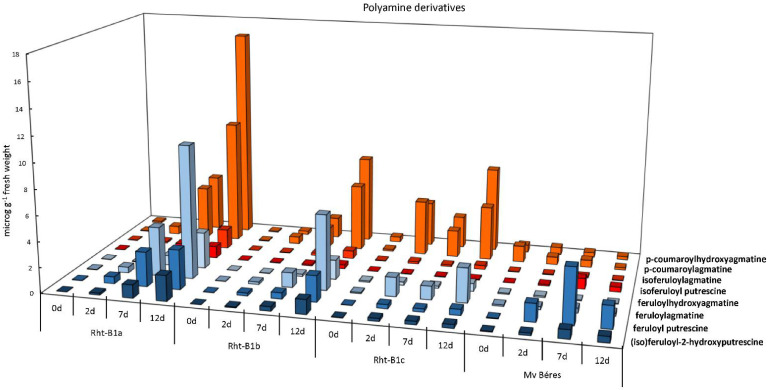
Polyamine derivatives in control (0 d) and cold-hardened young ‘April Bearded’ NILs and Mv Béres wheat genotypes (after the 2, 7 or 12 days of hardening at 5 °C).

The identification of the differentially accumulated metabolites indicated that several polyamine derivatives, namely (iso)feruloyl-2-hydroxyputrescine, feruloylputrescine, feruloylagmatine, feruloylhydroxyagmatine, isoferuloylputrescine, isoferuloylagmatine, p-coumaroylagmatine and p-coumaroylhydroxyagmatine, exhibited a substantial level of accumulation during exposure to low temperatures, and that this level also differed for individual wheat genotypes (Figure 4). Although the levels of certain phenylamides, such as p-coumaroylhydroxyagmatine, feruloylhydroxyagmatine, p-coumaroylagmatine and feruloylagmatine, were more than two-fold higher in the dwarf Rht-B1c line than in the wild-type Rht-B1a after short-term cold treatment (2 days), the cold-induced accumulation was most pronounced in the wild-type and least significant in the dwarf Rht-B1c after longer exposure. The semi-dwarf Rht-B1b line showed a medium level of accumulation, compared with Rht-B1a and Rht-B1c. Interestingly, Mv Béres only accumulated these compounds to a limited extent, and in some cases, such as p-coumaroylagmatine and p-coumaroylhydroxyagmatine, a continuous decrease was detected during the cold hardening period.

**Figure 5:**
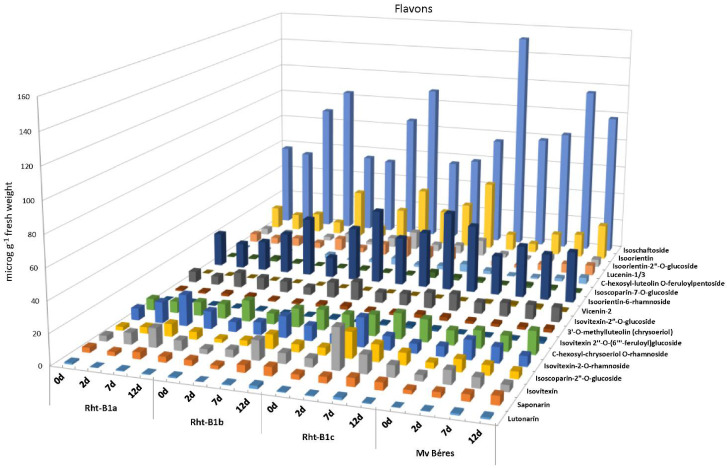
Estimated flavone contents in control (0 d) and cold-hardened young ‘April Bearded’ NILs and Mv Béres wheat genotypes (after the 2, 7 or 12 days of hardening at 5 °C).

Another group of compounds that showed significant differences between the genotypes was the flavones; 17 flavone compounds exhibited a significant low-temperature-induced increase in most of the genotypes tested. This increase was either continuous, for example in the cases of isoschaftoside in the ‘April Bearded’ lines or isoorientine in Mv Béres, or temporary, peaking on the second (for example, isovitexin in Rht-B1c) or seventh day (for example, isoschaftoside in Mv Béres) of the cold hardening period (Figure 5).

Figures S4–S6 represent the PCA plots for the 25 differentiating compounds only. It is clear that using a limited set of entries for the PCA calculation did not greatly increase the quality of the differentiation compared to Figure 3. As can be seen on Figure S4A, when all four genotypes were included, differences between the quantities of the eightpolyamine derivatives detectable only in positive ion mode were only visible on day 7 and day 12, which is in accordance with the greater abundance and complexity of the polyamine derivatives on these days. However, only when the datasets for the studied ‘April Bearded’ genotypes were considered, it proved possible to separate these three genotypes on days 0 and 2 as well (Figure S4B).

By contrast, when PCA was performed on the 17 flavones, using data from either positive or negative mode analysis (Figure S5), the clearest differentiation was observed between Mv Béres and the three studied ‘April Bearded’ genotypes, while less separation was visible between days (see Figure S5A for PCA results derived from ESI negative mode). When the Mv Béres genotype was excluded (Figure S5B), the Rht-B1a and Rht-B1b genotypes could be differentiated on days 2, 7 and 12, while Rht-B1c could be separated on all the sampling days when negative mode data were used; the sampling days could also be distinguished. When positive mode data were applied (Figure S5C,D), separation was much poorer for the Rht-B1a and Rht-B1b genotypes and from the point of view of the day of sampling.

When joint PCA analysis was performed on the eight-polyamine derivatives and 17 flavones, using positive mode data (Figure S6), certain separations could be observed between days and genotypes, e.g., the studied ‘April Bearded’ genotypes were clearly discriminated on day 12 when Mv Béres was excluded.

**Figure 6:**
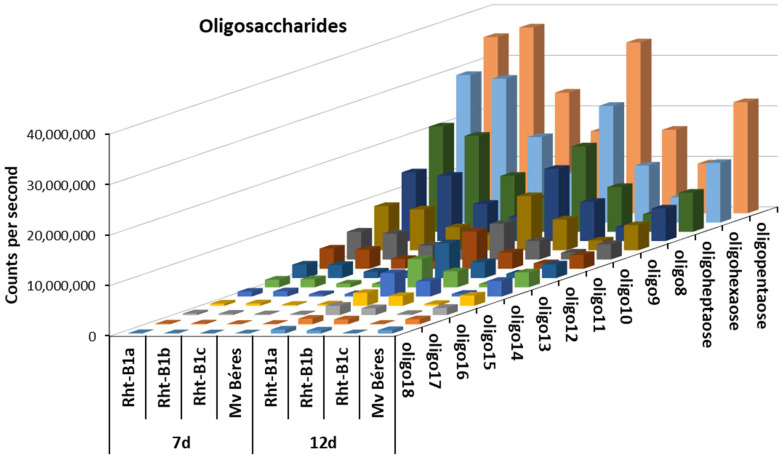
Oligosaccharide contents in young ‘April Bearded’ NILs and Mv Béres wheat genotypes cold-hardened for 7 or 12 days. In control and 2-day hardened plants all the values were below the detection limits.

When the other differentiating entities were subjected to further MS/MS analysis, homologous series of oligohexoses were only detected in sample groups collected 7 and 12 days after the beginning of cold hardening. These were eluted close to the void volume of the chromatographic system. In order to ensure adequate retention and separation, HILIC-ESI-QTOF-MS analysis was chosen. This set-up was previously proven to separate fructans with a degree of polymerization (DP) of up to 47 from a chicory extract [32]. To the best of our knowledge, the present study is the first to extend this technique to wheat fructans. Although fructans with 3–20 DP were detected, reproducible results were only obtained for DP = 5–18; over 18, the intensities were too low for relative quantification, while the accurate mass and adduct-based peak picking results for DP = 3 and 4 were too complex because of in-source fragment formation. When co-eluting, the individual species of such series cannot be distinguished as they produce in-source fragments that exactly match the accurate mass of the lower molecular weight members of the series. As presented in Table S3, oligohexoses containing 5–18 hexose moieties could be unambiguously detected and assigned as statistically differentiating. In general, the highest oligohexose levels were detected in the wild-type Rht-B1a and semi-dwarf Rht-B1b genotypes (Figure 6). In Rht-B1c, this level was usually higher than in Mv Béres after 7 days, but after 12 days the tendency was reversed, with an increase in Mv Béres and a decrease in Rht-B1c.

## 3. Discussion

Previous studies showed that the presence of the *Rht-B1c* or *Rht-B1b* alleles led to better drought tolerance than that found in the tall, wild-type *Rht-B1a* genotype [4]. However, the present results showed that the dwarf genotypes *Rht-B1b* and, especially, *Rht-B1c* had a lower level of freezing tolerance than *Rht-B1a*, and all of them performed worse than the reference winter variety Mv Béres. This can be explained by the fact that the tall variety ‘April Bearded’, carrying the wild-type allele *Rht-B1a*, has a facultative growth habit and low frost tolerance, as do the two *Rht* near-isogenic lines. The present experiment mimicked growth, hardening and freezing conditions, where due to global warming, plants are not fully hardened, but they are exposed to a sudden freezing stress. This may differ from other conditions, where plants can reach their maximum freezing tolerance. 

A further aim of the present work was to discover molecular mechanisms that could explain differences between the freezing tolerance of the studied ‘April Bearded’ genotypes and to elucidate the effects of the two mutant alleles (*Rht-B1b and Rht-B1c*) at the *Rht-B1* locus. 

Several studies have shown that free polyamines may play an important role in acclimation processes to abiotic stressors [17], including the development of freezing tolerance in plants [33]. The present data also showed that cold hardening increased the PUT, SPD and CAD levels in the ‘April Bearded’ NILs, with a parallel decrease in the SPM contents. Similar tendencies have been shown in cold acclimated *Thellungiella* and *Arabidopsis* *thaliana* genotypes [34]. The decrease in the DAP and SPM levels in cold-hardened plants also suggests the possible involvement of the polyamine cycle and its related signalling processes in the cold acclimation of these plants [35]. However, significant differences between the free polyamines, which could explain the different levels of freezing tolerance in ‘April Bearded’ NILs, were not detected.

The adjustment of the antioxidant system may also be part of the adaptation to environmental stresses. Growth at low temperatures may increase the concentration of reactive oxygen species, inducing oxidative stress [36,37]. A correlation has been demonstrated between the APX activity in cold-hardened leaves and the frost tolerance of cereal species [35,38]. Although growth at 5 °C did indeed increase the ascorbate peroxidase activity in the ‘April Bearded’ NILs, there was no difference between the genotypes. In the same way, changes in the levels of certain flavonols, which are components in the antioxidant system, were also similar in the ‘April Bearded’ NILs investigated. 

Frost tolerance is a multigenic, complex trait, and its mechanisms may also have a crosstalk with growth regulation. For example, a recent study indicated that low temperature also induced the SQUAMOSA PROMOTER BINDING PROTEIN-LIKE 9 (SPL9) gene in *Arabidopsis* plants. SPL9 may directly bind to the promoter of one of the CBF genes, leading to increased freezing tolerance [39]. Results in *Betula luminifera* indicated that certain SPLs may also interact with DELLA proteins, influencing the processes regulated by GA [40]. In order to obtain a better understanding of the molecular background of differences in the freezing tolerance of studied ‘April Bearded’ genotypes, stress-related, mainly cold-inducible genes were selected for investigation. However, although the selected genes, including the *SOS2* gene mainly involved in salt tolerance, were generally only cold-inducible for a limited time, they were not responsible for differences between the studied ‘April Bearded’ genotypes. For better understanding the differences between these lines, an RNA-seq study can be useful in future works.

**Figure 7:**
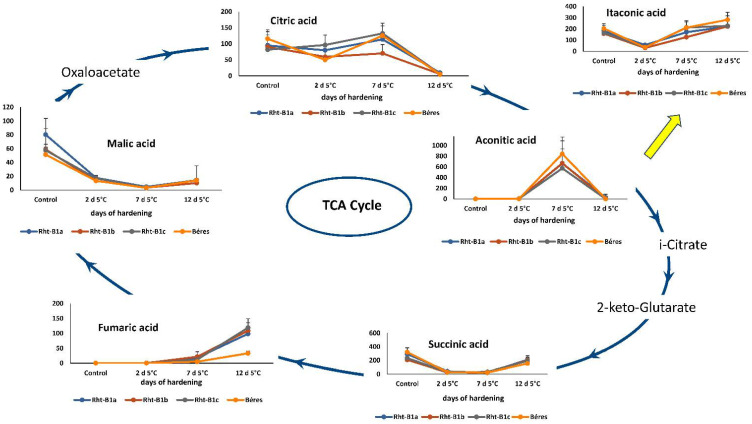
Changes in the components of the Tricarboxylic Acid (TCA) Cycle and certain related compounds during the 12-days of cold hardening period in ‘April Bearded’ NILs and the wheat variety Mv Béres.

Since the most widely used stress markers related to cold tolerance in wheat did not explain the differences in freezing tolerance, especially between the ‘April Bearded’ NILs, untargeted metabolomics analyses using the GCxGC/TOFMS and LC-ESI-QTOF/MS techniques were also applied to reveal metabolic alterations linked to cold responses. Exposure to low hardening temperatures also affected the elements of the TCA cycle and certain related compounds (Figure 7). Although the majority of the changes observed in the first 12 days were temporary, the amounts of fumaric acid and malic acid showed a progressive increase and decrease, respectively, suggesting that low temperatures may reduce the rate of fumarate-malate conversion, especially in the ‘April Bearded’ NILs, where the fumarate levels were significantly higher than in the Mv Béres variety, which had the best frost tolerance. The amount of malate could also be directly regulated by fumarate via the NAD-malic and/or NADP-malic enzymes, leading to a facilitated malate-pyruvate conversion at accumulated fumarate levels [41]. The other TCA compounds were usually present in similar quantities in all the genotypes tested.

**Figure 8:**
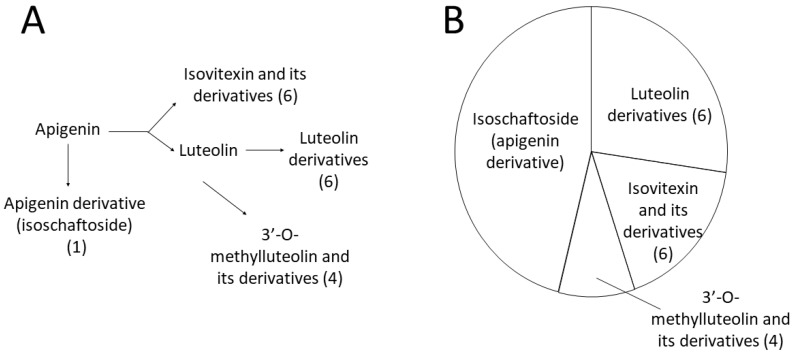
Biosynthetic relationships of the 17 differentiating flavones (**A**) and their contribution to the total flavone response (**B**), based on their summed abundance. (1), (4) and (6) are the numbers of the flavones and their derivatives. Names and detailed information can be found in Table 1.

The role of flavonoids in various stress-related processes has been widely studied and reviewed. A recent study also showed that they may contribute to the cold acclimation processes in *Arabidopsis* plants [42]. However, earlier research focused mainly on flavonols or anthocyanins, while flavones received less attention. Among other things, flavones are able to protect plants from UV-B radiation and may also act as natural pesticides [43]. As antioxidants, they may also provide protection against high salinity [44]. No authentic standards are available for most of the differentiating flavones, and their identification/assignment could only be based on LC-ESI-MS/MS fragmentation patterns, accurate mass data and relative retention time parameters, so quantitative analyses could not be carried out. Nevertheless, the abundance of data presented in Figure 5 and Figure 8 is useful for the indication of which pathways are involved in flavone biosynthesis. Isoschaftoside represented the most characteristic contribution to cold stress, while the other 16 flavones consisted of luteolin derivatives, isovitexin and its derivatives and 3′-O-methylluteolin and its derivatives. Neither apigenin nor luteolin was found among the differentiating flavones, indicating that these aglycones were not accumulated in response to cold stress; instead, their conjugation with sugar moieties or their conversion (e.g. methylation) could be linked to the special stress responses of individual wheat genotypes.

Among the compounds that were present in significantly higher abundance due to the cold stress, 17 could be assigned as flavonoids (Table 1), all of which were derivatives of the flavone apigenin, representing four subgroups, namely, isovitexin and its derivatives, luteolin derivatives, chrysoeriol and its derivatives and isoschaftoside. All in all, the complexity of these 17 flavonoids was the greatest in the *Rht-B1c* genotype (with very few exceptions in the case of Mv Béres). Regarding the individual flavonoid compounds, isoschaftoside was the most abundant species in all the genotypes, followed by isoorientin and isoorientin-6-rhamnoside. Interestingly, although the two feruloylated flavonoids, namely C-hexosyl-luteolin-O-feruloylpentoside and isovitexin-2″-O-(6‴-feruloyl)glucoside, were among the least abundant of the 17 differentiating flavonoids, they were present in significantly higher concentrations on days 2 and 7 in *Rht-B1c* than in any of the other genotypes (*Rht-B1a*, *Rht-B1b* and Mv Béres). It is not yet clear whether the increase in feruloylated polyamines, especially feruloylagmatine, on day 2 in the *Rht-B1c* genotype was connected to the increased ferulyolation of these two flavonoids. Generally speaking, the substantial, but sometimes temporary, increase in flavone content induced by cold hardening in wheat leaves was not correlated with the level of freezing tolerance in the investigated genotypes, suggesting that flavones probably play a role mainly in the acclimation to low, but non-freezing, temperatures and are less important for freezing tolerance. However, this does not exclude the possible contribution of flavonoids to the development of freezing tolerance, because recent results on various knock-out *Arabidopsis* mutant plants also showed that the lack of certain flavonoids could be compensated by other compound classes [42].

In contrast to free polyamines, several polyamine derivatives belonging to the hydroxycinnamic acid amides differentially accumulated at hardening temperature in the different wheat genotypes. The accumulation of hydroxycinnamic acid conjugates of polyamines was reported earlier, mainly in the case of biotic stresses, and there are only a few reports about their accumulation during abiotic stresses [45]. The *Rht-B1a* and *Rht-B1b* genotypes exhibited a clear trend of polyamine derivative accumulation, reaching both relative and absolute maximum values on day 12 and indicating the superiority of the *Rht-B1a* genotype over *Rht-B1b*, *Rht-B1c* and the reference Mv Béres genotype after 12 days of cold acclimation. Genotype *Rht-B1c* featured sudden peaks for *p*-coumaroylagmatine, *p*-coumaroylhydroxyagmatine, feruloylagmatine and feruloylhydroxyagmatine on day 2, before reaching their maximum values on day 12. In the studied ‘April Bearded’ genotypes, PUT derivatives only showed a significant increase from day 7, while Mv Béres had significantly higher levels of some PUT conjugates either inherently (day 0) or on day 2 compared to the studied ‘April Bearded’ genotypes. Although a variety of phenylamides have been detected in various plant species and their involvement in a number of physiological processes has been detected, they have only recently become the focus of research [46,47]. These compounds are generally known as important participants in plant-microbe interactions, especially in defence responses against pathogens [48], but feruloylputrescine may also participate in interactions with plant growth-promoting rhizobacteria [47]. Results also suggest that they may be involved in abiotic stress responses, including drought [49] or nutrient deficiency [50]. In a study on the cold acclimation of two wheat varieties [23], only three of the 10 polyamine derivatives detected (namely, p-coumaroylagmatine, feruloylputrescine and isoferuloylagmatine) and only 5–6 of the 20 flavonoids detected (namely, isoorientin, isovitexin, dihexosyl luteolin, *O*,*C*-rhamnosyl-glucosyl-luteolin, 6-*C*-glucosyl-*O*-glucosyl-chrysoeriol and chrysoeriol) were the same as those found in the present work (though some matches were uncertain due to differences in the synonyms used). This indicates a considerable difference in complexity between the wheat varieties involved in the studies. For example, while vitexin and orientin were among the four most abundant flavonoids, showing a significant increase after cold exposure in the study of Moheb et al. [23], the four genotypes studied here contained only traces of these two flavonoids and their derivatives. Isoorientin, isoschaftoside and isovitexin proved to be important biomarkers for other, stress-related wheat metabolome changes, e.g., for low nitrogen stress [27]. In agreement with the present findings, earlier work also showed that feruloylagmatine, characterised as being an antifungal and antioxidant compound, also accumulated at low temperatures in winter wheat plants [51]. The present results suggest that the accumulation of these phenylamides could be partially responsible for differences in the freezing tolerance of the ‘April Bearded’ NILs. However, they are probably not a critical factor in the frost tolerance of wheat plants for the following reasons: a cold-induced increase could be observed for most of the compounds detected; they are induced by various stress factors; and the cold-induced accumulation was less pronounced in the most tolerant variety, Mv Béres, than in the ‘April Bearded’ NILs. They probably contribute to the antioxidant defence system, because ozone also induces their synthesis [52].

In contrast to the flavonoids and polyamine derivatives, the differentiating oligohexoses (DP = 5–18) could only be detected after 7 or more days of cold hardening in all the genotypes. From the 7th day, *Rht-B1a* and *Rht-B1b* accumulated significantly more fructans than Mv Béres and *Rht-B1c*; in this comparison, *Rht-B1c* showed the lowest concentrations of differentiating metabolites, unlike the case of flavonoid derivatives. Their relative levels (*Rht-B1a* > *Rht-B1b* > Béres > *Rht-B1c*) indicate that fructans may be important for cold stress responses in the studied ‘April Bearded’ genotypes, though their relatively low amounts do not substantially affect the high level of freezing tolerance of the best variety, Mv Béres. It is well documented that wheat accumulates the β(2,6)-linked levan type of fructans, and that this increase in fructan content is associated with the freezing tolerance [53,54]. Unlike oat genotypes, for instance, fructans with a high degree of polymerisation (DP > 6), were more closely correlated with freezing tolerance in wheat than low-DP fructans [55].

High data loads arising from liquid chromatography—high resolution mass spectrometry-based untargeted metabolomics—are generally subjected to reduction at any possible step of data evaluation [56], e.g., by limiting the chromatographic retention time window to be considered. While these efforts can be legitimated by substantive reasons (for example, to exclude compounds eluting close to the dead volume), neglecting one out of the two possible ionisation modes is also common. However, such an approach fundamentally derogates metabolomics information as a significant part of molecular entities can be detected only in one ionisation mode. Accordingly, our study covered both modes in order not to miss any relevant metabolite.

The present work also indicated that the applicability of PCA plotting should be evaluated in terms of the mass spectrometry analysis mode, i.e. negative or positive ionization. Although only 2461 molecular entities were detected in negative ion mode, they offered a clearer discrimination of all four genotypes and the treatment days than the 11,064 entities obtained in positive ion mode. Even when the molecular entities detected in positive ion mode were filtered to focus on the most abundant and significantly discriminating molecules, such as polyamine derivatives, the PCA plots were still not as clear and straightforward as in negative mode. Consequently, the complexity of the molecules detected in dwarf wheat samples in positive ion mode could not be directly linked to the genotypes or the time elapsing after exposure to cold stress.

## 4. Materials and Methods

### 4.1. Plant Materials, Growth Conditions and Freezing Test

The bread wheat (*Triticum aestivum* L.) genotypes used in the study were the Hungarian winter semi-dwarf variety “Mv Béres”, the facultative tall UK variety ‘April Bearded’, and two near-isogenic lines (NILs) in the background of ‘April Bearded’—one semi-dwarf and one extremely dwarf, differing in the alleles at the locus *Rht-B1* on chromosome 4B. The mutant alleles *Rht-B1b* (formerly *Rht1* gene) and *Rht-B1c* (formerly *Rht3* gene) were introduced into ‘April Bearded’ (possessing the wild type allele *Rht-B1a*) by crosses with accessions carrying the corresponding mutations, followed by repetitive backcrosses with recurrent selection for GA insensitivity [57]. These lines have been produced after six backcrosses and are considered to be near-isogenic, so the differences between lines within the background of variety ‘April Bearded’ could be attributed to differences at the Rht-B1 locus, although effects due to allelic variations at neighbouring loci are also possible as long as the exact length of the *Rht*-bearing chromosome segments is unknown. 

The facultative variety can be grown as either spring or winter wheat, and has low vernalization requirements and poor frost tolerance. Seed samples from the *Rht* NILs were provided by the Leibniz Institute of Plant Genetics and Crop Plant Research (IPK-Gatersleben), Germany, and multiplied in the experimental field of the Institute of Plant Physiology and Genetics (Sofia, Bulgaria). The Mv Béres winter wheat variety was bred in Martonvásár (Agricultural Institute, Centre for Agricultural Research, Hungary), which also carries the mutant allele *Rht-B1b* [58]. 

Approx. 25 seeds were sown for metabolite analysis, and 40 for freezing tests. In 110 mm diameter plastic pots containing a 3:1 (v:v) mixture of loamy soil and sand, they were grown for 10 d (to the 3-leaf stage) in a PGR-15 growth chamber (Conviron Ltd., Winnipeg, MB, Canada) at 20/18 °C (day/night) with a 16 h/8 h light/dark period, 75% relative humidity, and 250 μmol m^−2^ s^−1^ photosynthetic photon flux density (PPFD) at the leaf level, provided by metal halide lamps. Plants differing from the average size were removed. Low temperature hardening was carried out in a chamber of the same type at a constant 5 °C for 12 days. Samples for biochemical analysis were taken from the middle parts of the youngest fully developed leaves in the middle of the light period on the 0, 2nd, 7th and 12th days of the hardening period.

The freezing test was carried out as described earlier [59]. Briefly, cold-hardened seedlings were put in a frost chamber (ProfiMaster ECU 0520-1; National Lab GmbH, Mölln, Germany; H560 × W360 × D280 mm) for 1 d at −10 °C or −11 °C in the dark. In order to minimise the error from temperature inhomogeneity inside the frost chamber, the pots involved in freezing tests were divided into four sections (Figure 1), so that all four genotypes could be sown in each pot for simultaneous testing. After freezing, frozen seedlings were cut off at ground level, and the regrowth of the surviving plants was evaluated after 1–2 weeks under normal growth conditions (20/18 °C). The survival percentage was estimated by visual evaluation, counting the viable seedlings for all the genotypes in a pot (Figure 1A). Six pots were used for this calculation.

All the chemicals used in the experiments were purchased from Sigma-Aldrich-Supelco-Merck (Merck KGaA, Darmstadt, Germany).

### 4.2. Determination of Free Polyamine and 1,3-Diaminopropane (DAP)

Polyamines and DAP were determined according to Smith and Davies [60] and Szalai et al. [61]. For analyses, leaf samples were homogenized with 0.2 M ice-cold perchloric acid and allowed to stand for 20 min on ice. After centrifugation at 10,000× *g* and 4 °C for 20 min, the supernatant was used for the analyses. The polyamines, namely cadaverine (CAD), putrescine (PUT), spermidine (SPD) and spermine (SPM), together with DAP, the terminal catabolite product of SPD and SPM, were separated via HPLC using an Alliance W2690 separation module (Waters, Milford, MA, USA) on a reverse phase column (Kinetex C18, 5 μ, 100 × 4.6 mm, Phenomenex; Torrance, CA, USA) and analysed as dansylated derivatives using a W2474 scanning fluorescence detector (Waters, Milford, MA, USA) with excitation at 340 nm and emission at 515 nm.

### 4.3. Determination of Ascorbate Peroxidase Activity

Ascorbate peroxidase (APX; EC 1.11.1.11.) activity was determined spectrophotometrically, as described earlier [62]. Leaf samples (0.5 g) were homogenised in 2.5 mL of ice-cold Tris buffer (0.5 M, pH 7.5) containing 3 mM MgCl_2_ and 1 mM EDTA. The enzyme activity was determined as a decrease in absorbance at 290 nm with a Shimadzu UV-160A multiwavelength spectrophotometer (Shimadzu, Kyoto, Japan) using 50 µL plant extract in Tris buffer (pH 7.8) in the presence of 0.2 M 5.625 mM ascorbic acid. The reaction was started with 0.042% hydrogen peroxide in a final volume of 2250 µL reaction mixture. Enzyme activity (in nkatal) was calculated according to this: 1000 × (V_total_/V_sample_) × ε^−1^ × ΔA_(290nm)_ × dilution × time^−1^ (V_total_: total volume of the reaction mixture; V_sample_: volume of the plant extract; ε: extinction coefficient of ascorbate, 2.8 mM^−1^ cm^−1^; ΔA_(290nm)_: decrease of the absorbance at 290 nm; dilution: can be calculated according to the sample preparation; time: duration of the measured decrease of absorbance in seconds).

### 4.4. Quantification of Selected Flavonols

Flavonols, namely rutin, myricetin, quercetin and kaempferol, were quantified according to Gondor et al. [63] using 0.5 g wheat leaves. Leaf samples were ground in liquid nitrogen in a mortar and pestle in the presence of quartz sand. The tissue powder was transferred to a centrifugation tube and mixed with 2 mL of 70% methanol. The extract was centrifuged at 10,000× *g* for 10 min. The pellet was resuspended in 2 mL of 90% methanol, vortexed and centrifuged as above. The methanol content was evaporated at room temperature under a vacuum. One millilitre of 5% (*w*/*v*) trichloroacetic acid was added to the residual aqueous phase, and the mixture was centrifuged at 10,000× *g* for 10 min. The supernatant was gently partitioned twice against 1.5 mL of a 1:1 (*v*/*v*) mixture of ethyl acetate/cyclohexane. The upper organic layer, which contained the free phenolic portion, was evaporated to dryness under vacuum. The evaporated samples were resuspended in 500 µL 15% HPLC grade acetonitrile and detected after separation on a Synergi Fusion RP 80A 150 × 4 mm reverse phase column (Alliance W2690; Waters, Milford, MA, USA) with a photodiode array detector in the range of 230–300 nm. Flavonols were quantified at their maximum (quercetin: 220 nm; myricetin: 253 nm; kaempferol and rutin: 265 nm) using external calibration curves.

### 4.5. Gene Expression Analysis

For gene expression analysis, the second fully developed leaves were collected. Total RNA isolation and cDNA synthesis were performed as described by Tajti et al. [64]. In detail: Total RNA was extracted from fully developed leaves using TRI Reagent^®^. The samples were treated with DNase I and cleaned with a Direct-zol™ RNA MiniPrep Kit (Zymo Research, Irvine, CA, USA) according to the manufacturer’s instructions. The quality and integrity of RNA was monitored using an agarose gel and the samples were quantified with a Nanodrop 2000 spectrophotometer (Thermo Fisher Scientific Inc., Wilmington, MA, USA). Total RNA (1000 ng) was reverse transcribed by using M-MLV Reverse Transcriptase (Promega Corporation, Madison, WI, USA) and oligo(dT)18 (Thermo Fisher Scientific) 1 µL of 2-fold diluted cDNA, gene-specific primers and housekeeping primers (Table 2), PCRBIO SyGreen Mix (PCR Biosystems, London, UK) and CFX96 Touch™ Real-Time PCR Detection System (Bio-Rad, Hercules, CA, USA) were used for quantitative real-time PCR reaction. Melt curve analysis was also performed to confirm the presence of a single PCR product. The relative gene expression values were determined with the 2^−ΔΔCt^ method [65]. For normalisation the Ct values of the housekeeping gene, Ta30797, encoding phosphogluconate dehydrogenase, were used [66]. All reactions were performed in triplicate using three biological and three technical repetitions.

### 4.6. Metabolomics Analyses

#### 4.6.1. Sample Preparation

Five parallel leaf samples from each sample group (i.e., collection on the 0, 2nd, 7th and 12th days after the beginning of cold hardening and from four genotypes) were ground in liquid N_2_, and 0.2 g subsamples were weighed into 2.0 mL safety Eppendorf tubes with the addition of 1.0 mL of 40% (*v*/*v*) methanol. After vortex mixing, the tubes were inserted into a pre-cooled (−18 °C) tube holder and the extraction was carried out in a 1600 MiniG^®^—Automated Tissue Homogenizer and Cell Lyser (SPEX; Rickmansworth, UK) for 3 min at 1500 rpm. The samples were then centrifuged at 15,000× *g* for 15 min at +4 °C, after which the supernatants were recovered and placed in separate Eppendorf tubes. The residues were re-extracted with 1.0 mL of 90% (*v*/*v*) methanol in a MiniG^®^ instrument (3 min), followed by centrifugation as described above. The matching supernatants were pooled and used for GC-MS and LC-MS analysis.

#### 4.6.2. Untargeted Gas Chromatography-Mass Spectrometry (GC-MS) Analysis

For GC-MS analysis, 15 µL of 1 mg mL^−1^ ribitol was added as an internal standard to 1.0 mL methanolic extract and an aliquot (200 µL) was dried under a vacuum. Derivatization was performed with 40 µL methoxyamine hydrochloride dissolved in pyridine (20 mg mL^−1^), with incubation for 90 min at 37 °C. After this, 60 µL N-trimethylsilyl-N-methyl trifluoroacetamide was added and the sample was incubated for an additional 30 min at the same temperature. The samples were transferred to vials and 1.0 µL was injected into a LECO Pegasus 4D GCxGC TOFMS (LECO, St Joseph, MI, USA) equipped with a 30 m capillary column (Rxi-5MS phase) and a 1.5 m column (Rxi-17Sil MS phase) in a split mode. The temperature of the inlet was 230 °C, and the transfer line and ion source were kept at 250 °C. The carrier gas was He and a constant flow rate (1 mL min^−1^) was used. After 70 °C for 3 min, the temperature was increased to 320 °C at a rate of 7 °C min^−1^ where it was maintained for 5 min with a 3.25 s modulation period in 2D GC mode. GC analysis and data processing were carried out using ChromaTOF 4.72 software. External standards, Kovats retention index data, NIST and LECO-Fiehn metabolomics library databases were used to identify the compounds.

#### 4.6.3. Untargeted Liquid Chromatography-Mass Spectrometry (LC-MS) Analysis

For LC-MS analysis, aliquots of the combined supernatants were diluted either 1:2 with water containing 0.15% (*v*/*v*) formic acid (for C_18_ chromatography) or 1:4 with acetonitrile (for HILIC/hydrophilic interaction liquid chromatography). After dilution, the sample solutions were filtered through 0.22 µm pore-sized disposable PTFE syringe filters and injected into the LC-MS set-up. A Vion ion mobility quadrupole time-of-flight mass spectrometer (Waters, Milford, MA, USA) equipped with a UniSpray (Waters, Milford, MA, USA) ion spray source was applied. Chromatographic elution was provided by an Acquity UPLC I-Class system (Waters, Milford, MA, USA) using either a BEH-C_18_ reversed phase (RP) UPLC column (100 mm × 2.1 mm × 1.7 µm; Waters, Milford, MA, USA) or a SeQuant ZIC-cHILIC HPLC column (100 mm × 2.1 mm × 3.0 µm; Merck-Sigma Group, Schnelldorf, Germany). The UniSpray ion source was used both in positive and negative ionisation modes, either with MS^E^ or with MS^E^-MSMS/DDA functions. The instrument was controlled using UNIFI software (version 1.9.4; Waters). The related instrumental parameters are described in Table S4. For the quantitative estimation of discriminating flavonols, apigenin was obtained from the Merck-Sigma group and its UV chromatogram was recorded at λ = 334 nm. Concentrations of flavonols were estimated by normalisation of their UV absorption data at λ = 334 nm (for more abundant species) or on the basis of their MS intensity (for less abundant species where UV trace couldn’t be adequately obtained). For quantitative estimation of the concentration of discriminating conjugated polyamines, feruloylputrescine (>97% purity) was obtained from BOC Sciences (Shirley, NY, USA) and its MS response was used for normalisation.

#### 4.6.4. Multivariate Statistical Analysis of LC-MS Data

The Vion UNIFI LC-MS data were processed with the Progenesis QI (Version 27.26.1020; Nonlinear Dynamics, Quayside, Newcastle Upon Tyne, UK) and EZinfo (version 3.0.3; UMetrics AB, Umeei, Sweden) software packages.

In the Progenesis QI work step, the datasets were first aligned according to the default settings. Peak picking was carried out with the following settings: sensitivity: “automatic”; minimum chromatographic peak width: 0.03 min (in positive mode) or 0.04 min (in negative mode); retention time window: 0.55–15 min (in positive mode) or 0.60–15 min (in negative mode); high energy limits: 0.2% base peak; adducts concerned: M-H, 2M-H, M-H_2_O-H, M+Na-2H and M+H, M-H_2_O+H, M+Na, M+K, M+NH_4_ and 2M+H. With these settings, 2461 and 11,064 entities were found in negative and positive ion mode, respectively, and were subjected to ANOVA and Principal Component Analysis (PCA). For group difference calculations, the PCA data were exported to EZinfo, where pairwise group differences were visualised in an S-plot arrangement. Data (=compounds) were considered significant and transferred back to Progenesis QI for identification if (i) the related fold change exceeded 2.0, (ii) their p[1] loadings were higher than |0.1| and/or (iii) their p(corr)[1] correlation values were higher than |0.95|. In the case of oligohexoses, where peak picking failed because of the highly complex adduct formation (i.e., M+H, M+H+K, M+Na, M+2Na, M+Na+K, M+K, M+2K, M+NH_4_), the ion intensities of the most abundant adducts (M+Na and M+2Na) were used for pairwise *t*-test comparison of the wheat genotypes.

The following settings were applied for compound identification: precursor tolerance: 5 ppm; fragment tolerance: 10 ppm; isotope similarity: 95%; elemental composition: H 0–180, C 0–100, N 0–10, O 0–100, P 0–3, S 0–2. The following data sources were considered: Biosynth Carbosynth, ChEBI, ChEMBL, Extrasynthese, FDA, FAO, FooDB, HMDB, KEGG, LipidMAPS, MassBank, Molbank, Nature Chemistry, Nature Chemical Biology, NIST, NIST Chemistry WebBook, Phenol Explorer and PlantCyc. The assignment of statistically differentiating components (polyamines and flavonoids) was based on their accurate mass, MS/MS fragments and chromatographic elution order according to Cavaliere et al. [71], Ferreres et al. [72], Shao et al. [73], Pereira et al. [74], da Silva and de Oliveira [75], Nikolic et al. [76], Lee et al. [77], Kage et al. [78], Gorzolka et al. [79] and Dong et al. [80]. Figure S7 presents all the related high-resolution MS and MS/MS spectra. Oligohexoses (fructans) were assigned according to accurate mass data only, and they were characterised only by the number of hexose moieties. Compounds found to be significant in group difference analyses in either ionisation mode were sorted by their maximum abundance, and entries exceeding either 600 cps (in negative mode) or 850 cps (in positive mode), i.e. the experimentally determined limits for reliable MS/MS acquisition, were individually and manually checked for redundancies (e.g. because of incorrect isotopologue alignment) in the LC-MS spectra. Non-redundant entries were subjected to directed data acquisition (DDA)—MS/MS fragmentation in both negative and positive mode when applicable.

### 4.7. Statistical Analysis

The whole experimental design was repeated at least three times, and a representative set of experiments is given here. In each set of experiments, the biochemical data represented the average of five measurements. The data were statistically evaluated using the standard deviation and *t*-test methods in the Microsoft Excel program.

## 5. Conclusions

Present results show that mutant alleles *Rht-B1b* and *Rht-B1c* may negatively affect the freezing tolerance in ‘April Bearded’ facultative wheat plants. However, several cold-related processes exhibited similar changes during the cold hardening period in these genotypes. High resolution MS and MS/MS based untargeted metabolomics revealed a series of differentiating compounds, especially coumaroylated and feruloylated poliamines and apigenine derivatives, that showed a remarkably different time profile compared to the stress-induced fructans. The mechanisms of frost tolerance might be different for a typical winter variety (Mv Béres) than for a facultative variety. Although oligosaccharides are able to differentiate between them, it is unlikely that they play an important role in Mv Béres, which may use other strategies. Compared to flavonoids, the polyamine derivatives exhibited very diverse patterns, and the accumulation of these compounds correlated with the level of freezing tolerance of the ‘April Bearded’ lines. However, in Mv Béres, which had the highest level of tolerance, the accumulation of these compounds was much less pronounced. Obviously, the interaction between the major developmental genes, frost tolerance genes and the Rht genes merits further deep exploration.

## Figures and Tables

**Table 1:** Statistically differentiating polyamines and flavones detected in the four wheat genotypes with reversed phase separation. (*) indicates no more definite synonym can be provided (*polyamine derivatives: italics*; **flavones: bold**).

Retention Time, min	Tentative Identification	Elemental Composition (Neutral)	Theoretical *m*/^z^ [M + H]^+^	Experimental *m*/^z^ [M + H]^+^	Difference, ppm	Theoretical *m*/^z^ [M − H]^−^	Experimental *m*/^z^ [M − H]^−^	Difference, ppm	Molecule Group
3.08	*Isoferuloyl putrescine*	C_14_H_20_N_2_O_3_	265.15467	265.15486	0.72	-	-	-	Polyamine derivative
3.48	*(Iso)feruloyl-2-hydroxyputrescine*	C_14_H_20_N_2_O_4_	281.14958	281.14955	−0.11	-	-	-	
3.49	*p-Coumaroylhydroxyagmatine*	C_14_H_20_N_4_O_3_	293.16082	293.16071	−0.38	-	-	-	
3.96	*Feruloyl putrescine*	C_14_H_20_N_2_O_3_	265.15467	265.15451	−0.60	-	-	-	
4.13	*Isoferuloylagmatine*	C_15_H_22_N_4_O_3_	307.17647	307.17661	0.46	-	-	-	
4.26	*Feruloylhydroxyagmatine*	C_15_H_22_N_4_O_4_	323.17138	323.17120	−0.56	-	-	-	
4.65	*p-Coumaroylagmatine*	C_14_H_20_N_4_O_2_	277.16590	277.16578	−0.43	-	-	-	
5.41	*Feruloylagmatine*	C_15_H_22_N_4_O_3_	307.17647	307.17667	0.65	-	-	-	
5.58	**Lutonarin**	C_27_H_30_O_16_	611.16066	611.16099	0.54	609.14611	609.14620	0.15	Luteolin derivative
6.00	**Vicenin-2**	C_27_H_30_O_15_	595.16575	595.16550	−0.42	593.15119	593.15115	−0.07	Isovitexin derivative
6.26	**Lucenin-1/3**	C_26_H_28_O_15_	581.15010	581.15050	0.69	579.13554	579.13497	−0.98	Luteolin derivative
6.56	**Isoorientin-2″-O-glucoside**	C_27_H_30_O_16_	611.16066	611.16080	0.23	609.14611	609.14659	0.79	Luteolin derivative
6.65	**Isovitexin-7-O-glucoside (saponarin)**	C_27_H30O_15_	595.16575	595.16581	0.10	593.15119	593.15088	−0.52	Isovitexin derivative
6.87	**Isoorientin**	C_21_H_20_O_11_	449.10784	449.10758	−0.58	447.09329	447.09354	0.56	Luteolin derivative
6.99	**Isoschaftoside**	C_26_H_28_O_14_	565.15518	565.15527	0.16	563.14063	563.14050	−0.23	Apigenin derivative
7.04	**Luteolin-6c-rutinoside (Isoorientin-6-rhamnoside)**	C_27_H_30_O_15_	595.16575	595.16599	0.40	593.15119	593.15087	−0.54	Luteolin derivative
7.09	**Isoscoparin-7-O-glucoside**	C_28_H_32_O_16_	625.17631	625.17585	−0.74	623.16176	623.16144	−0.51	Methylluteolin derivative
7.57	**Isovitexin-2″-O-glucoside**	C_27_H_30_O_15_	595.16575	595.16536	−0.66	593.15119	593.15155	0.61	Isovitexin derivative
8.06	**Isovitexin**	C_21_H_20_O_10_	433.11292	433.11284	−0.18	431.09837	431.09853	0.37	Isovitexin
8.07	**Isoscoparin-2″-O-glucoside**	C_28_H_32_O_16_	625.17631	625.17628	−0.05	623.16176	623.16157	−0.30	Methylluteolin derivative
8.10	**Isovitexin-2-O-rhamnoside**	C_27_H_30_O_14_	579.17083	579.17094	0.19	577.15628	577.15639	0.19	Isovitexin derivative
8.62	C-hexosyl-chrysoeriol O-rhamnoside *	C_28_H_32_O_15_	609.18140	609.18146	0.10	607.16684	607.16634	−0.82	Methylluteolin derivative
9.67	C-hexosyl-luteolin O-feruloylpentoside *	C_36_H_36_O_18_	757.19744	757.19699	−0.59	755.18289	755.18230	−0.78	Luteolin derivative
11.09	**Isovitexin 2″-O-(6‴-feruloyl)glucoside**	C_37_H_38_O_18_	771.21309	771.21326	0.22	769.19854	769.19858	0.05	Isovitexin derivative
13.54	**3′-O-methylluteolin (chrysoeriol)**	C_16_H_12_O_6_	301.07066	301.07079	0.43	299.05611	299.05627	0.54	Methylluteolin

**Table 2:** Primer sequences used for the gene expression studies.

Gene Name	Primer Sequences (5′ → 3′)		Reference
*Ta30797*	Forward	GCCGTGTCCATGCCAGTG	Paolacci et al., 2009 [66]
	Reverse	TTAGCCTGAACCACCTGTGC	
*SOS2*	Forward	GAAAACCTGCTTCTTGATTCACG	Darkó et al., 2017 [67]
	Reverse	GCTGCAGATCCATCATAGCC	
*R2R3MYB*	Forward	TTGGTCGTCGATCCTCCACAG	Boldizsár et al., 2016 [68]
	Reverse	GAGGAGAAGAAGGCGGAGGTG	
*COR14*	Forward	GAGAAGGCGAAGCAGGCGAC	Campoli et al., 2009 [69]
	Reverse	TTGCTCACATCCTCAACCGC	
*CBF14*	Forward	CATGGAGTCGCCGGACACCAGACC	Knox et al., 2008 [70]
	Reverse	GCCCTCCCCAAAAATAGACAGCGGAG	
*SS*	Forward	CCGACAAGGAGAAGTATG	Zeng et al., 2011 [30]
	Reverse	CGAGTTCACTAACATTCAC	
*wpi6*	Forward	TCTTCCTGCGCTACAAACTC	NCBI Reference Sequence: AB030210.1
	Reverse	AACTACCAGCACGTACACCG	

## Data Availability

All relevant data can be found within the manuscript and its supplementary materials.

## Supplementary Materials

The following are available online at https://www.mdpi.com/article/10.3390/ijms23147969/s1.
