# Supplementary material for: Mutations in Rht-B1 Locus May Negatively Affect Frost Tolerance in Bread Wheat
Source: Int J Mol Sci. 2022 Jul 19;23(14):7969. doi: 10.3390/ijms23147969 (PMC9324540; doi:10.3390/ijms23147969)

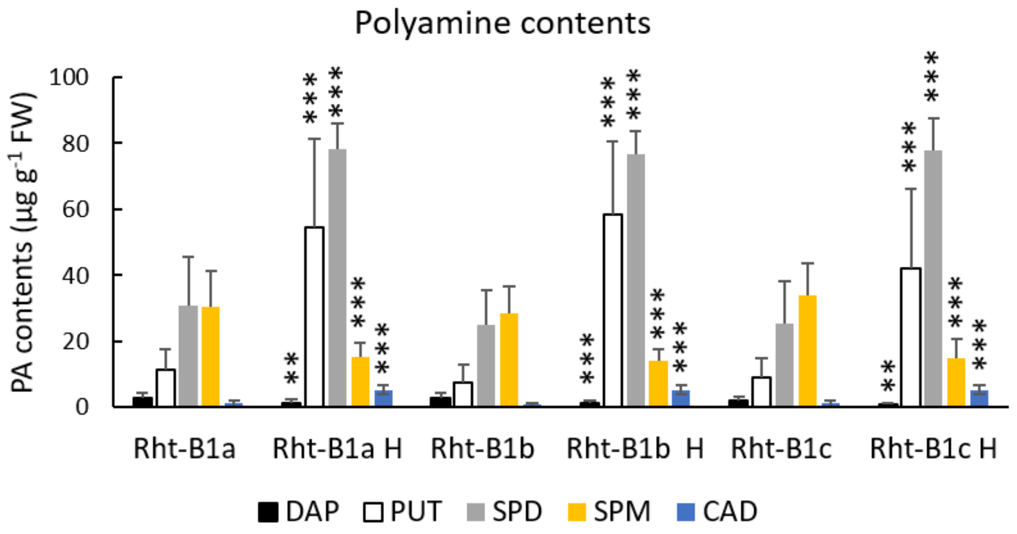

Figure S1. Contents of major free polyamines (PA), namely putrescine (PUT), spermidine (SPD), and spermine (SPM), and the degradation product 1,3-diaminopropane (DAP) in control and cold-hardened (H) young *Rht* wheat genotypes. Error bars indicate standard deviations. *,**,*** represent significant differences between control and cold-hardened plants at the $p < 0.05$, 0.01 and 0.001 levels, respectively.

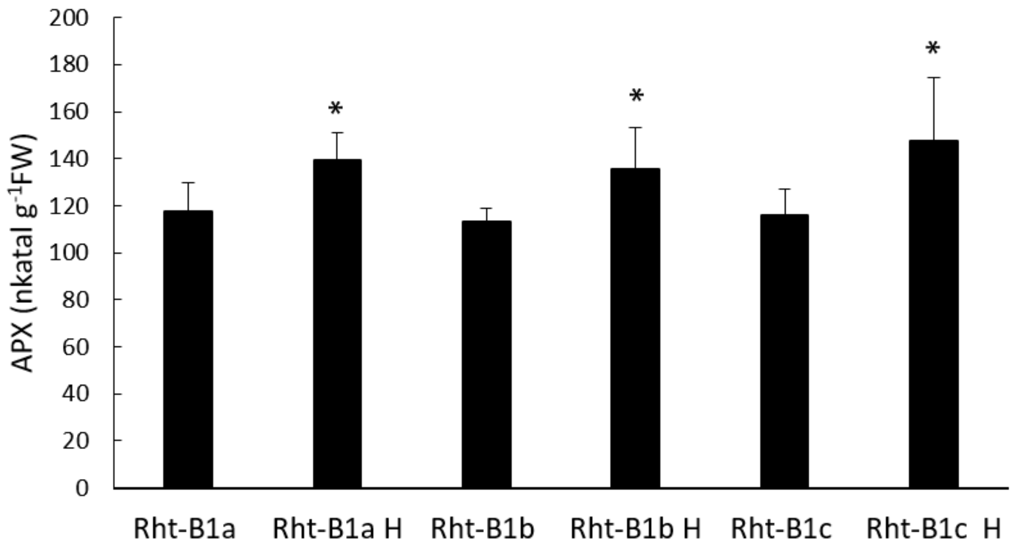

Figure S2. Activity of the ascorbate peroxidase enzyme in control and cold-hardened (H) young *Rht* wheat genotypes. Error bars indicate standard deviations. *,**,*** represent significant differences between control and cold-hardened plants at the $p < 0.05$, 0.01 and 0.001 levels, respectively.

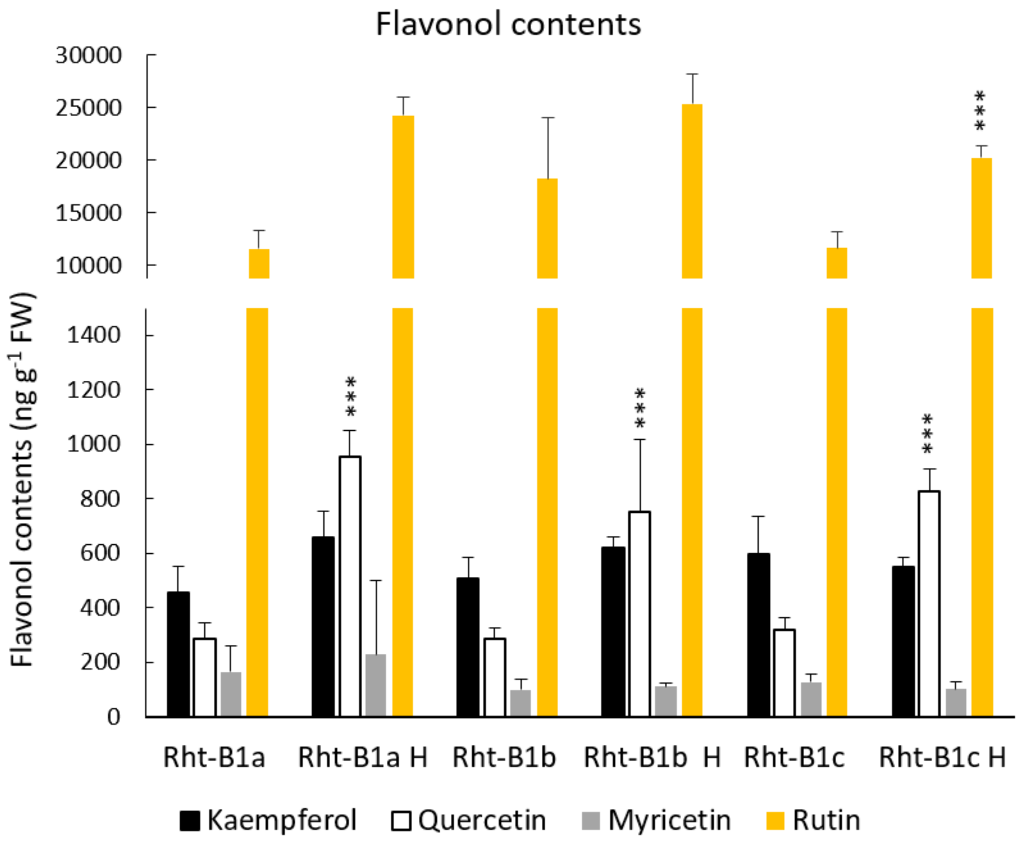

Figure S3. Contents of selected flavonols, namely kaempferol, quercetin, myricetin and rutin in control and cold-hardened (H) young *Rht* wheat genotypes. Error bars indicate standard deviations. *,**,*** represent significant differences between control and cold-hardened plants at the $p < 0.05$, 0.01, and 0.001 levels, respectively.

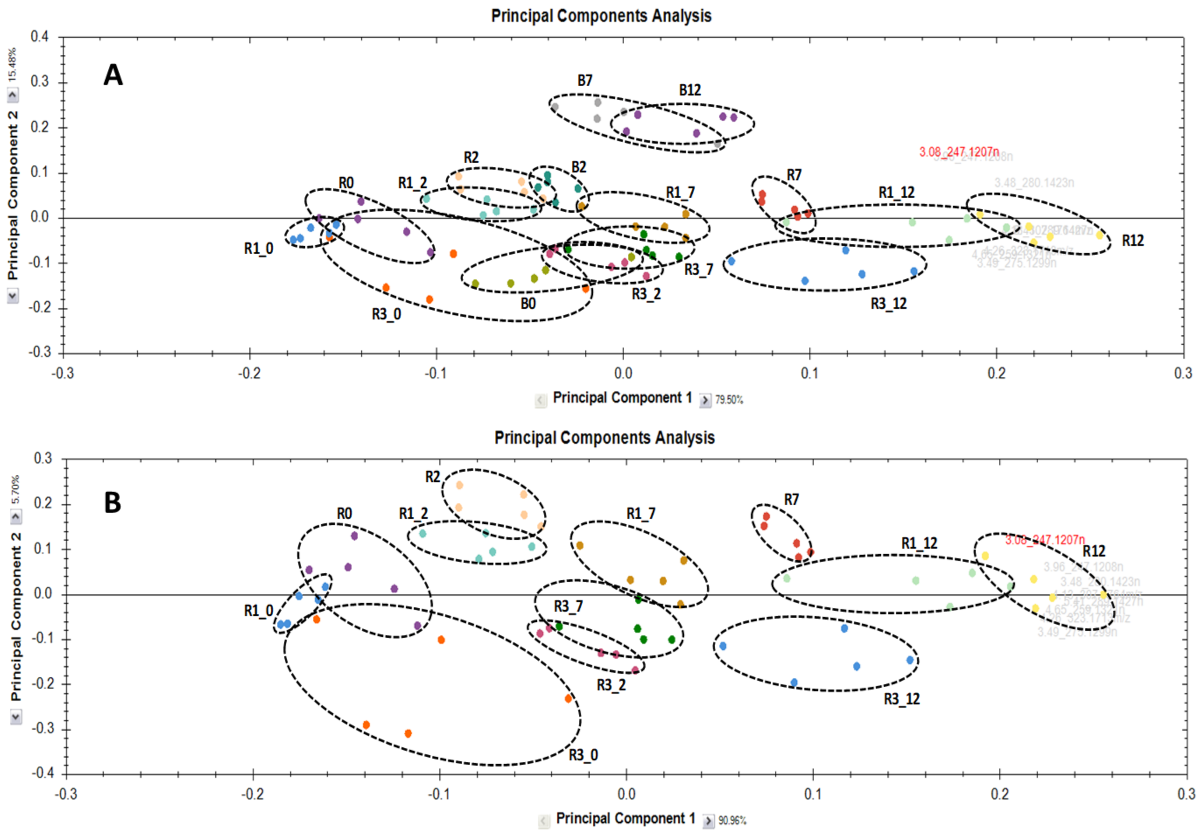

Figure S4. Principal component analysis (PCA) for eight polyamine derivatives (detectable in positive ion mode only) including (A) or without (B) the Mv Béres genotype. Legend: R0: *Rht-B1a* day 0; R2: *Rht-B1a* day 2; R7: *Rht-B1a* day 7; R12: *Rht-B1a* day 12; R1_0: *Rht-B1b* day 0; R1_2: *Rht-B1b* day 2; R1_7: *Rht-B1b* day 7; R1_12: *Rht-B1b* day 12; R3_0: *Rht-B1c* day 0; R3_2: *Rht-B1c* day 2; R3_7: *Rht-B1c* day 7; R3_12: *Rht-B1c* day 12; B0: Mv Béres day 0; B2: Mv Béres day 2; B7: Mv Béres day 7; B12: Mv Béres day 12

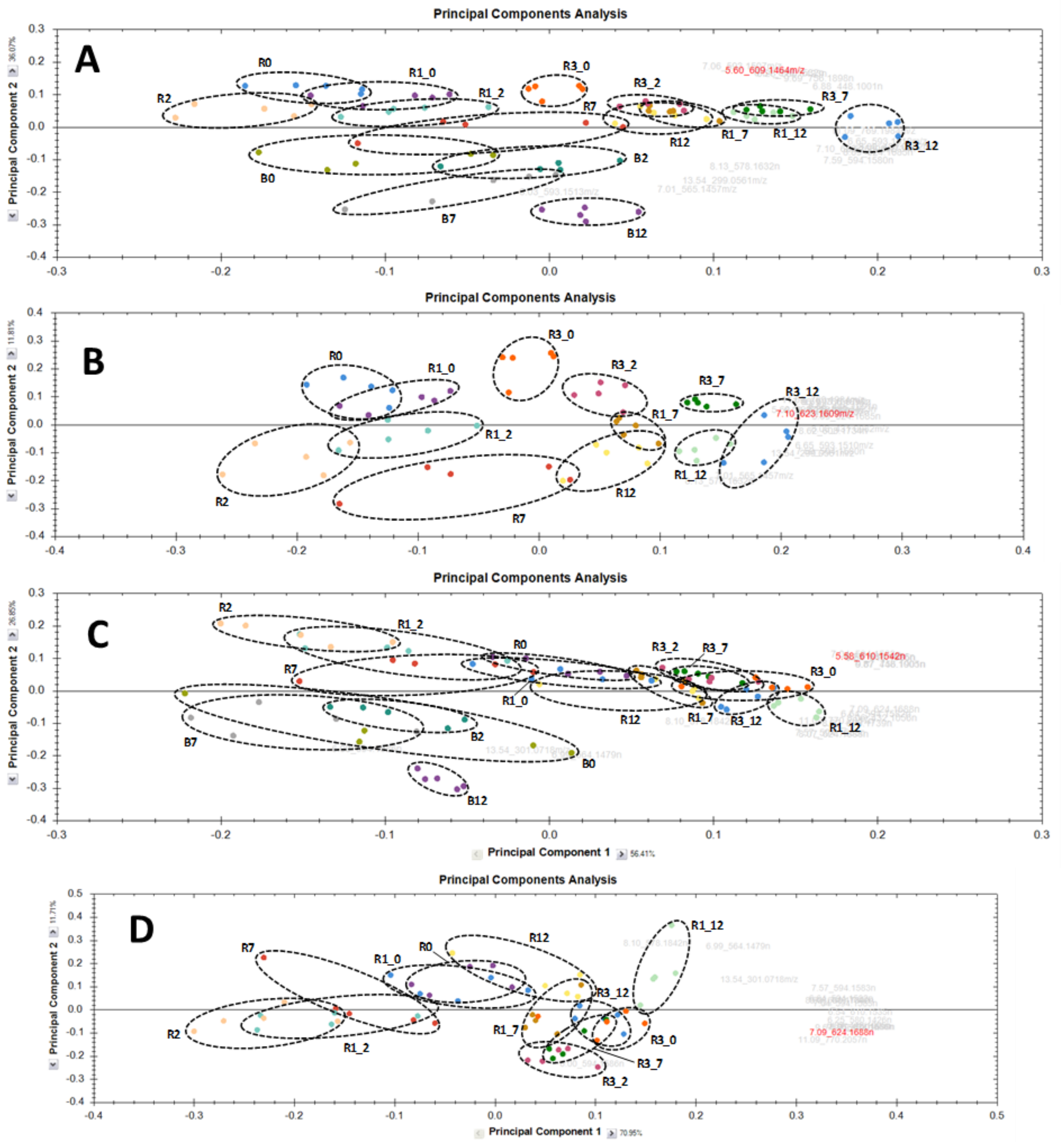

Figure S5. Principal component analysis (PCA) for 17 flavones including (A, C) or without (B, D) the Mv Béres genotype in ESI- (A, B) or ESI+ (C, D) mode. Legend: R0: *Rht-B1a* day 0; R2: *Rht-B1a* day 2; R7: *Rht-B1a* day 7; R12: *Rht-B1a* day 12; R1_0: *Rht*-B1b day 0; R1_2: *Rht-B1b* day 2; R1_7: *Rht-B1b* day 7; R1_12: *Rht-B1b* day 12; R3_0: *Rht-B1c* day 0; R3_2: *Rht-B1c* day 2; R3_7: *Rht-B1c* day 7; R3_12: *Rht-B1c* day 12; B0: Mv Béres day 0; B2: Mv Béres day 2; B7: Mv Béres day 7; B12: Mv Béres day 12

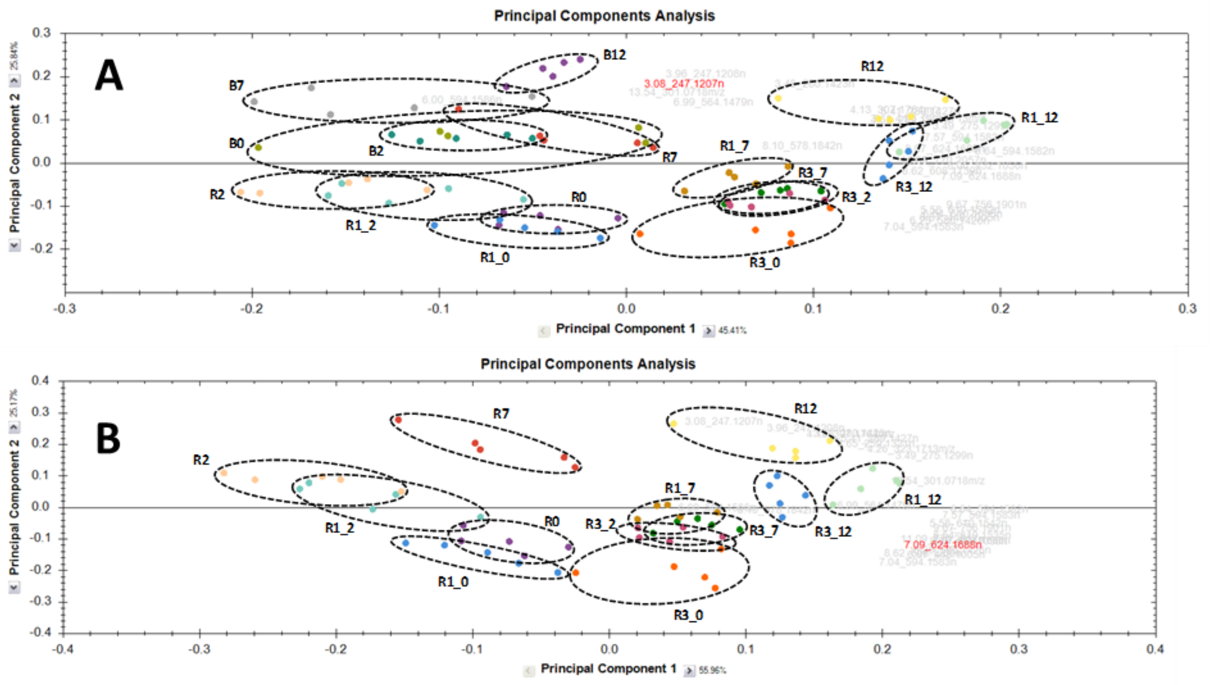

Figure S6. Joint principal component analysis (PCA) for eight polyamine derivatives and 17 flavones including (A) or without (B) the Mv Béres genotype in ESI+ mode. Legend: R0: *Rht-B1a* day 0; R2: *Rht-B1a* day 2; R7: *Rht-B1a* day 7; R12: *Rht-B1a* day 12; R1_0: *Rht-B1b* day 0; R1_2: *Rht-B1b* day 2; R1_7: *Rht-B1b* day 7; R1_12: *Rht-B1b* day 12; R3_0: *Rht-B1c* day 0; R3_2: *Rht-B1c* day 2; R3_7: *Rht-B1c* day 7; R3_12: *Rht-B1c* day 12; B0: Mv Béres day 0; B2: Mv Béres day 2; B7: Mv Béres day 7; B12: Mv Béres day 12

Figure S7. Related high resolution MS and MS/MS spectra

Compound name: isoferuloyl putrescine
ESI-MS mode: positive, Unispray ion source
m/z (experimental): 265.15486

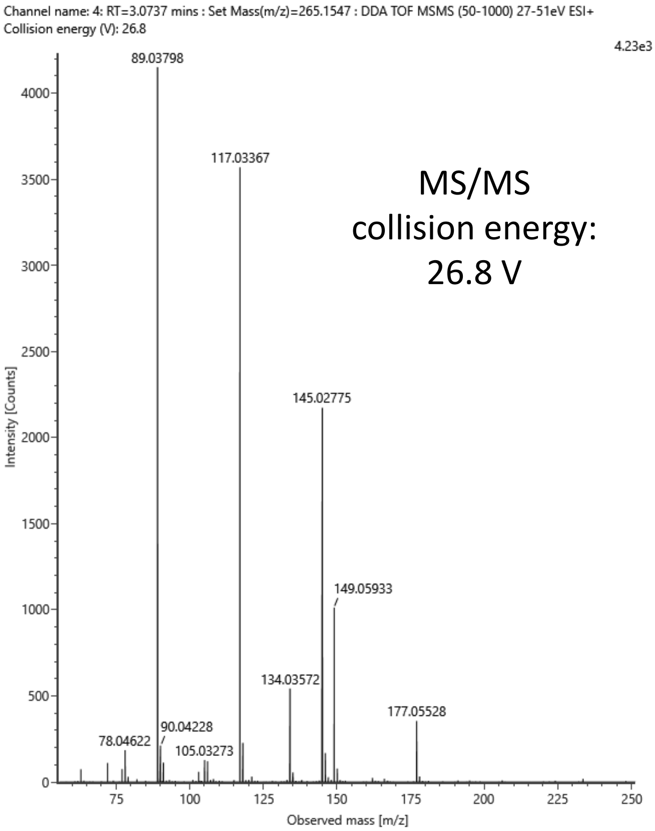

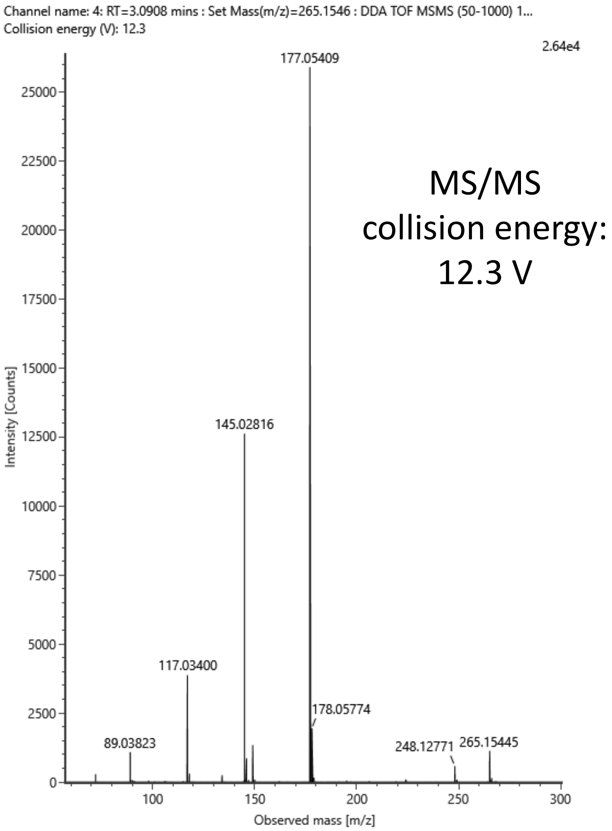

Compound name: (iso)feruloyl-2-hydroxyputrescine
ESI-MS mode: positive, Unispray ion source
m/z (experimental): 281.14955

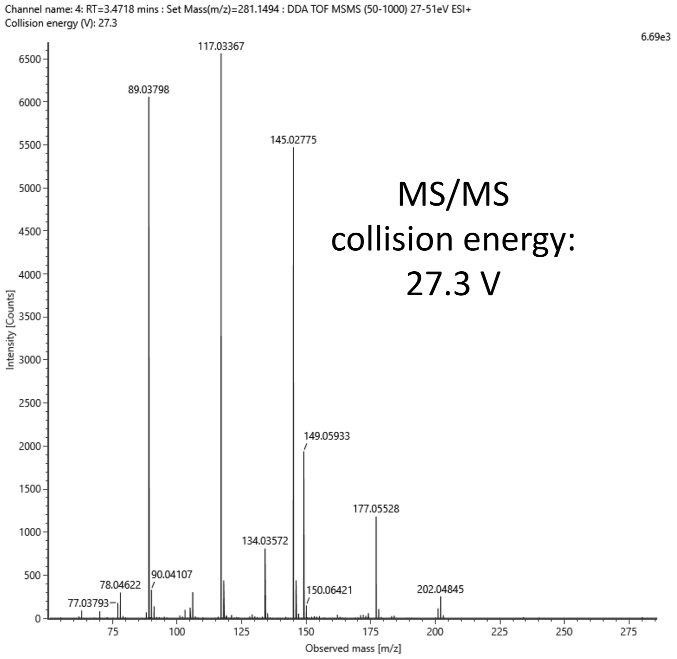

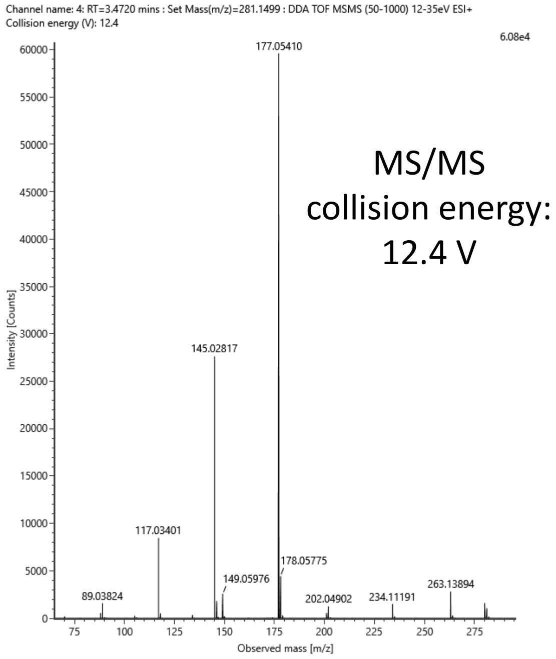

Compound name: p-coumaroylhydroxyagmatine
ESI-MS mode: positive, Unispray ion source
m/z (experimental): 293.16071

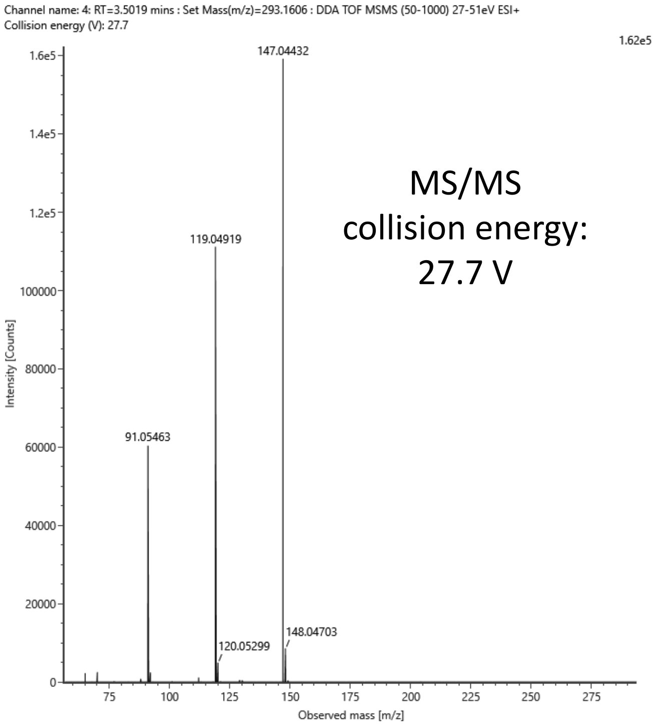

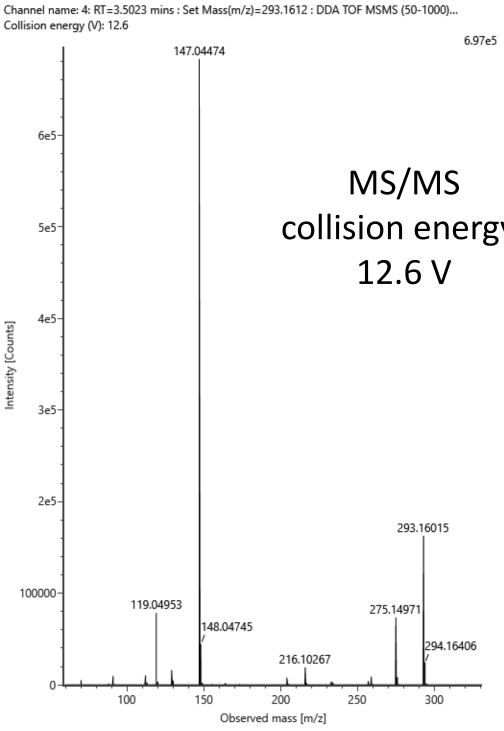

Compound name: feruloyl putrescine
ESI-MS mode: positive, Unispray ion source
m/z (experimental): 265.15451

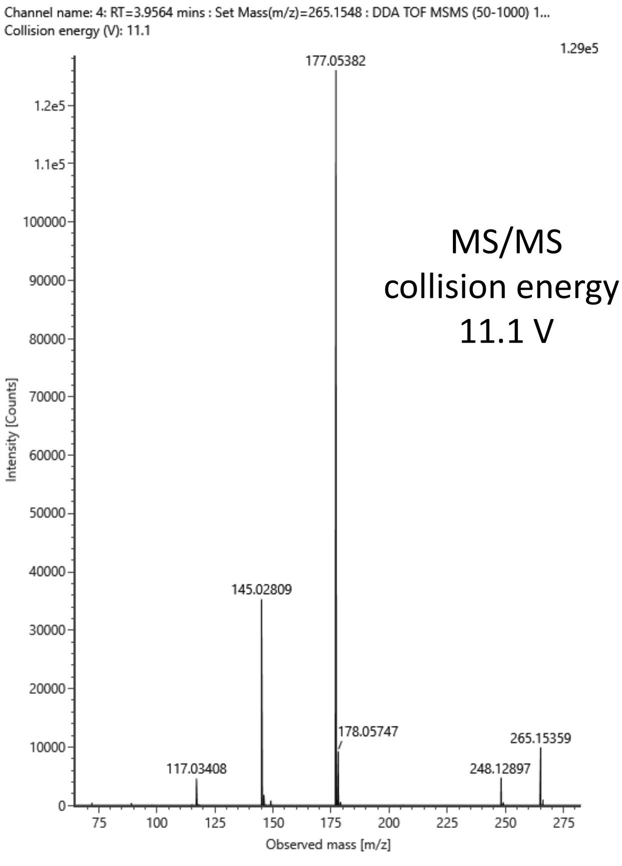

Compound name: isoferuloylagmatine
ESI-MS mode: positive, Unispray ion source
m/z (experimental): 307.17661

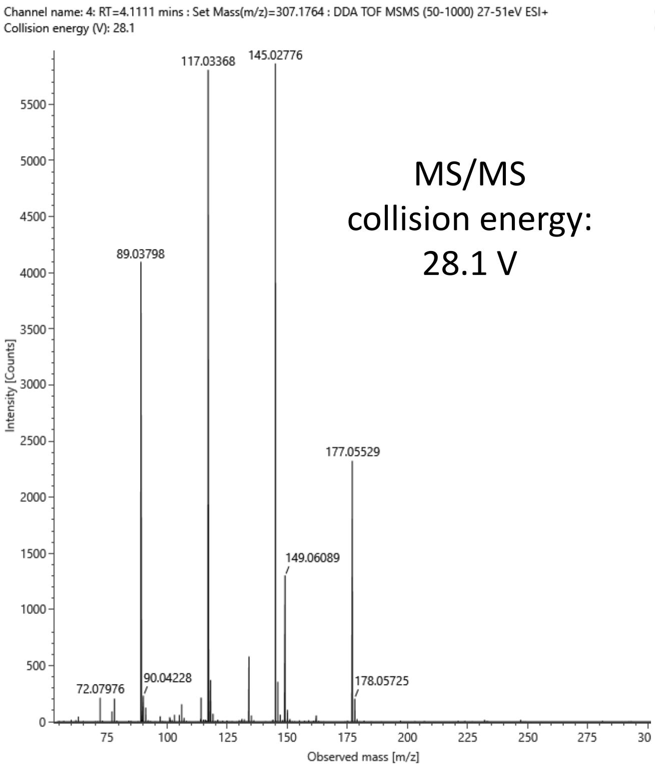

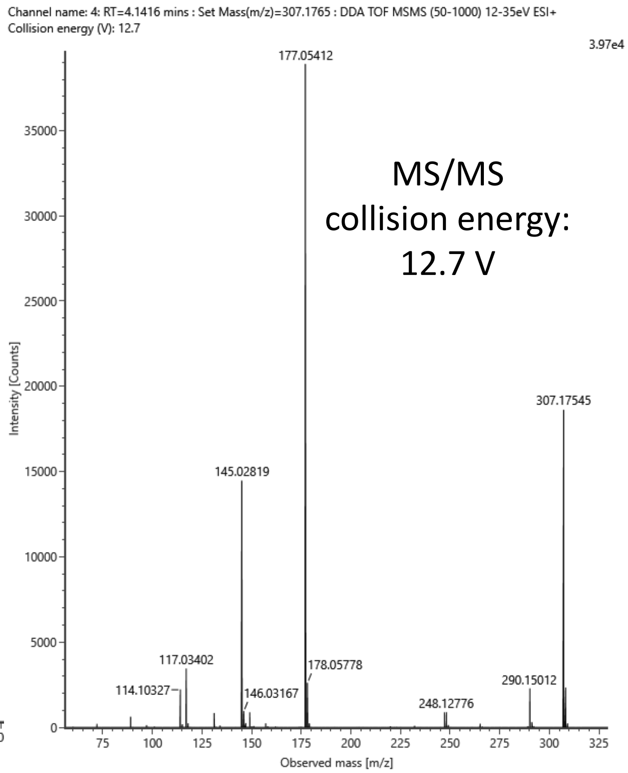

Compound name: feruloylhydroxyagmatine
ESI-MS mode: positive, Unispray ion source
m/z (experimental): 323.17120

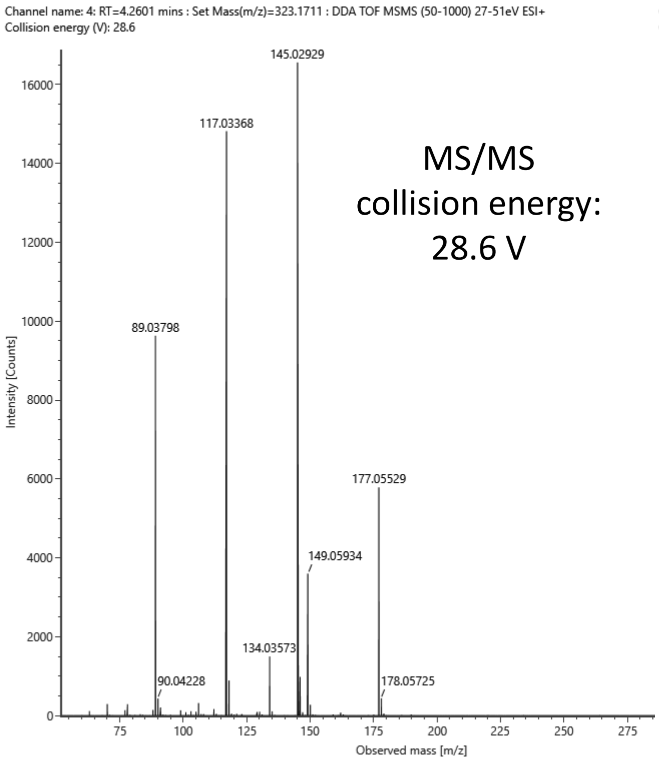

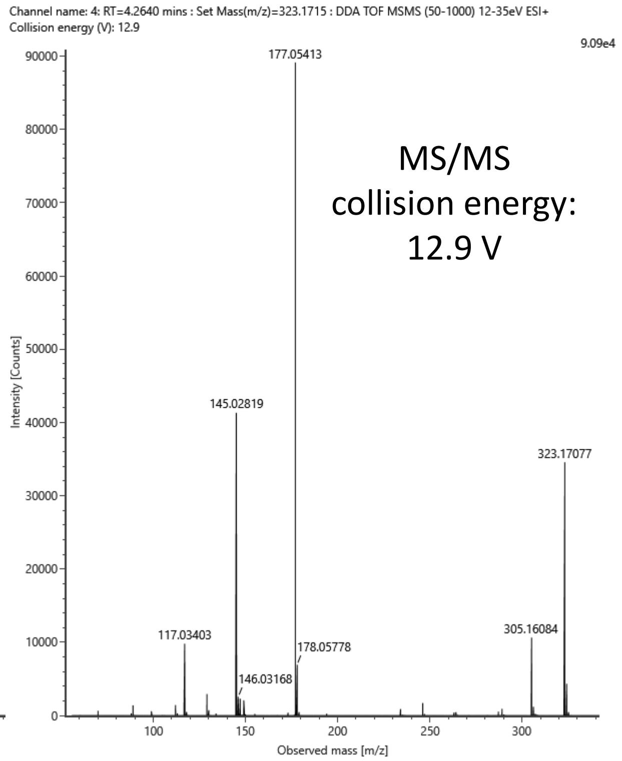

Compound name: p-coumaroylagmatine
ESI-MS mode: positive, Unispray ion source
m/z (experimental): 277.16578

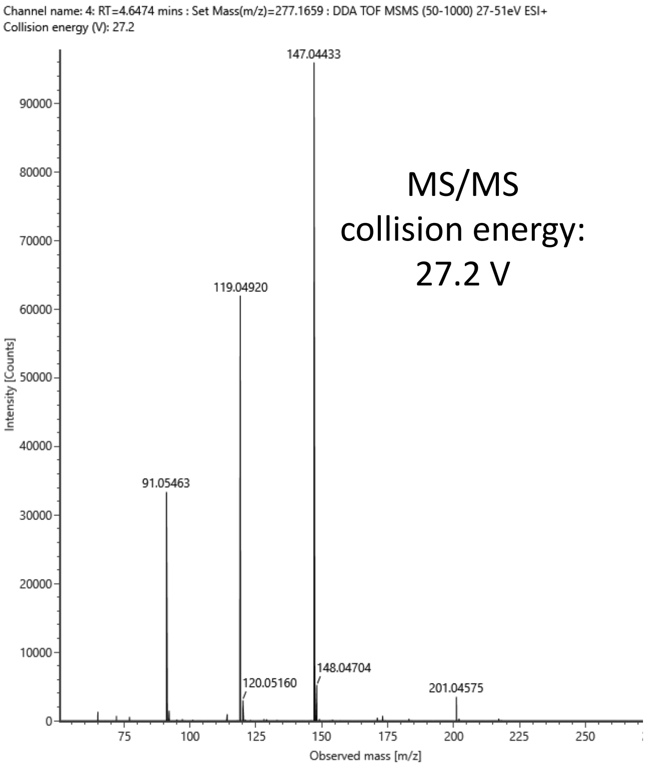

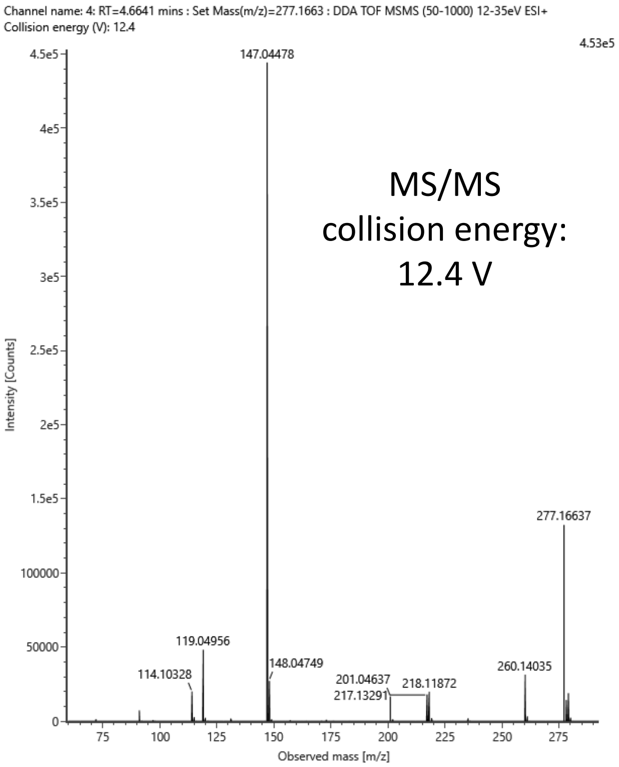

Compound name: feruloylagmatine
ESI-MS mode: positive, Unispray ion source
m/z (experimental): 307.17667

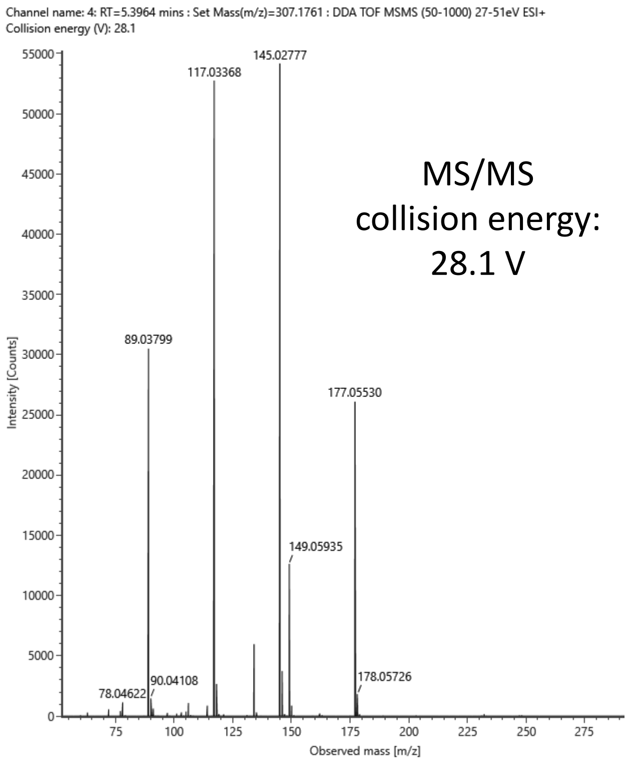

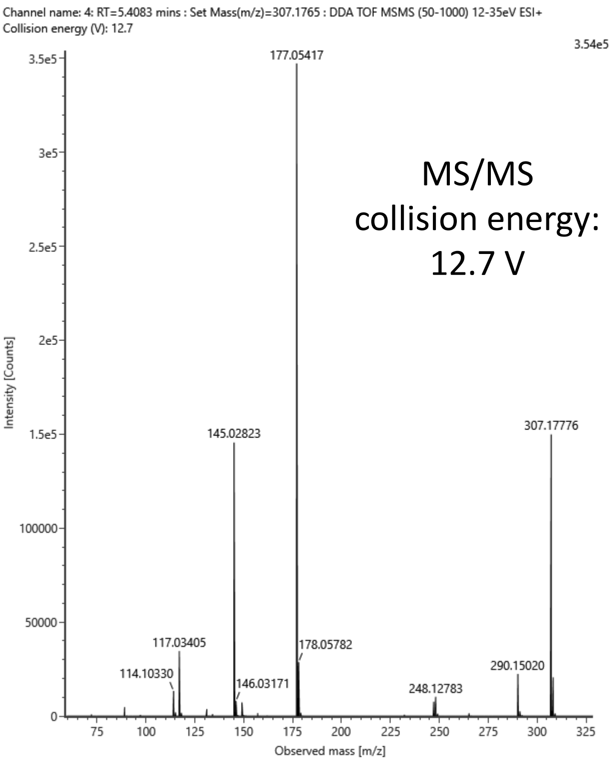

Compound name: lutonarin
ESI-MS mode: positive, Unispray ion source
m/z (experimental): 611.16099

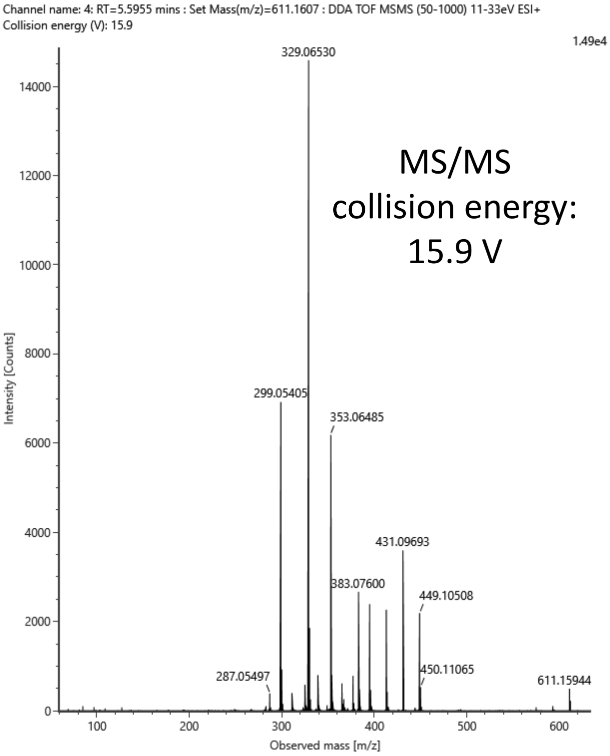

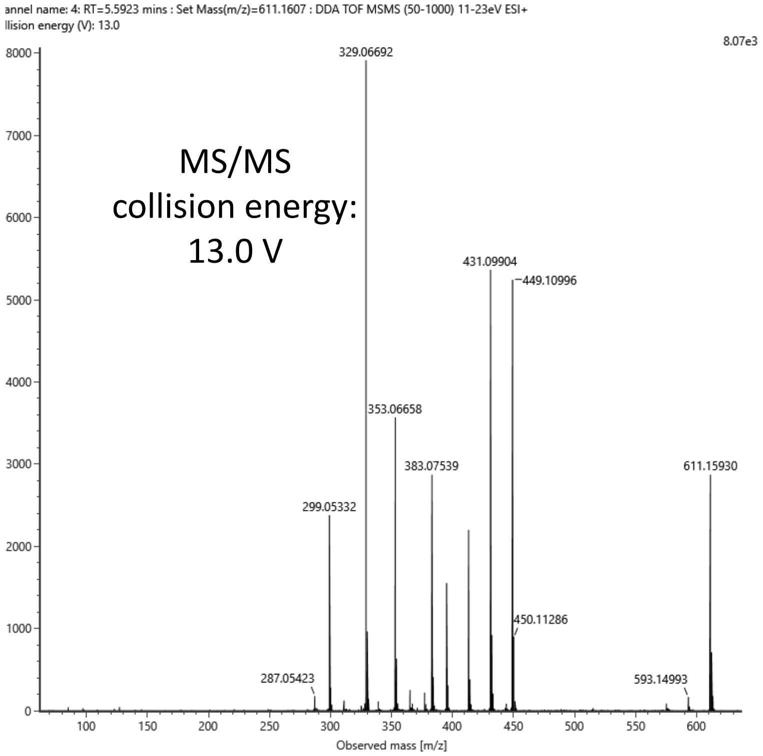

Compound name: lutonarin
ESI-MS mode: negative, Unispray ion source
m/z (experimental): 609.14620

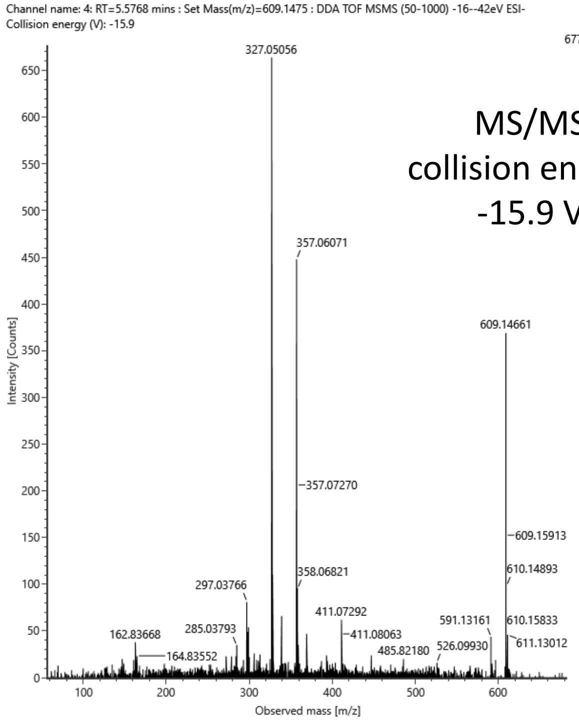

Compound name: vicenin-2
ESI-MS mode: positive, Unispray ion source
m/z (experimental): 595.16550

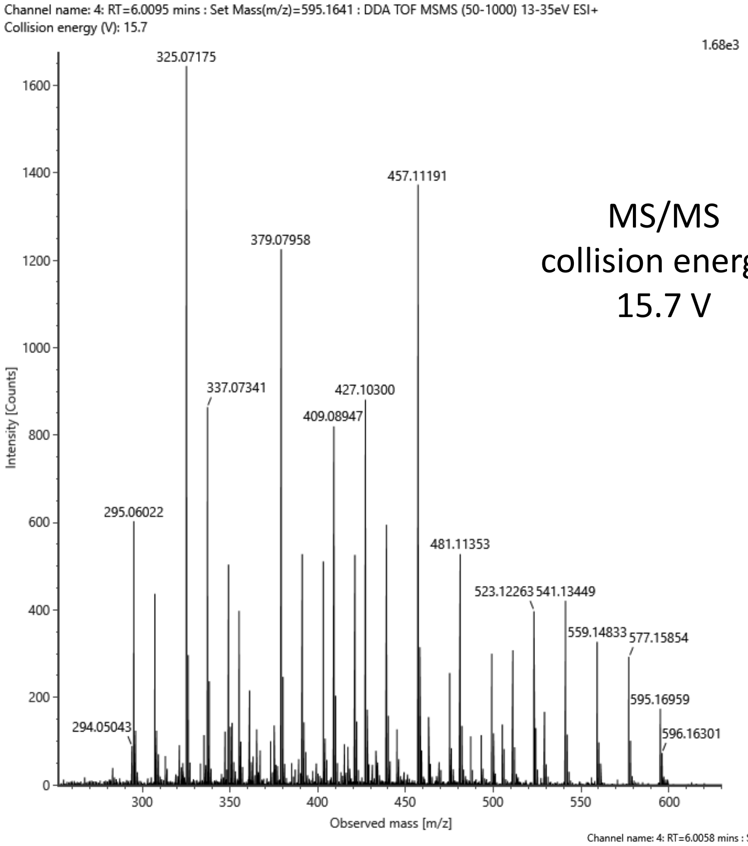

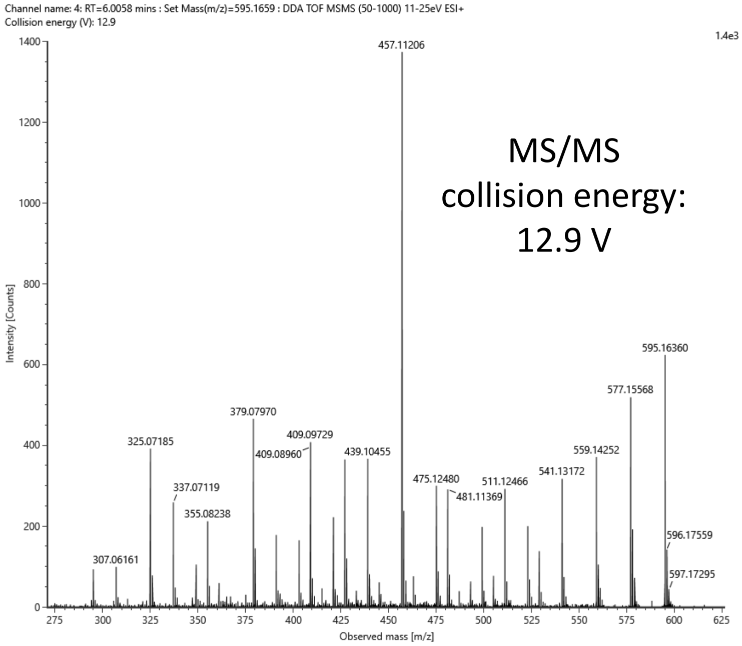

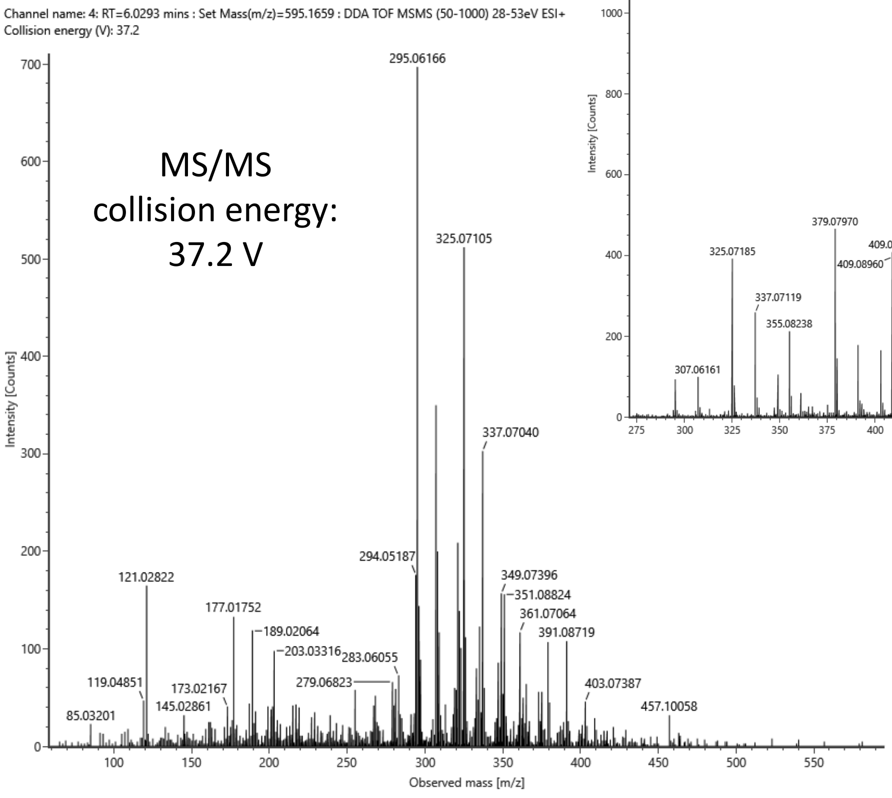

Compound name: vicenin-2
ESI-MS mode: negative, Unispray ion source
m/z (experimental): 593.15115

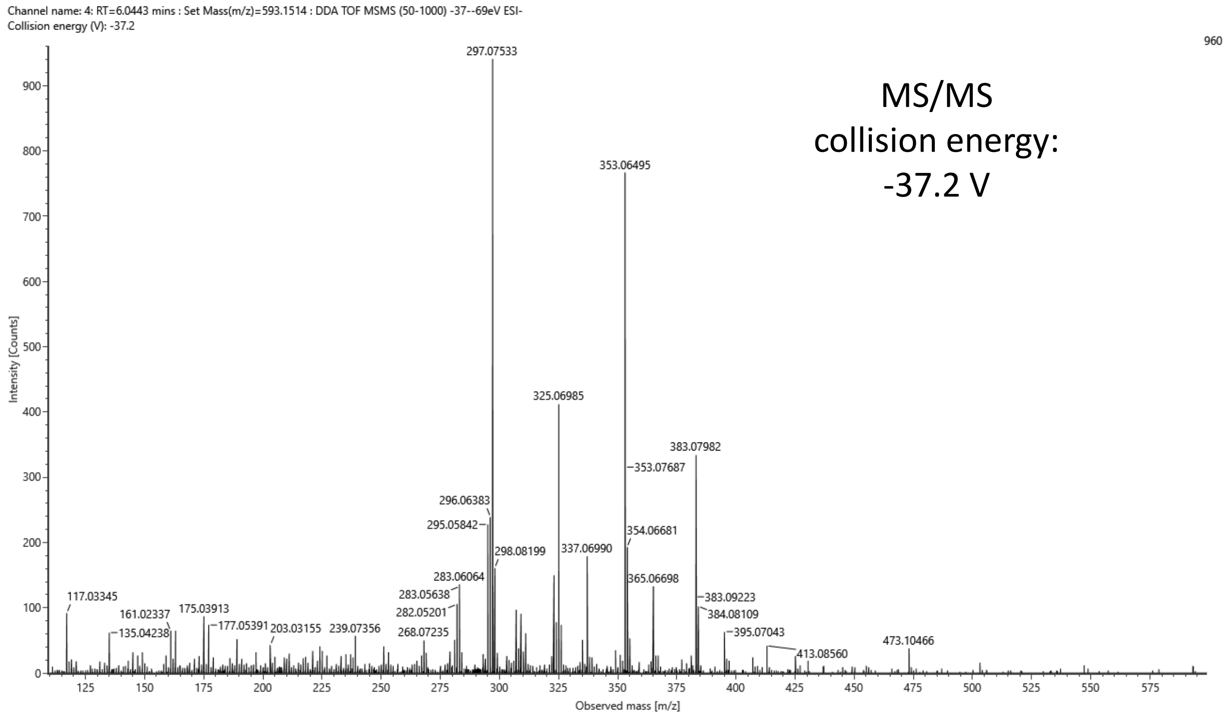

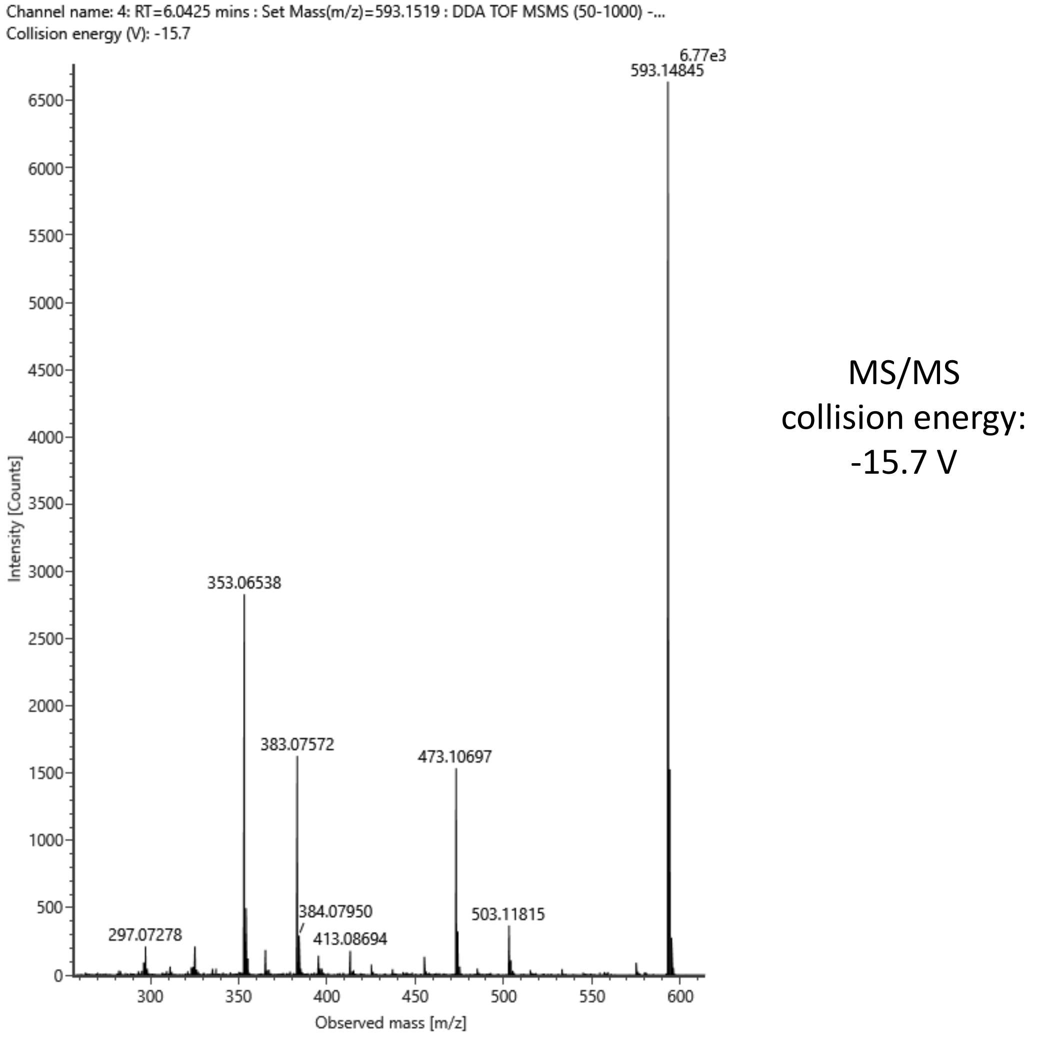

Compound name: lucenin-1/3
ESI-MS mode: positive, Unispray ion source
m/z (experimental): 581.15050

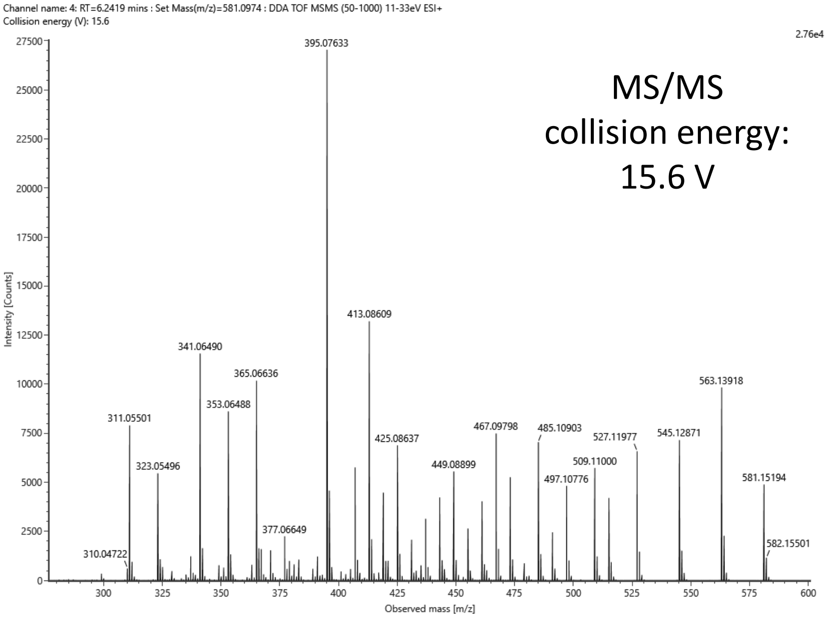

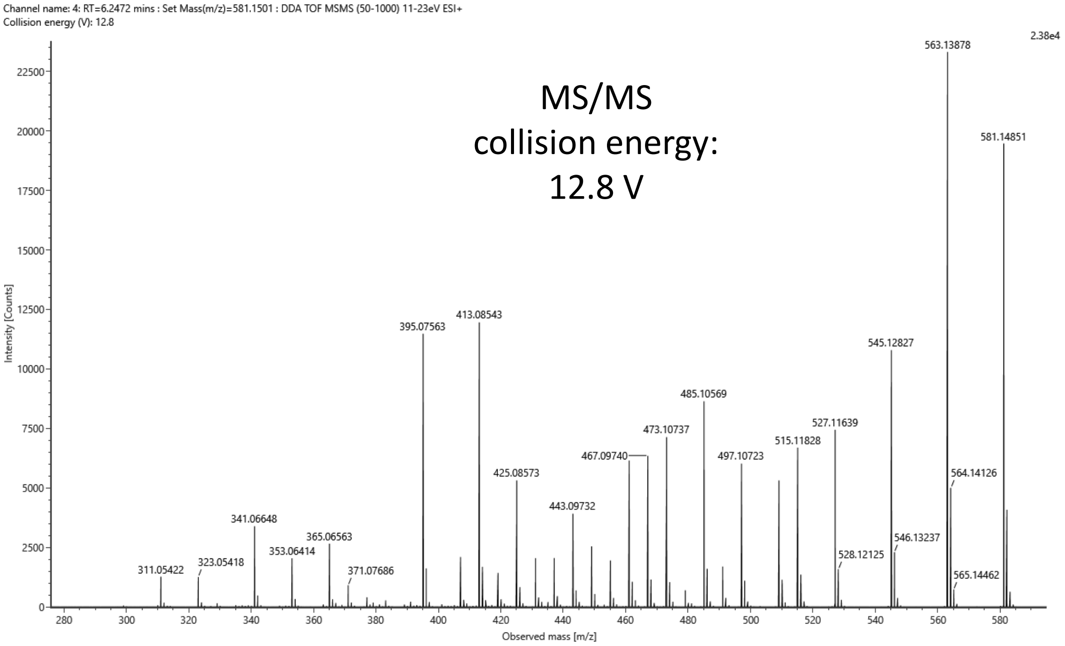

Compound name: lucenin-1/3
ESI-MS mode: negative, Unispray ion source
m/z (experimental): 579.13497

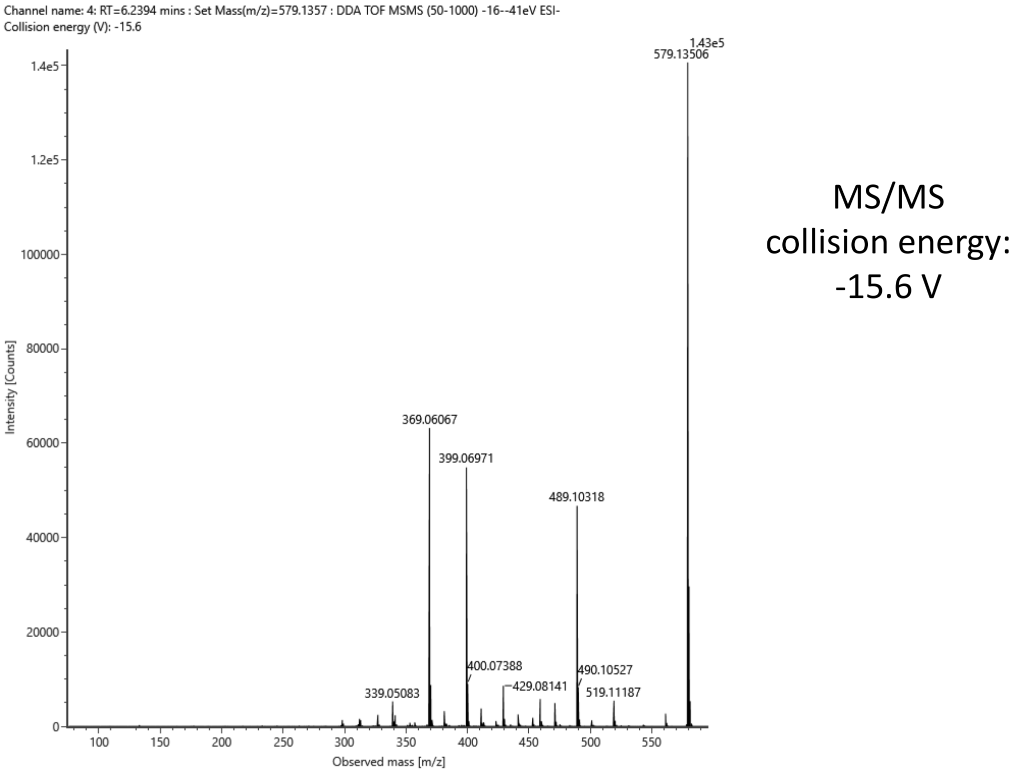

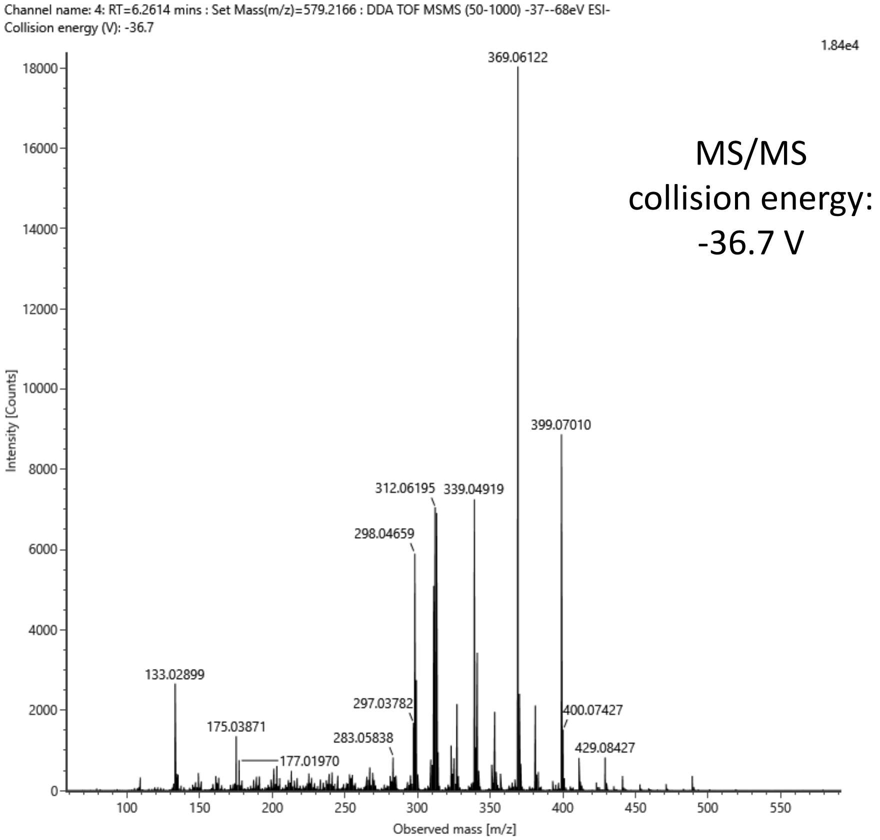

Compound name: isoorientin-2"-O-glucoside
ESI-MS mode: positive, Unispray ion source
m/z (experimental): 611.16080

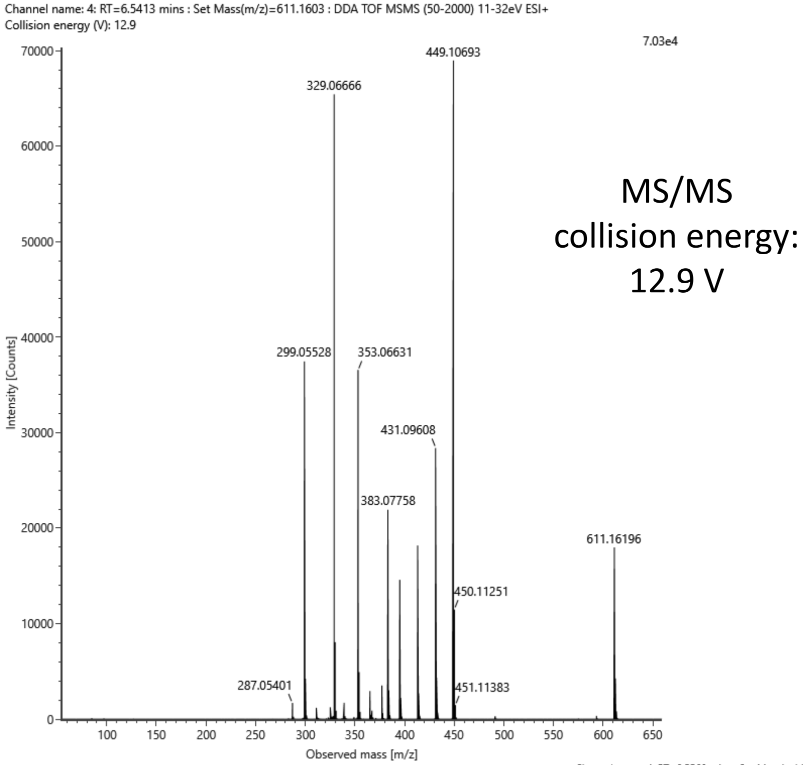

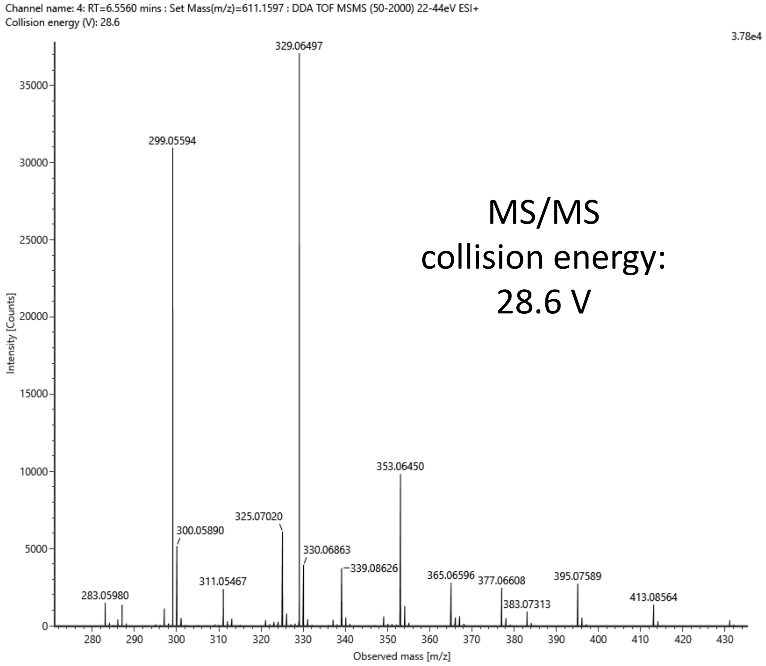

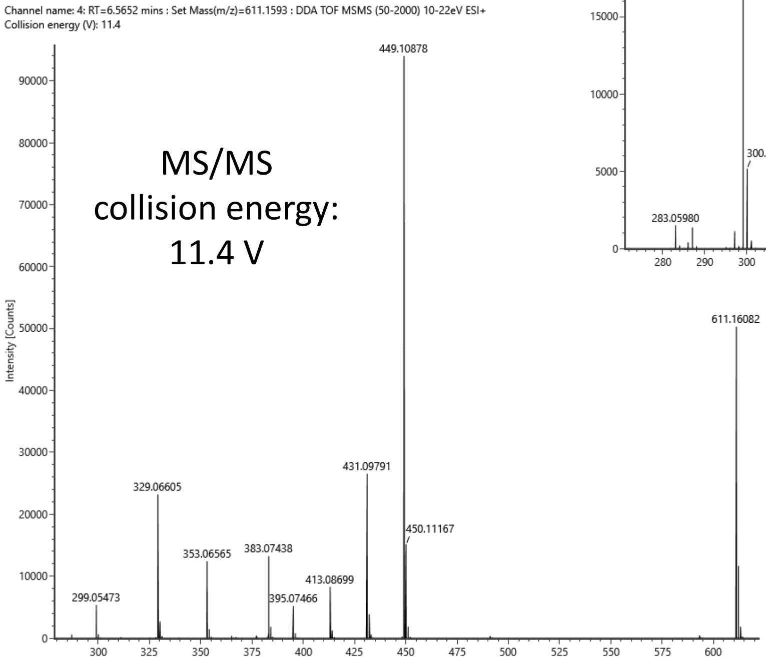

Compound name: isoorientin-2"-O-glucoside
ESI-MS mode: negative, Unispray ion source
m/z (experimental): 609.14659

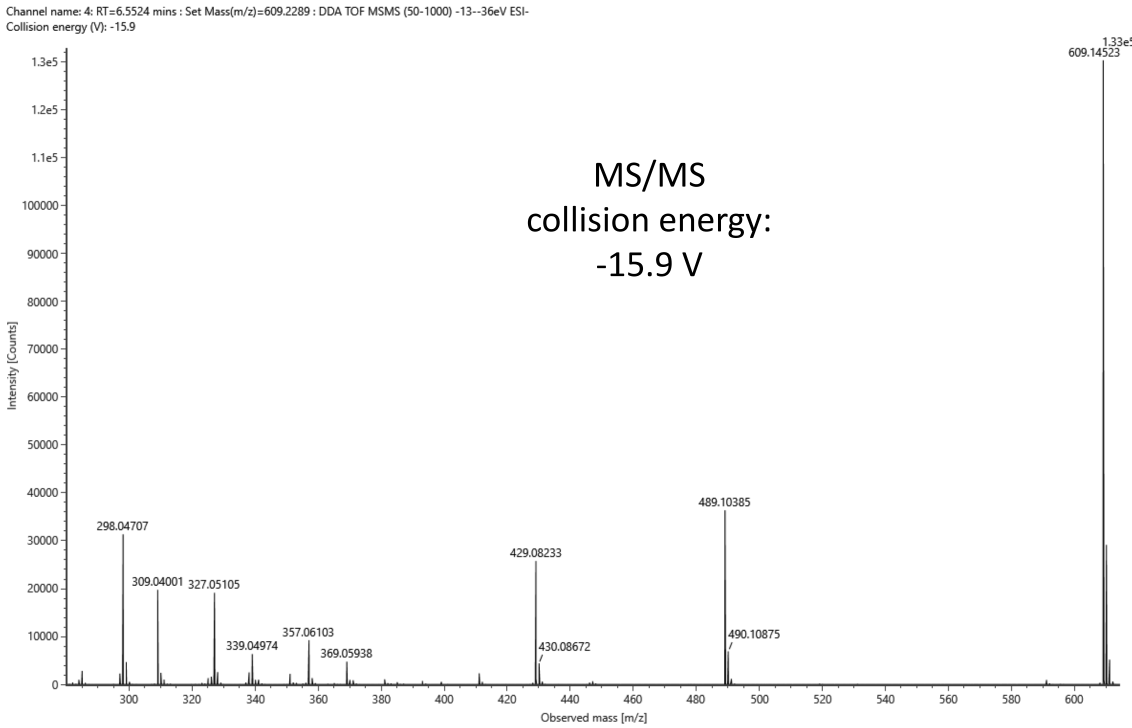

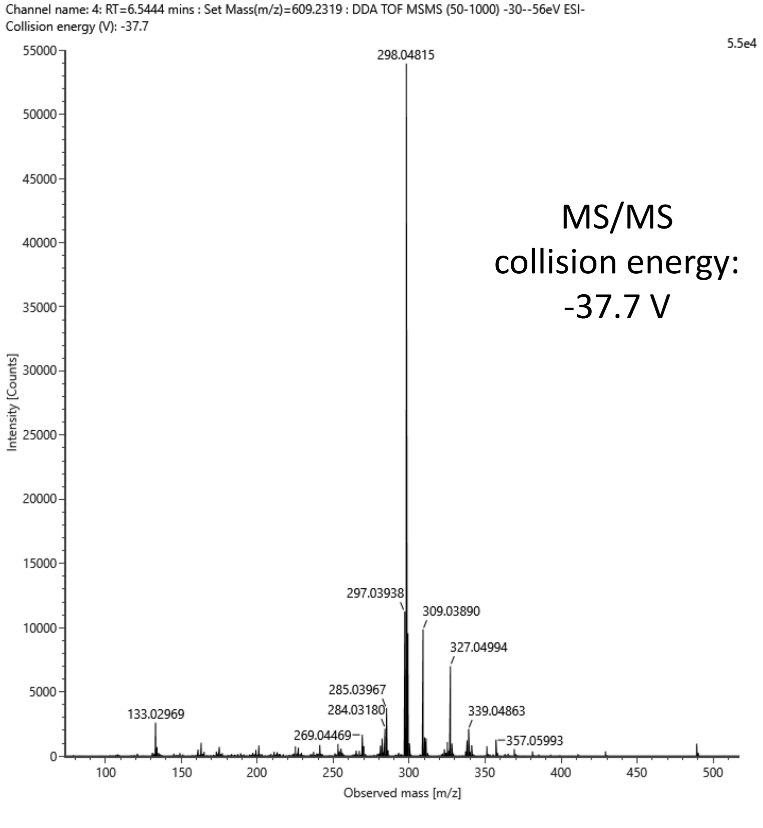

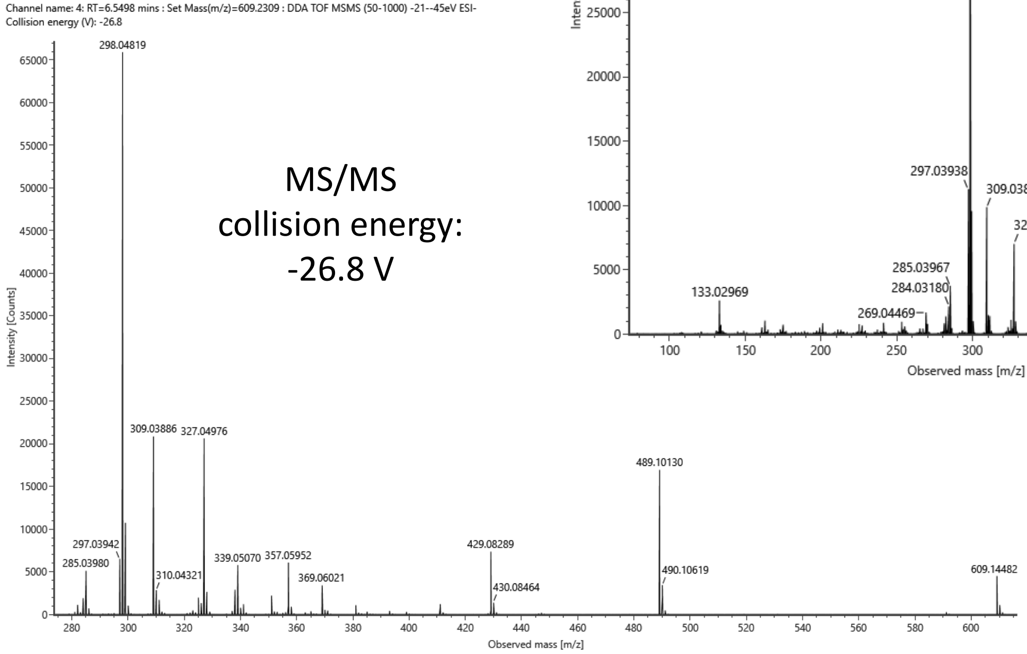

Compound name: saponarin
ESI-MS mode: positive, Unispray ion source
m/z (experimental): 595.16581

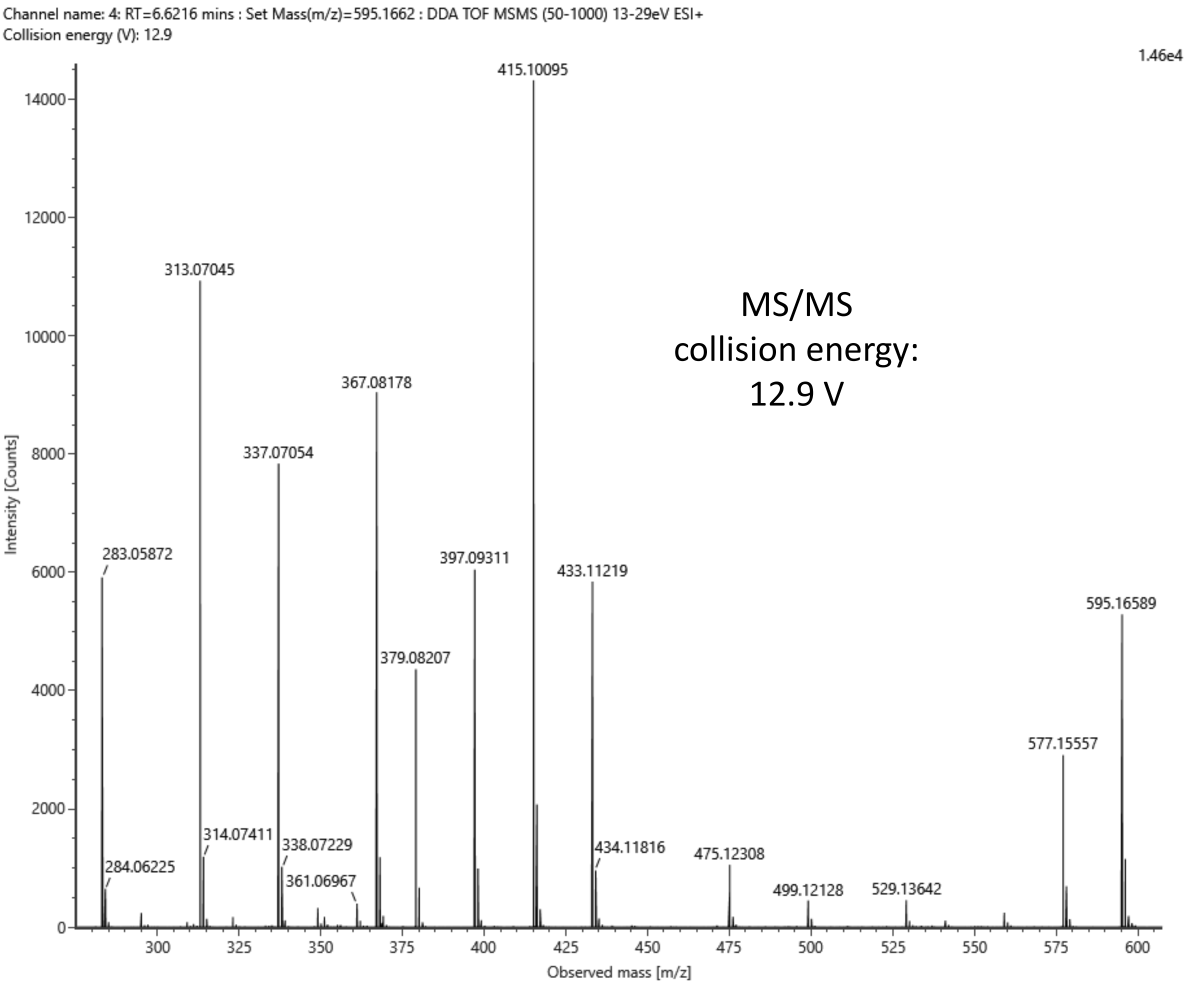

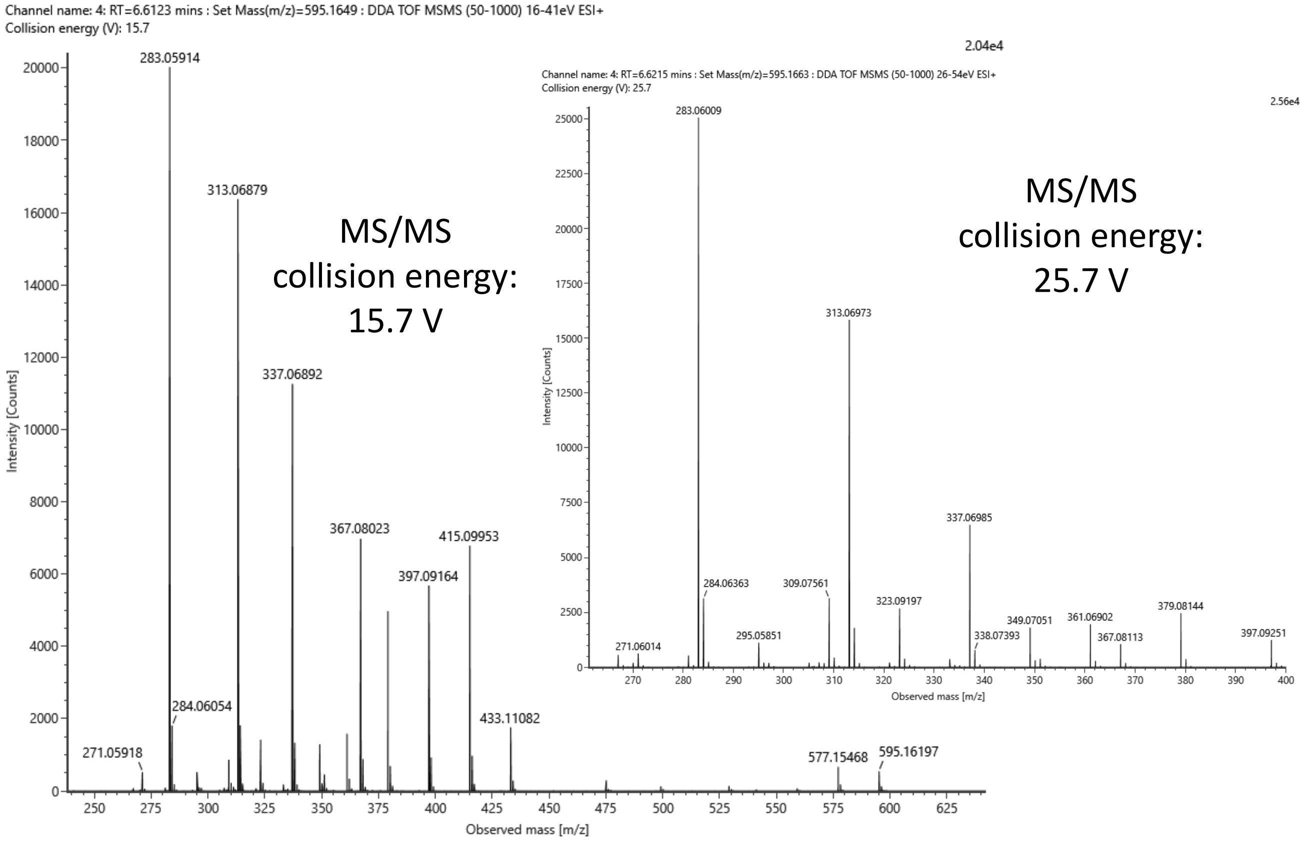

Compound name: saponarin
ESI-MS mode: negative, Unispray ion source
m/z (experimental): 593.15088

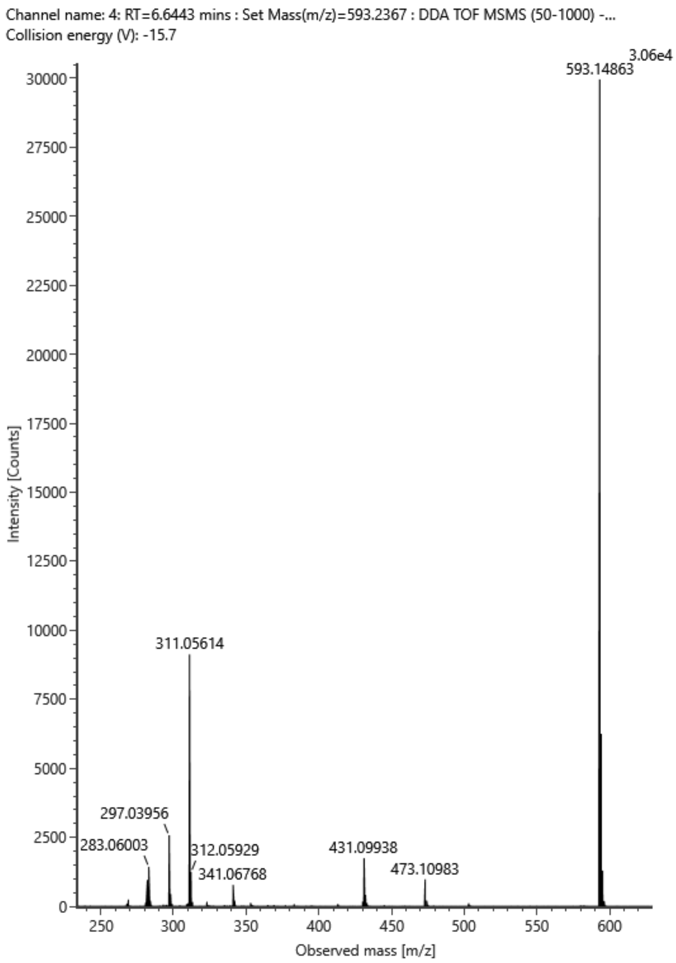

MS/MS
collision energy:
-15.7 V

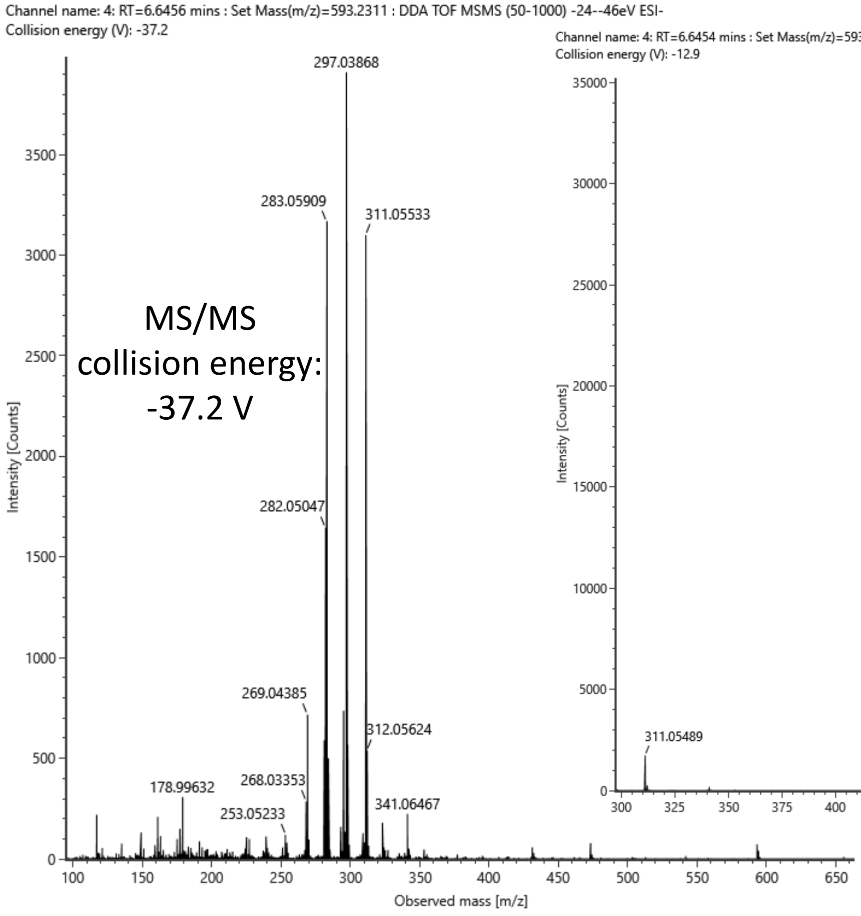

MS/MS
collision energy:
-37.2 V

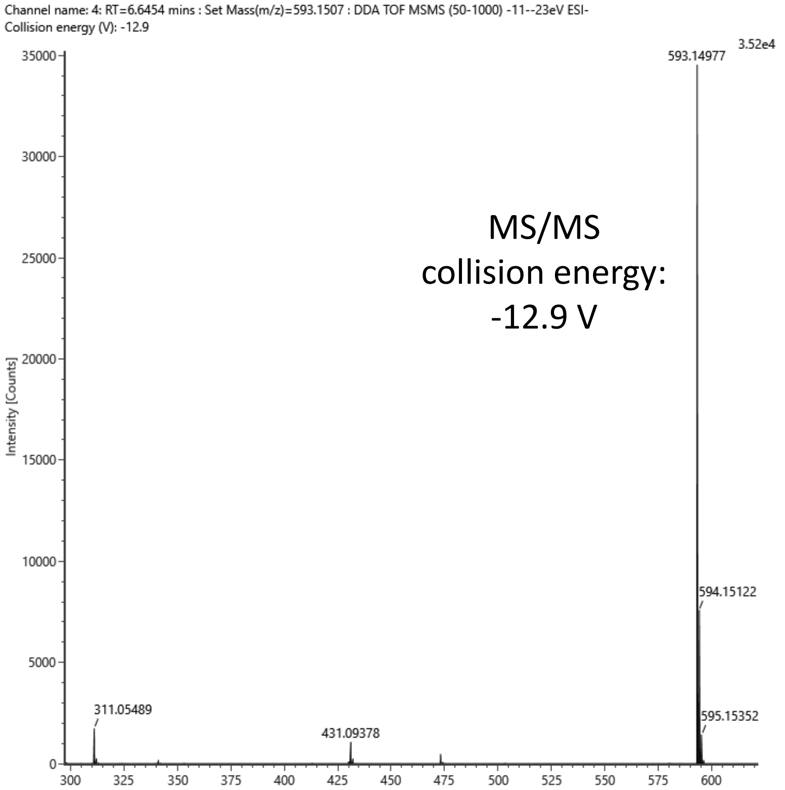

MS/MS
collision energy:
-12.9 V

Compound name: isoorientin
ESI-MS mode: positive, Unispray ion source
m/z (experimental): 449.10758

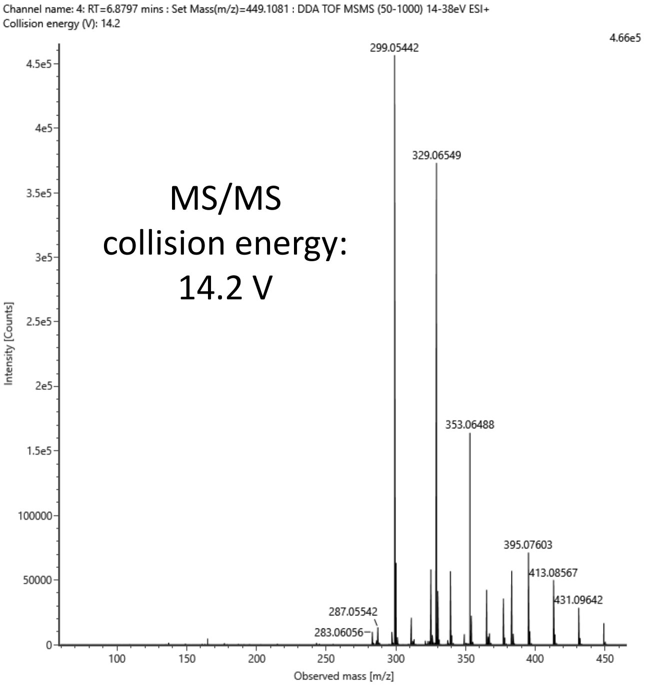

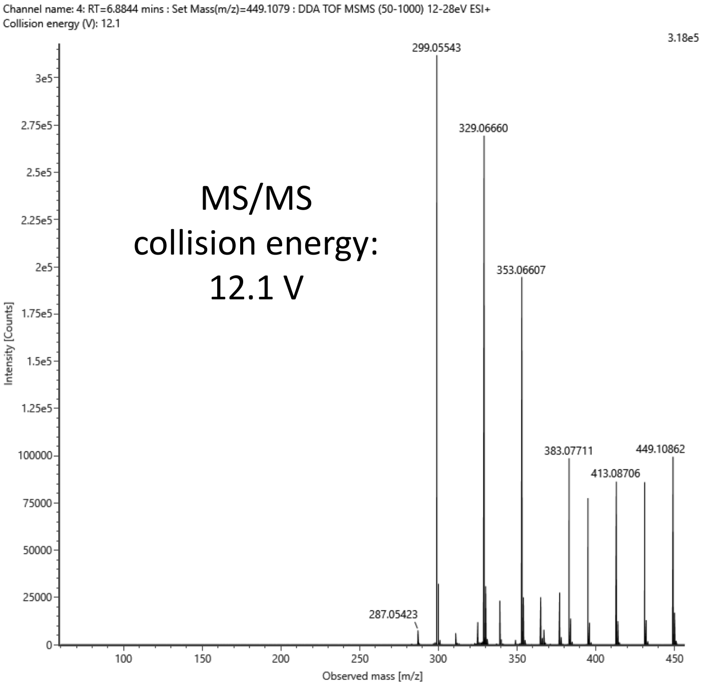

Compound name: isoorientin
ESI-MS mode: negative, Unispray ion source
m/z (experimental): 447.09354

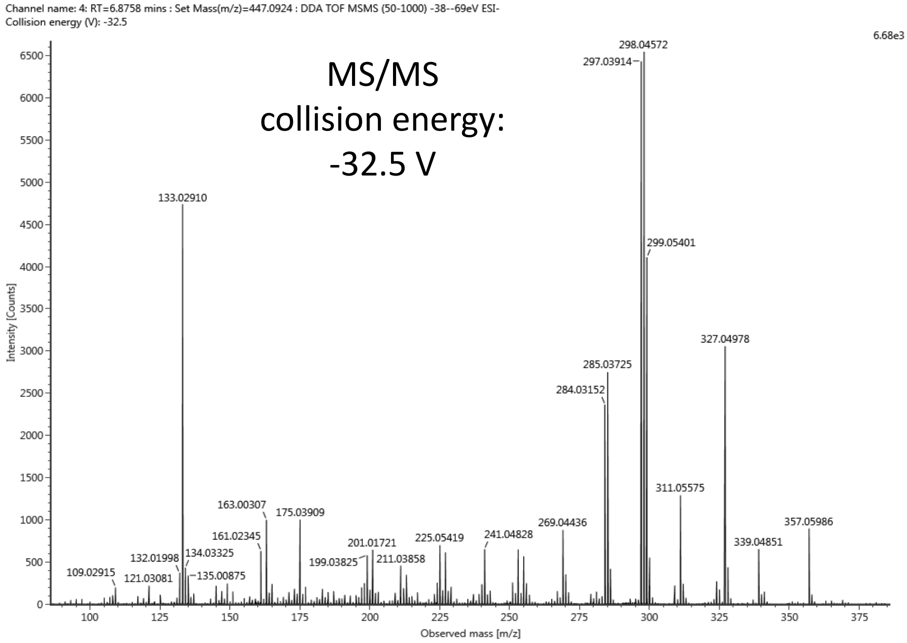

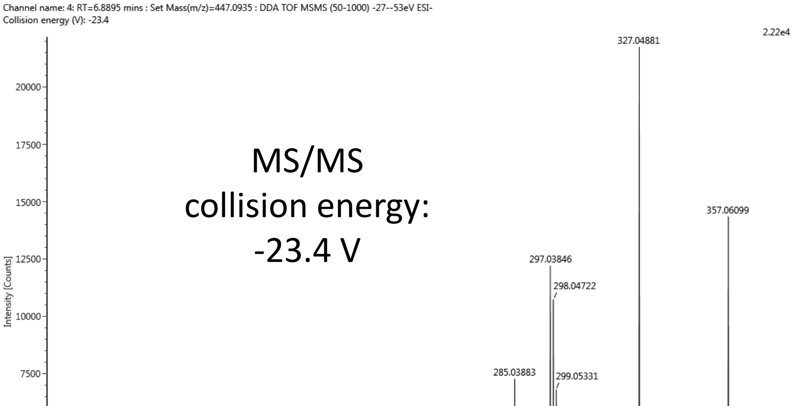

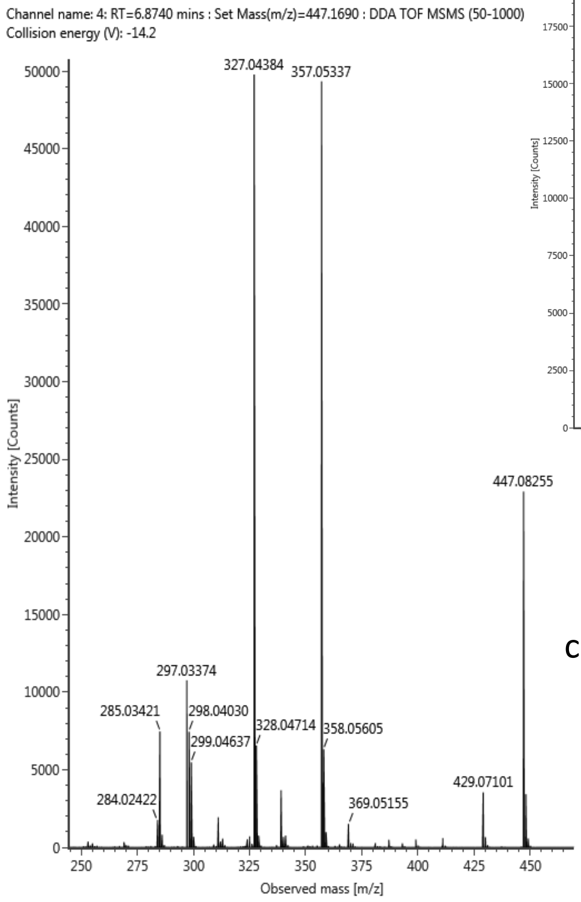

MS/MS
collision energy:
-14.2 V

Compound name: isoschaftoside
ESI-MS mode: positive, Unispray ion source
m/z (experimental): 565.15527

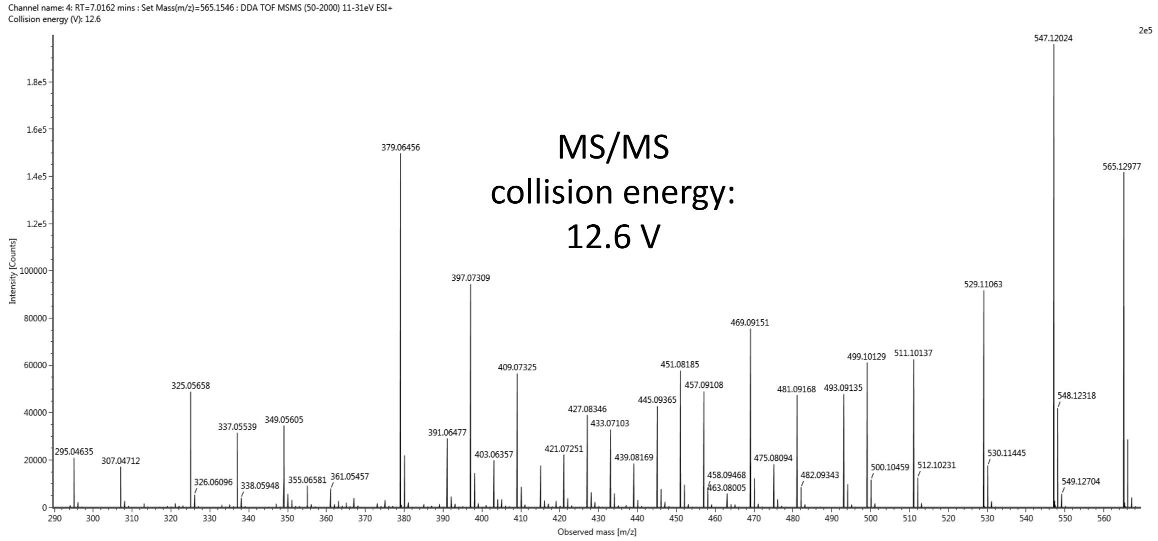

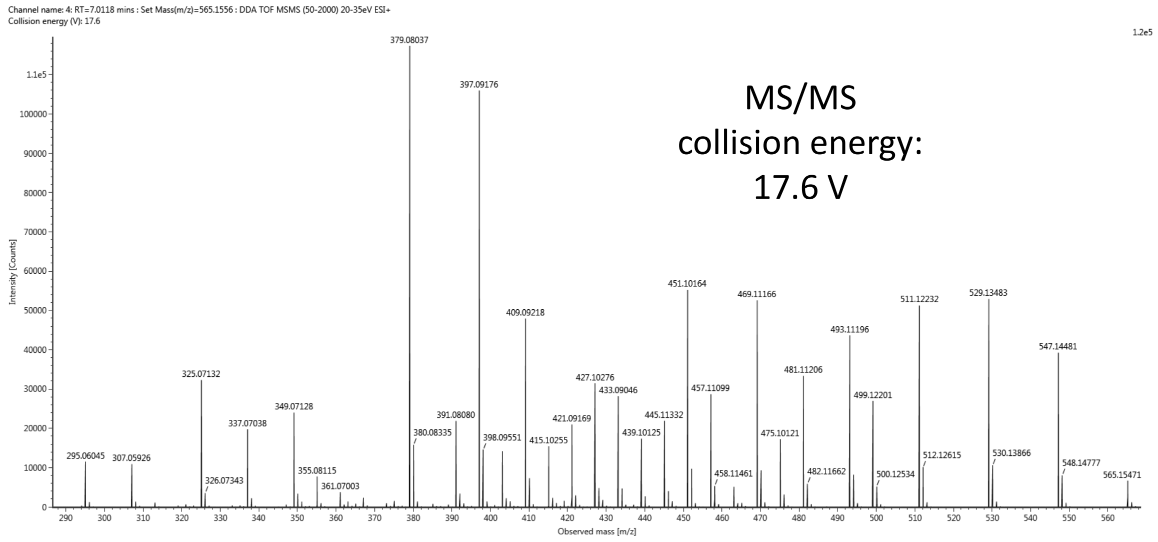

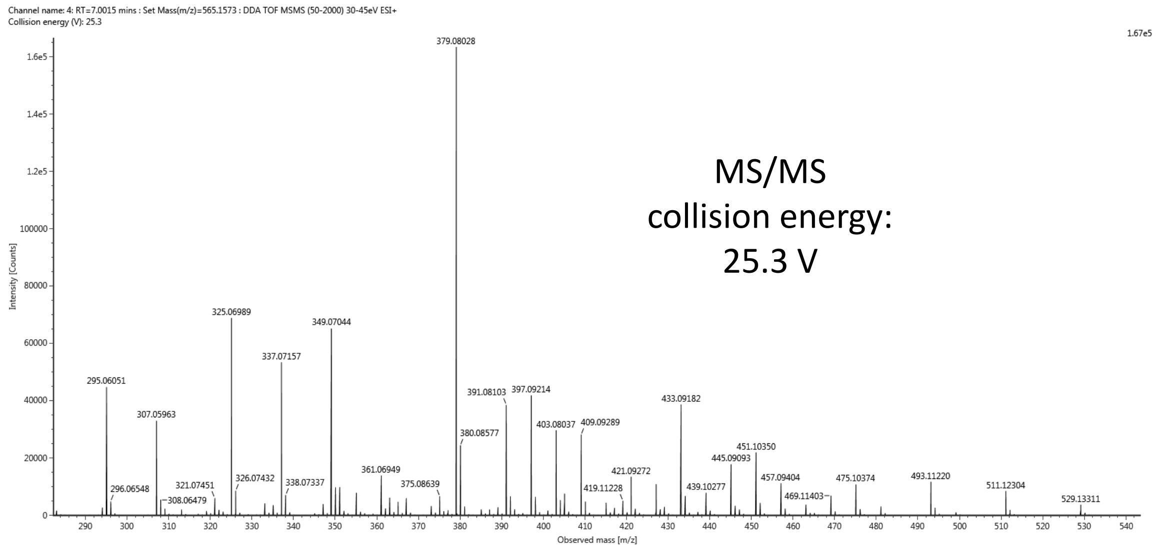

Compound name: isoschaftoside
ESI-MS mode: negative, Unispray ion source
m/z (experimental): 563.14050

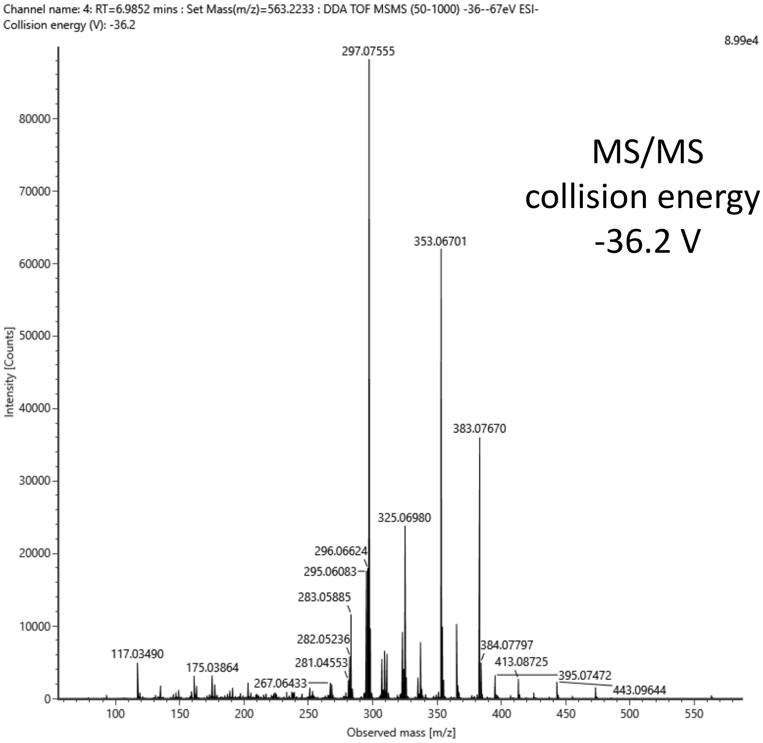

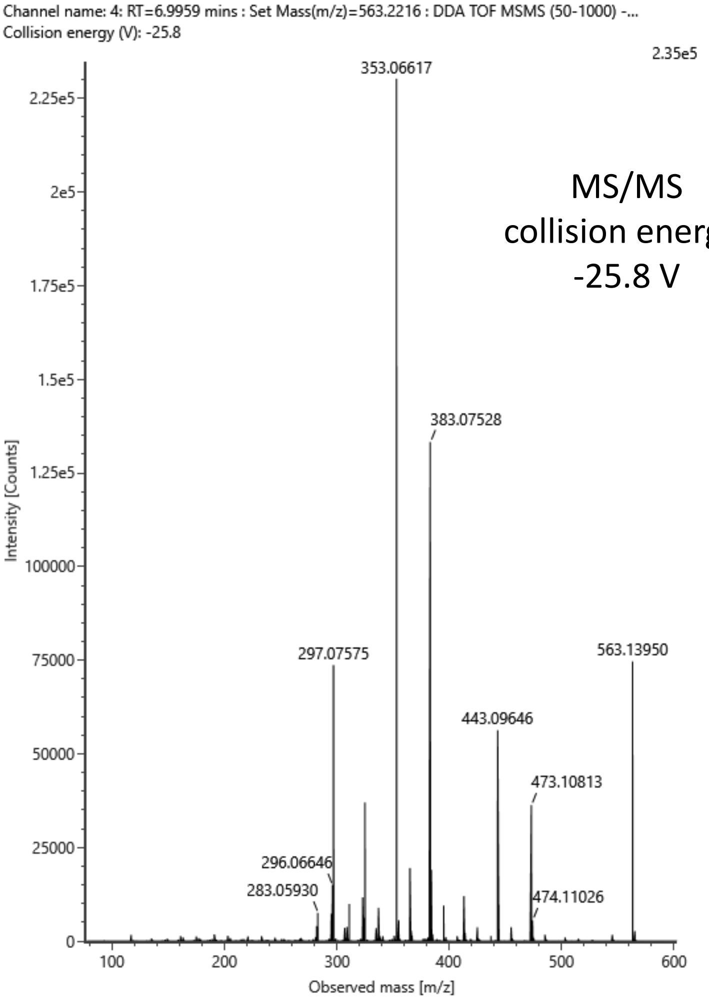

Compound name: isoorientin 6''-rhamnoside
ESI-MS mode: positive, Unispray ion source
m/z (experimental): 595.16599

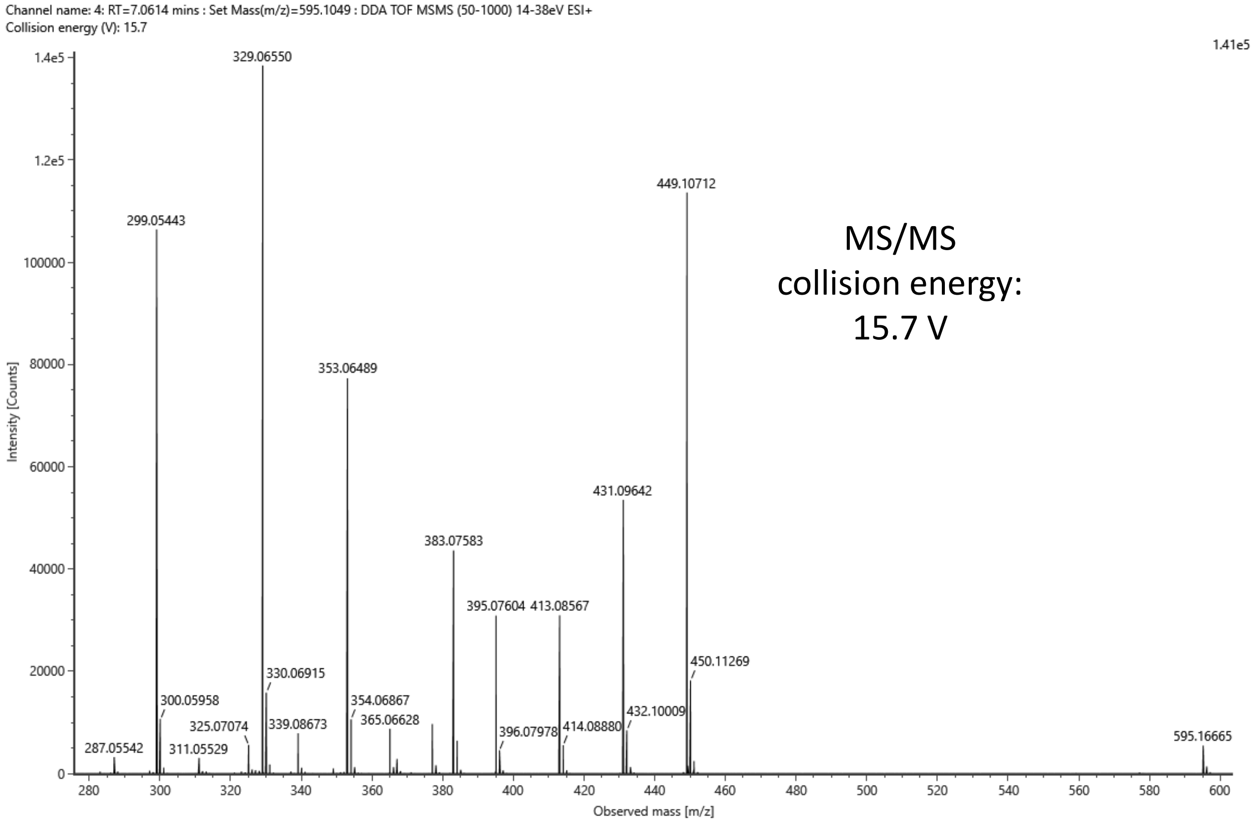

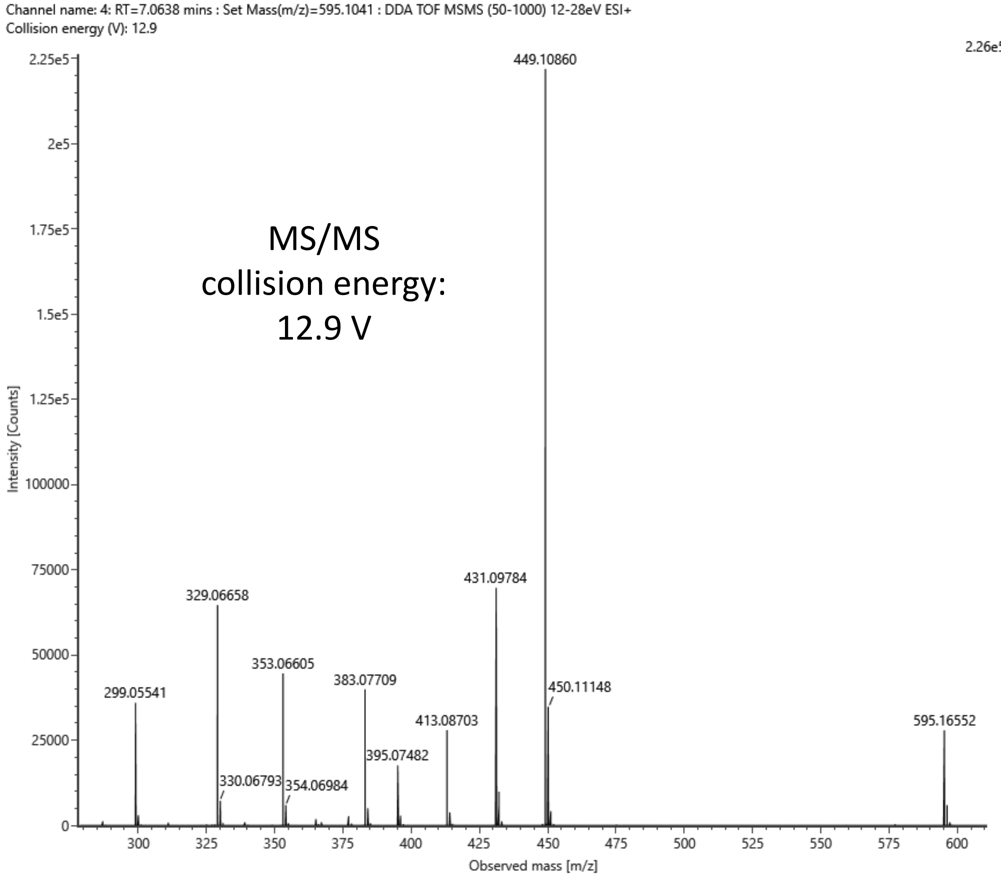

Compound name: isoorientin 6''-rhamnoside
ESI-MS mode: negative, Unispray ion source
m/z (experimental): 593.15087

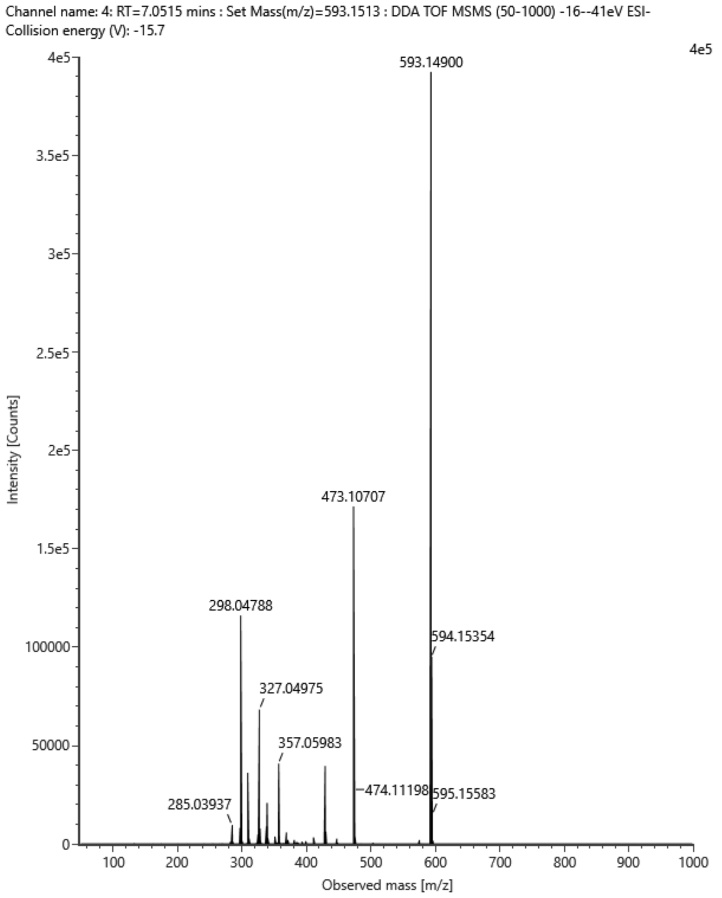

MS/MS
collision energy:
-15.7 V

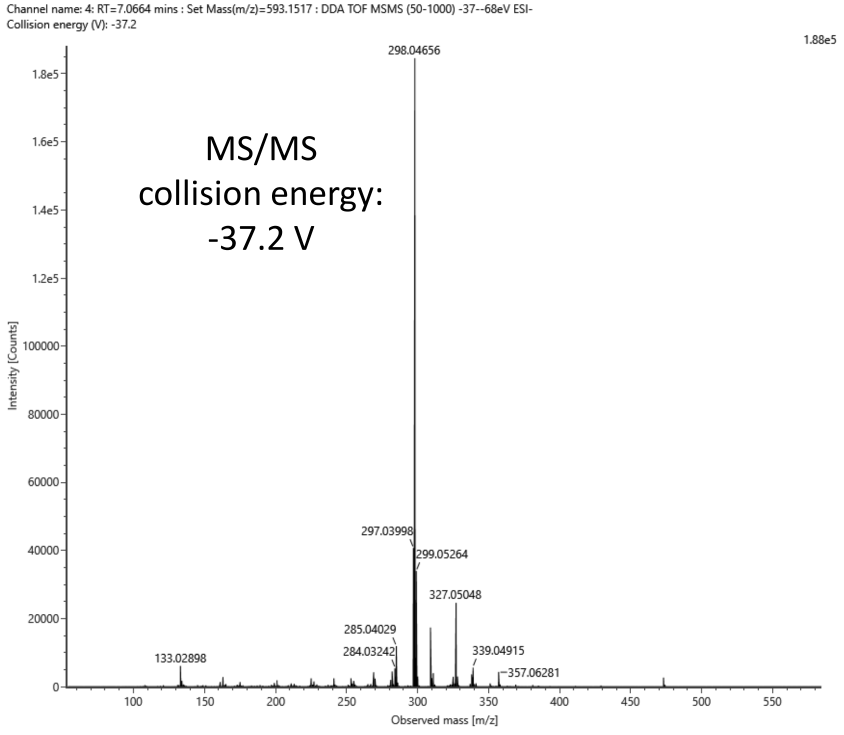

MS/MS
collision energy:
-37.2 V

Compound name: isoscoparin-7-O-glucoside
ESI-MS mode: positive, Unispray ion source
m/z (experimental): 625.17585

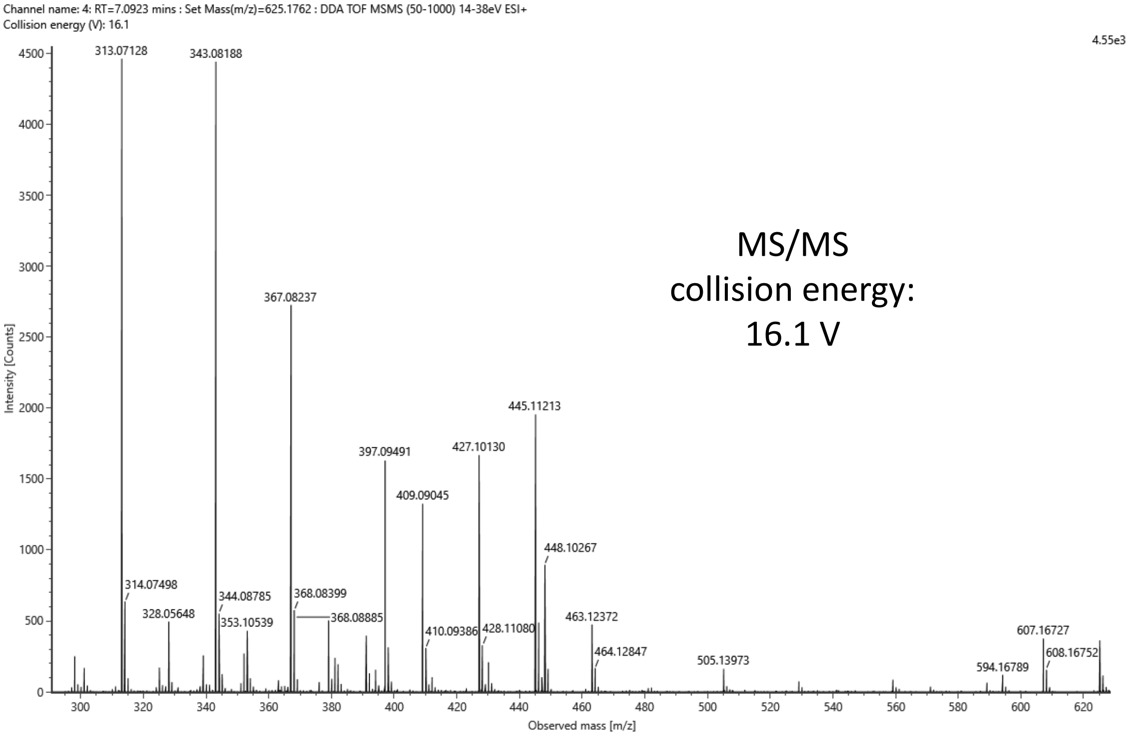

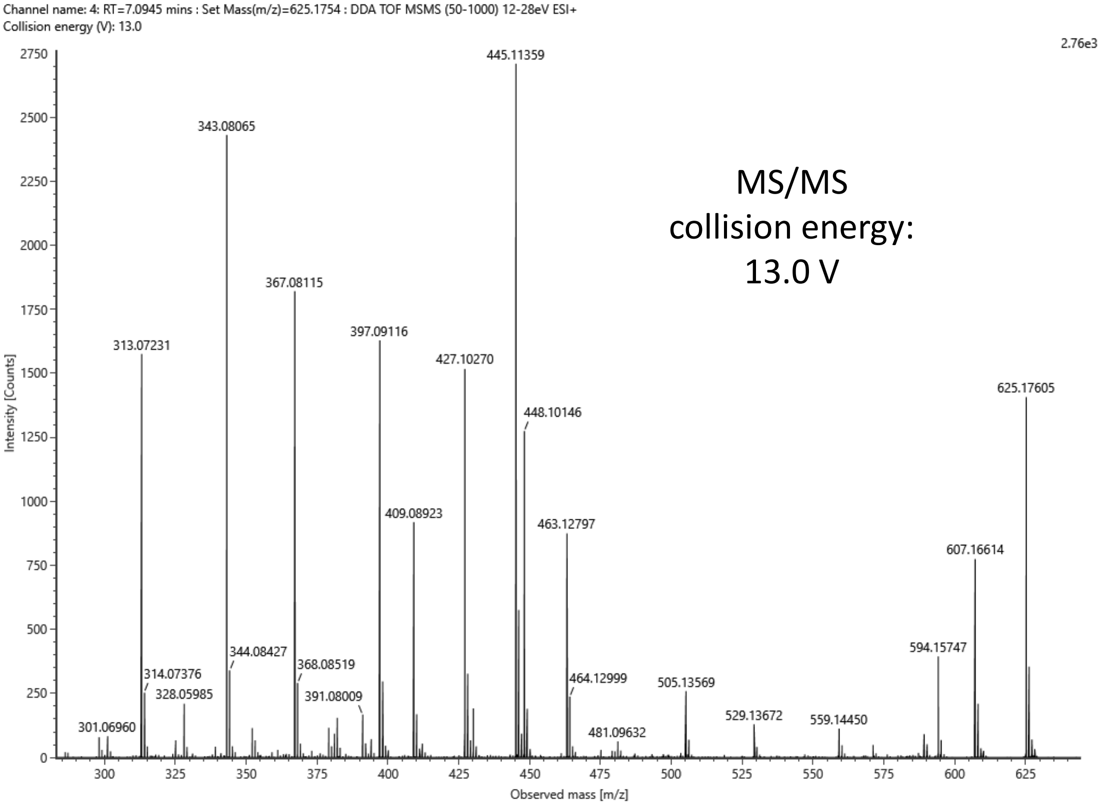

Compound name: isoscoparin-7-O-glucoside
ESI-MS mode: negative, Unispray ion source
m/z (experimental): 623.16144

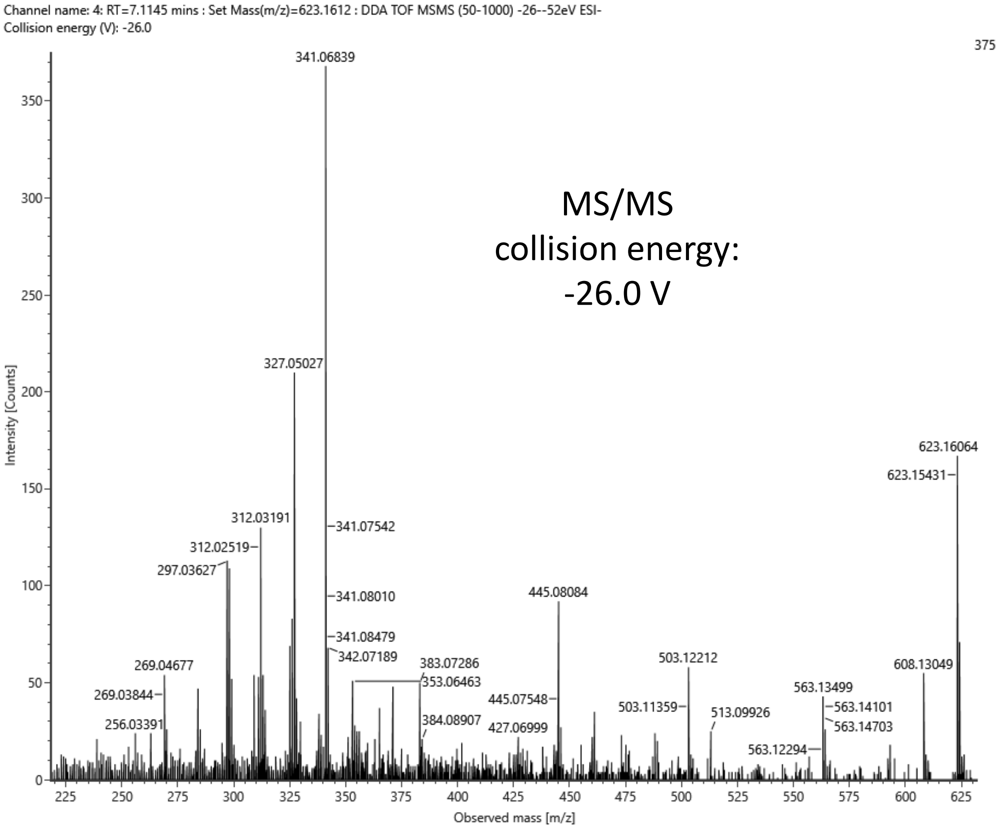

Compound name: isovitexin-2"-O-glucoside
ESI-MS mode: positive, Unispray ion source
m/z (experimental): 595.16536

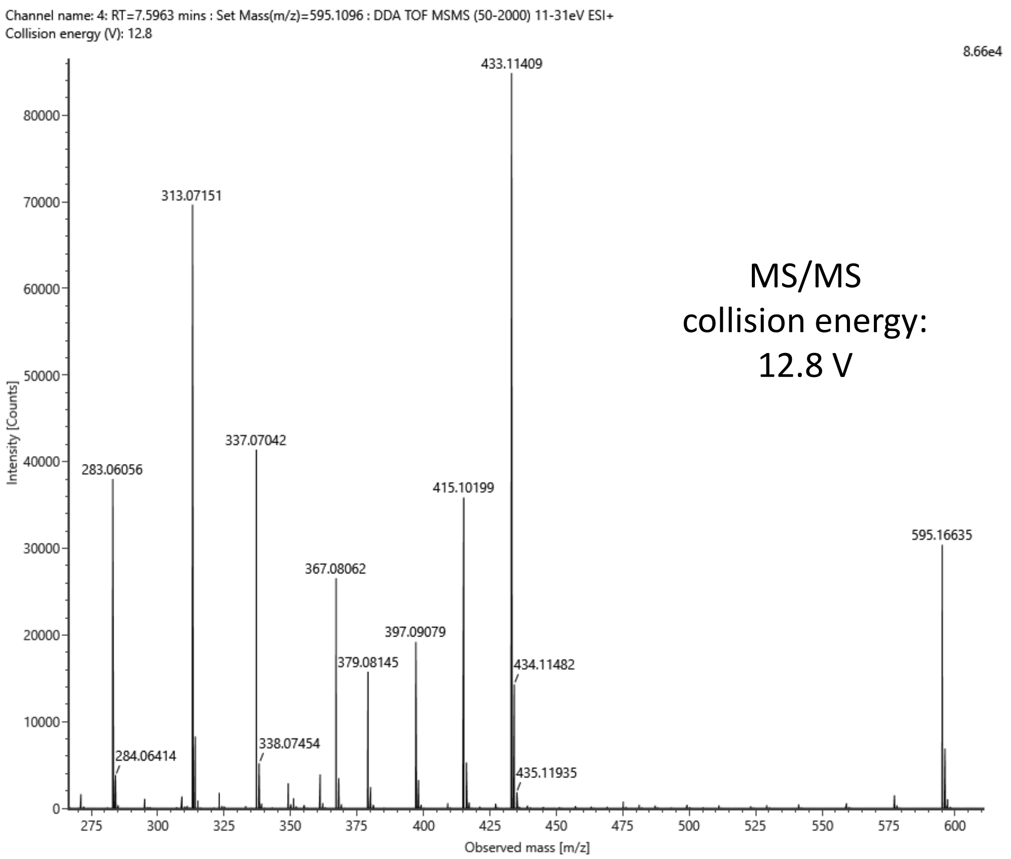

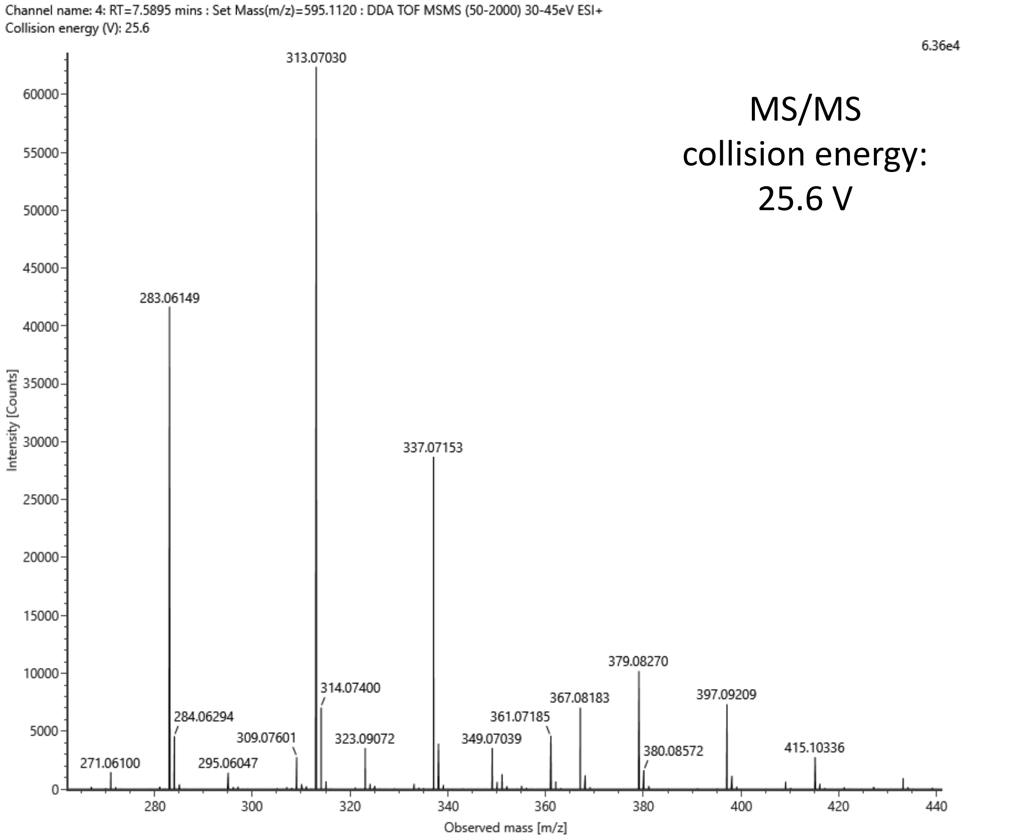

Compound name: isovitexin-2″-O-glucoside

ESI-MS mode: negative, Unispray ion source

m/z (experimental): 593.15155

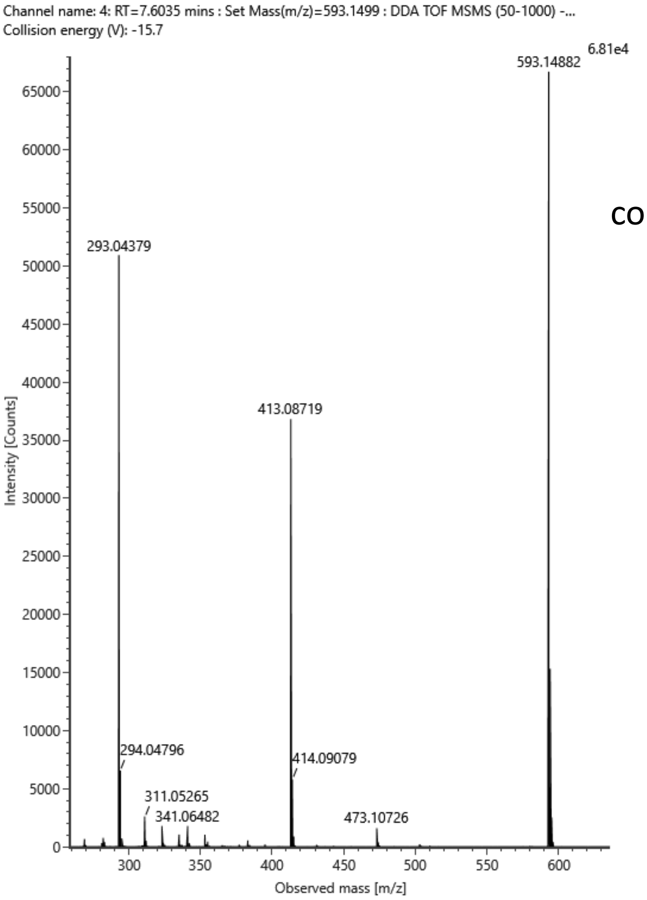

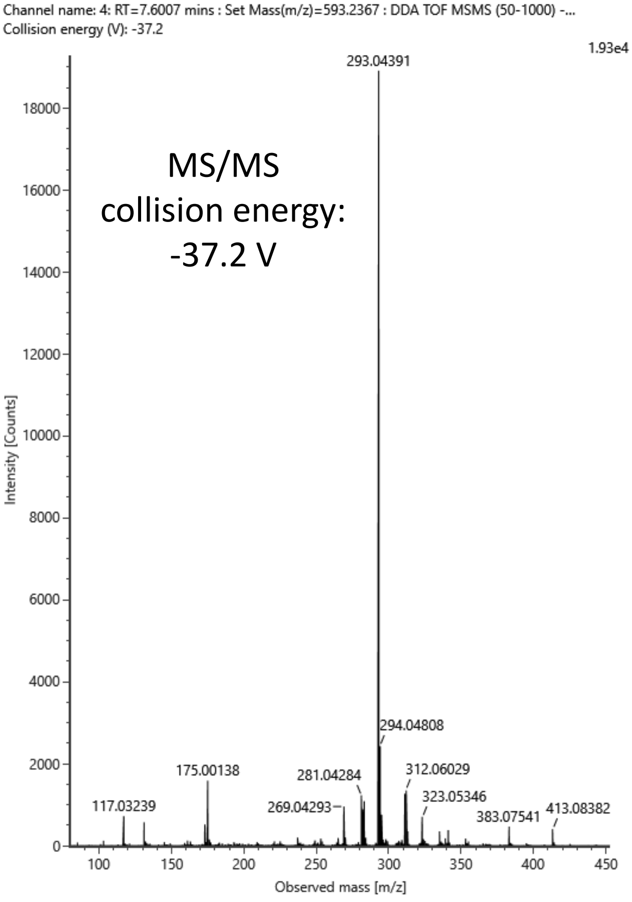

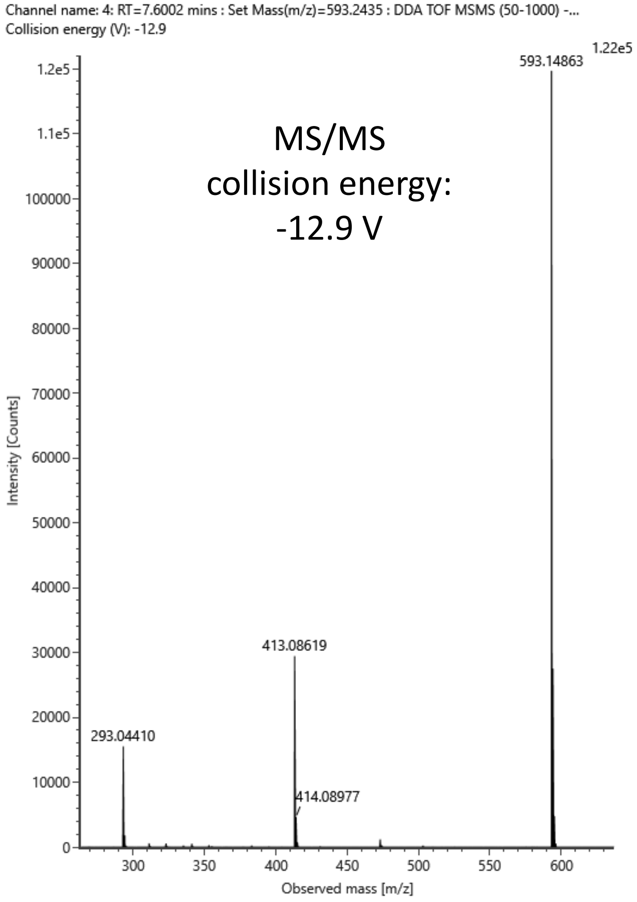

Compound name: isovitexin
ESI-MS mode: positive, Unispray ion source
m/z (experimental): 433.11284

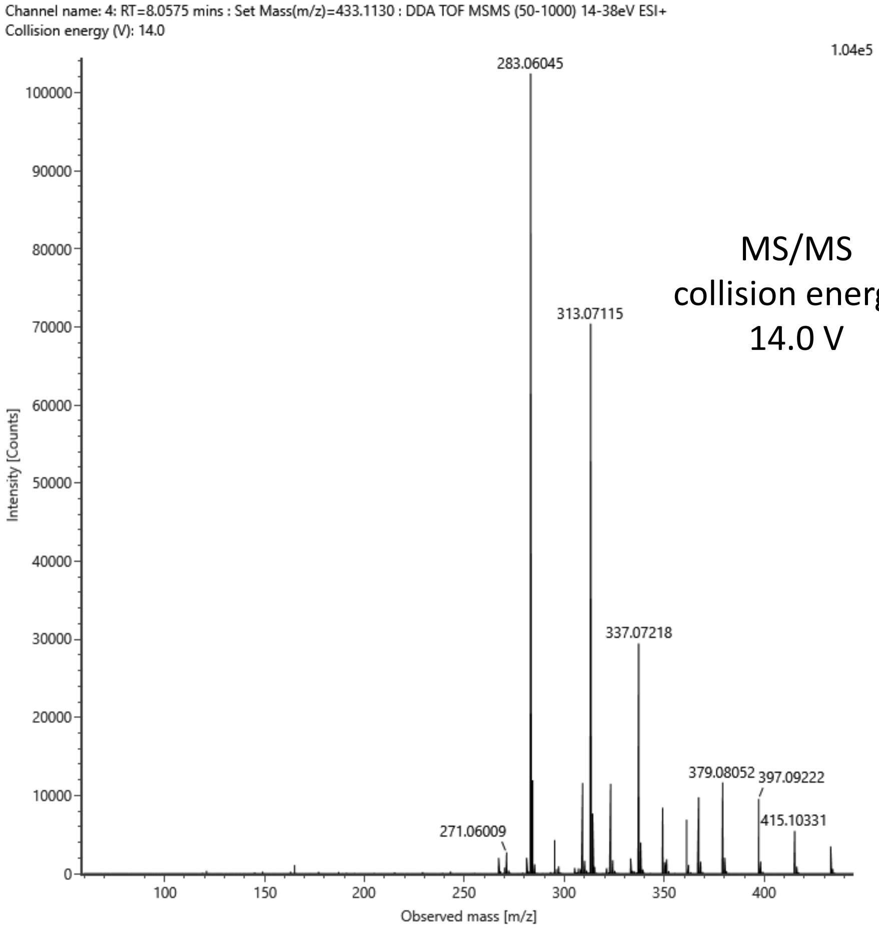

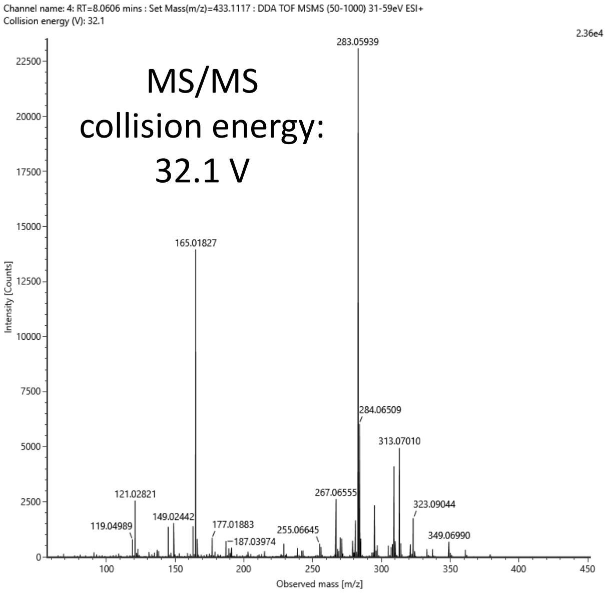

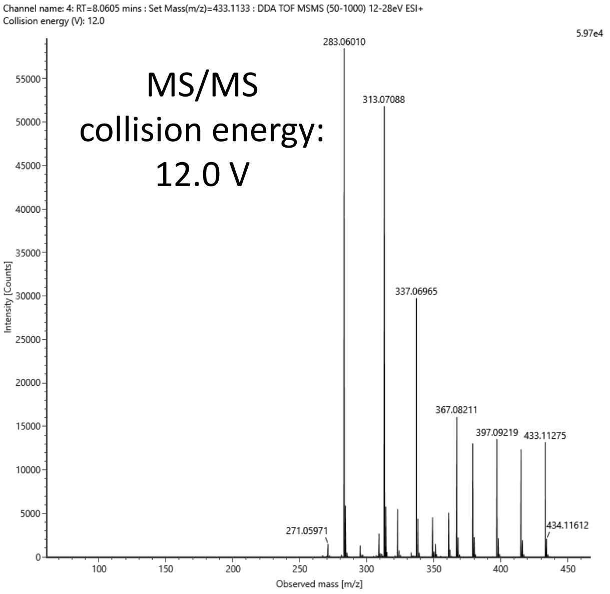

Compound name: isovitexin
ESI-MS mode: negative, Unispray ion source
m/z (experimental): 431.09853

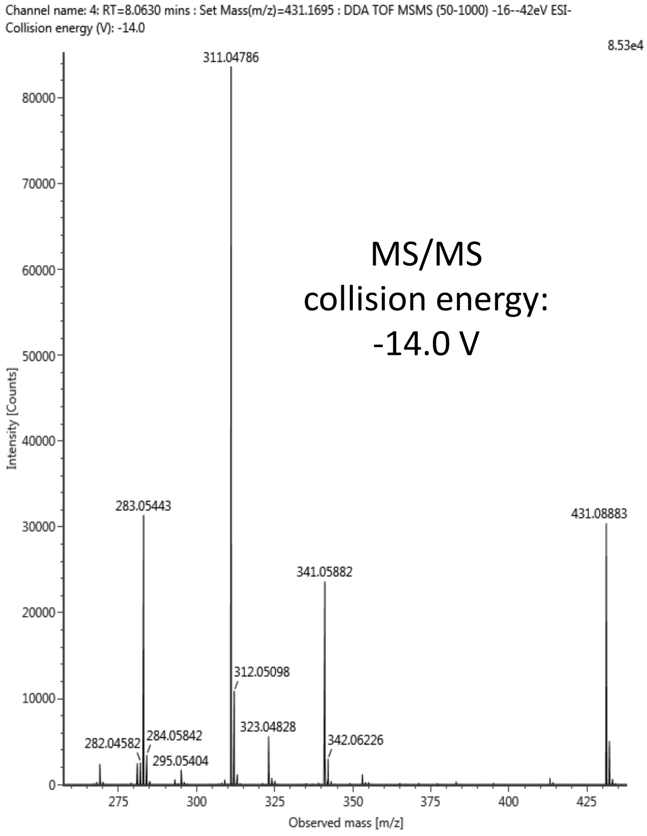

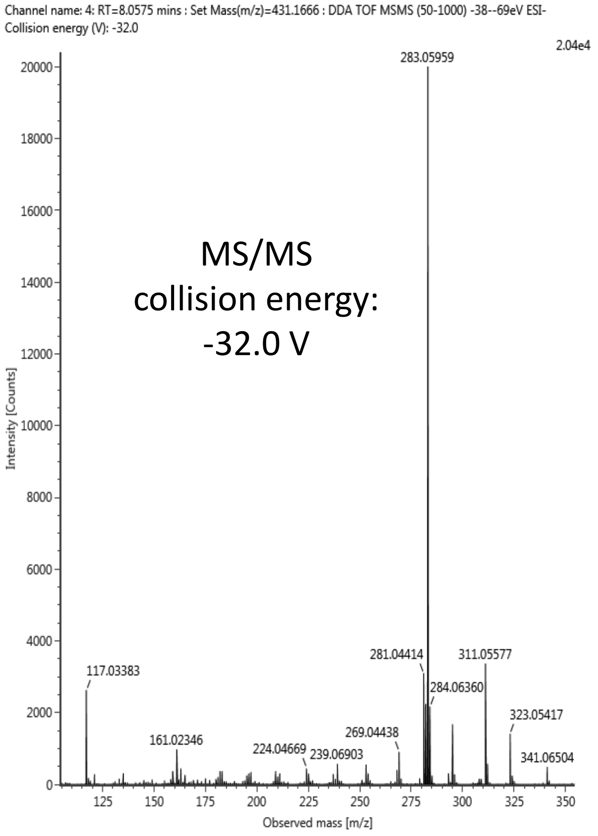

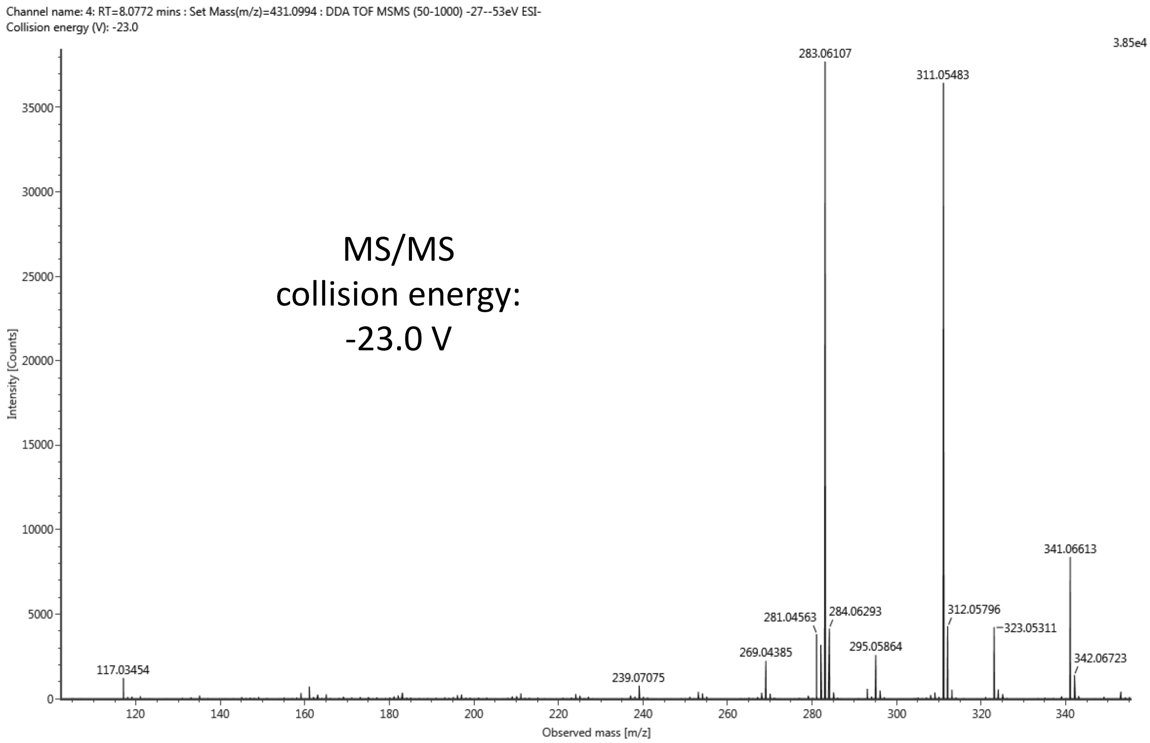

Compound name: isoscoparin-2"-O-glucoside
ESI-MS mode: positive, Unispray ion source
m/z (experimental): 625.17628

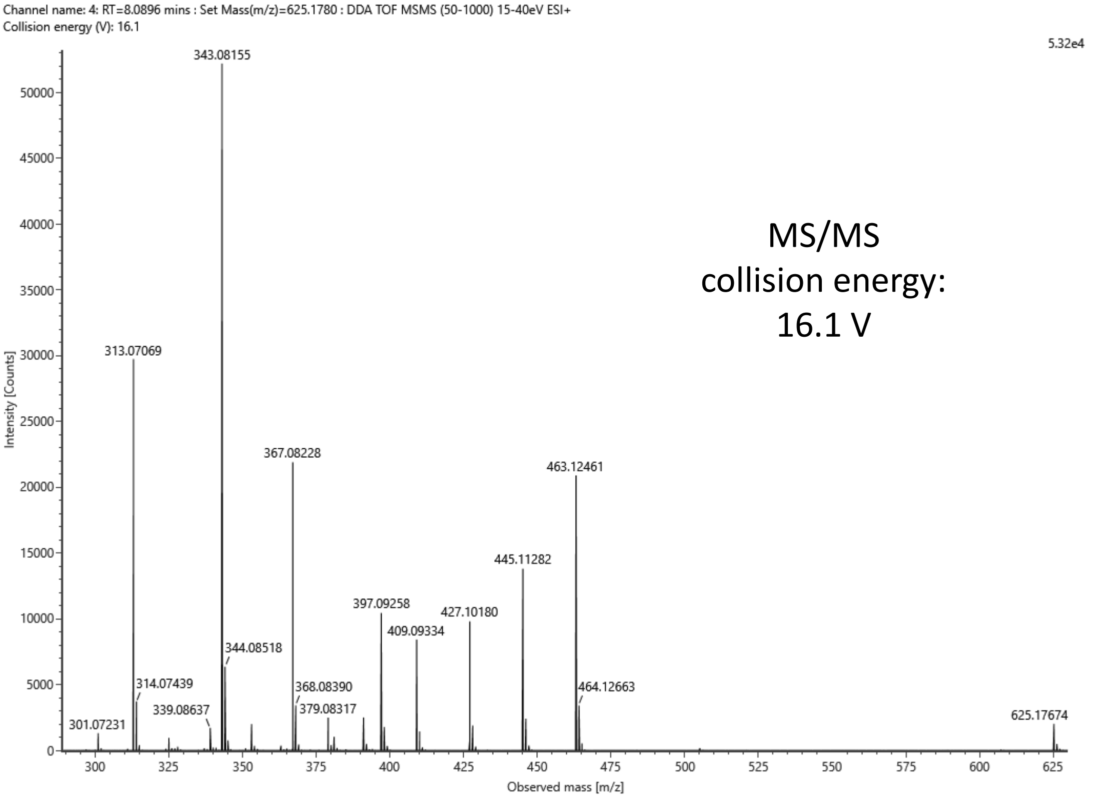

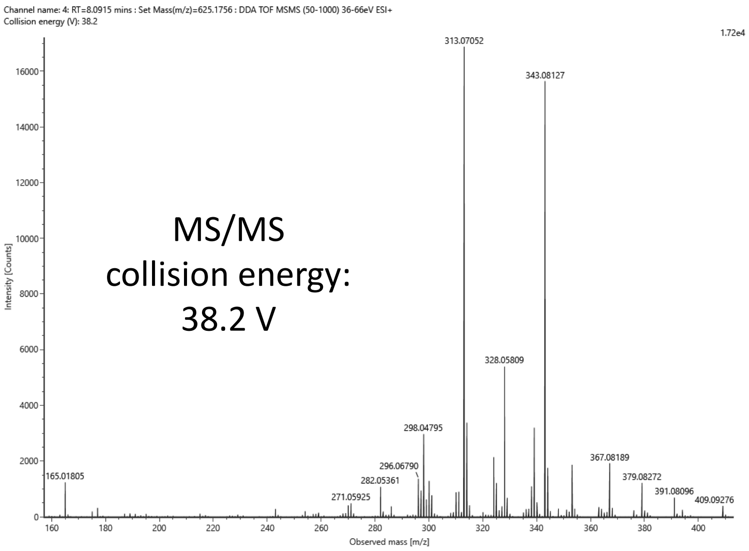

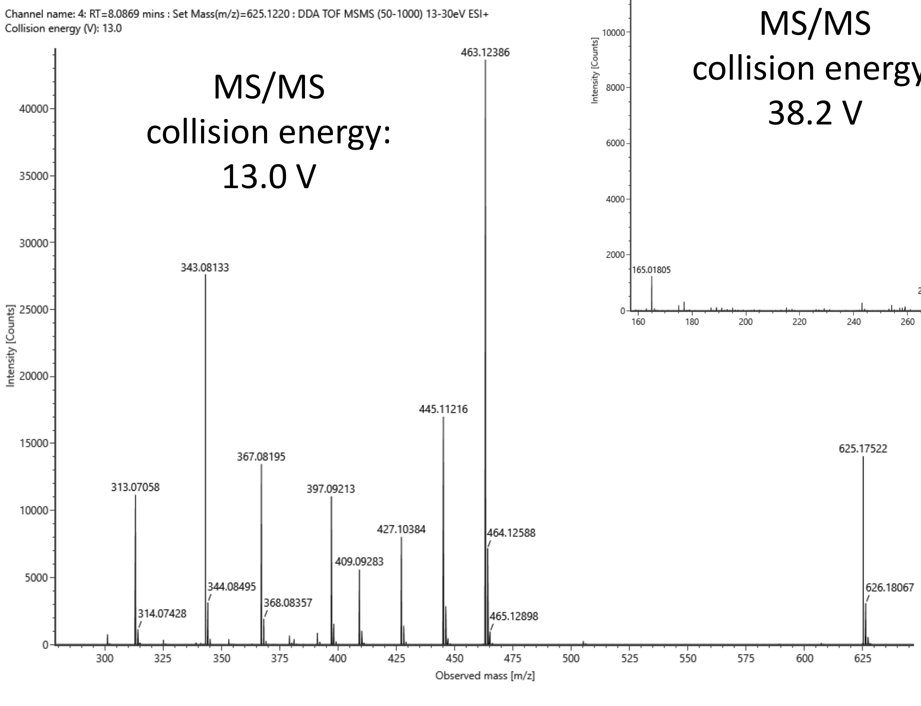

Compound name: isoscoparin-2"-O-glucoside
ESI-MS mode: negative, Unispray ion source
m/z (experimental): 623.16157

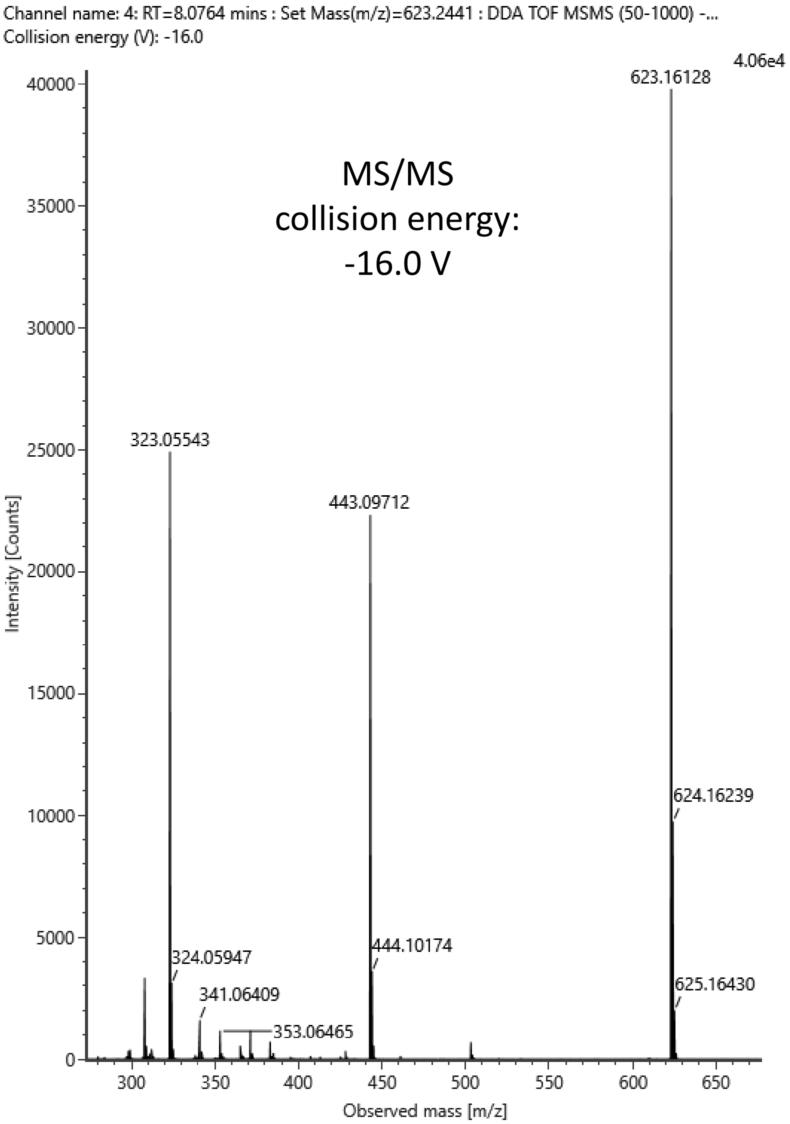

Compound name: isovitexin-2-O-rhamnoside
ESI-MS mode: positive, Unispray ion source
m/z (experimental): 579.17094

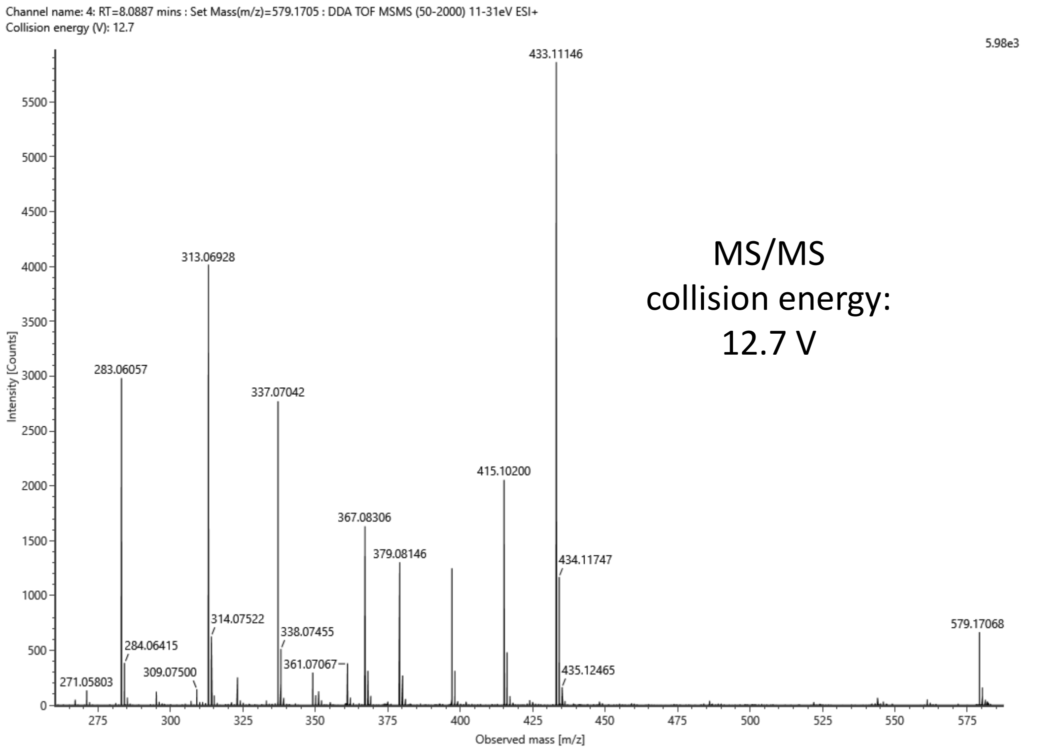

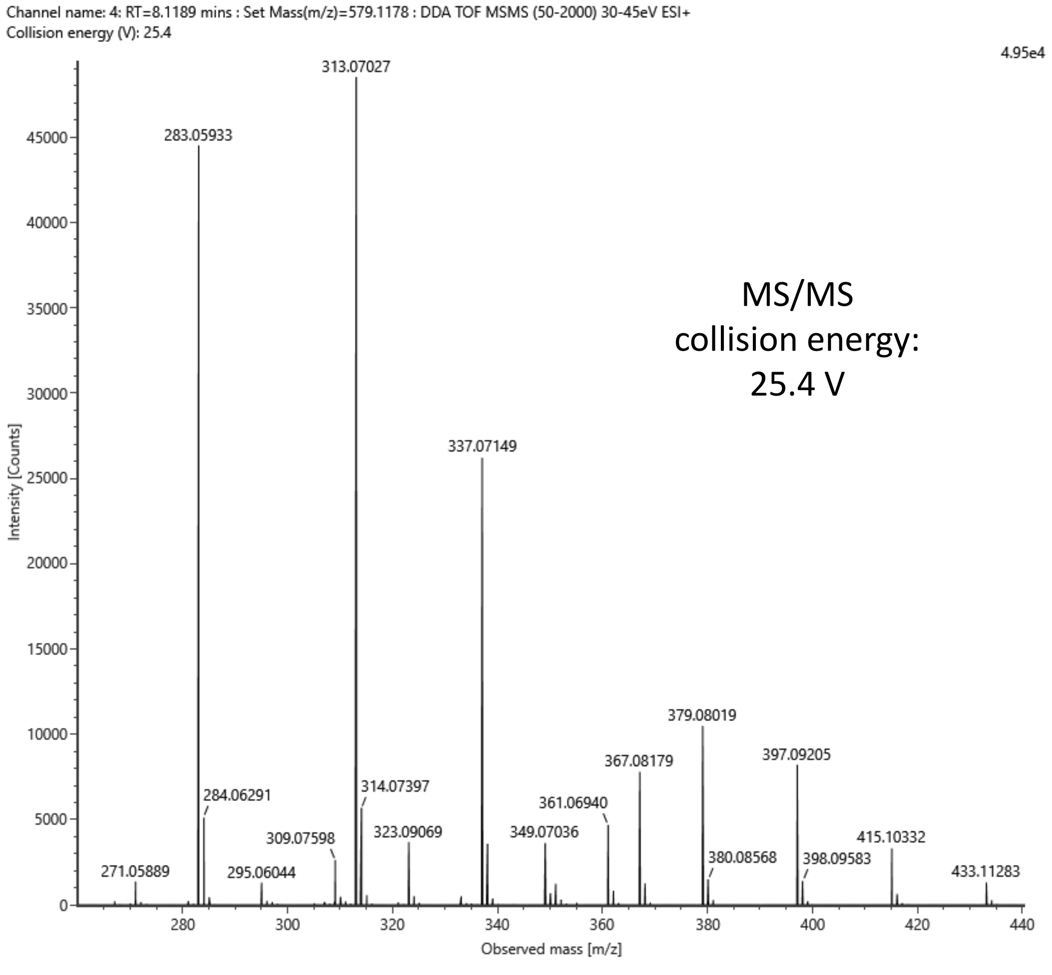

Compound name: isovitexin-2-O-rhamnoside
ESI-MS mode: negative, Unispray ion source
m/z (experimental): 577.15639

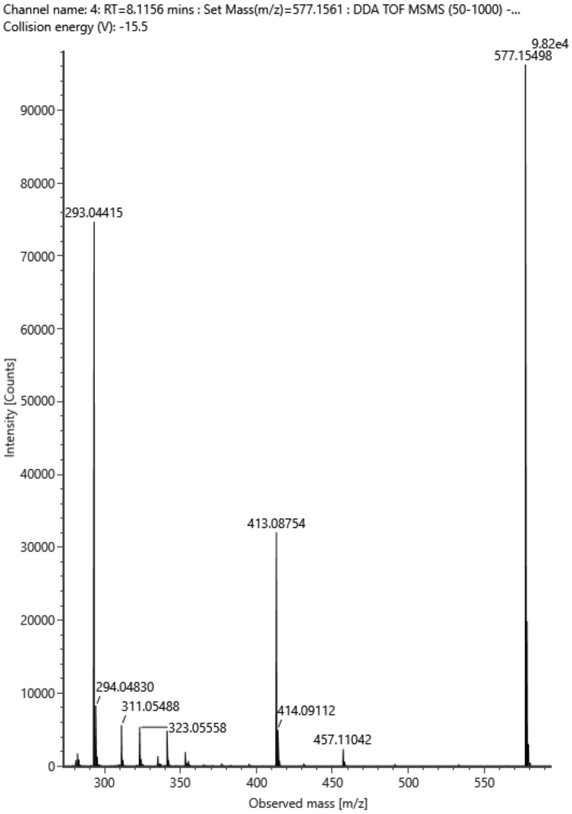

MS/MS
collision energy:
-15.5 V

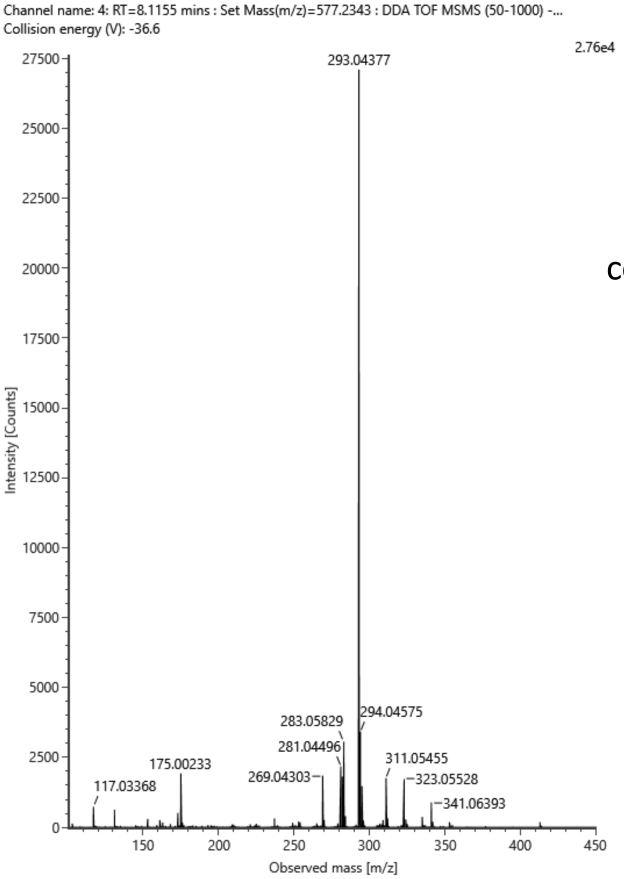

MS/MS
collision energy:
-36.6 V

Compound name: C-hexosyl-chrysoeriol O-rhamnoside
ESI-MS mode: positive, Unispray ion source
m/z (experimental): 609.18146

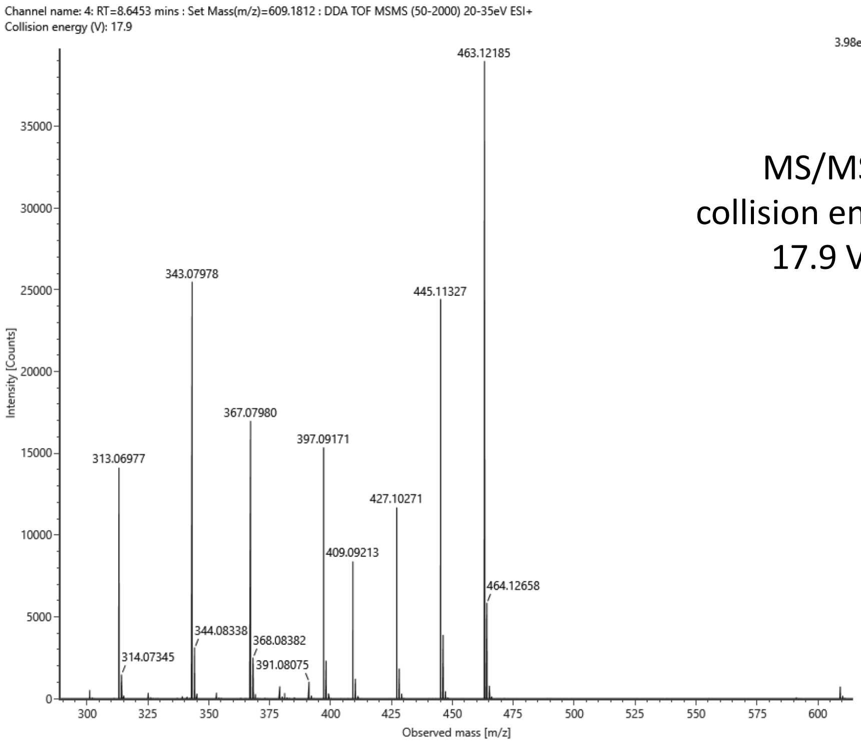

MS/MS
collision energy:
17.9 V

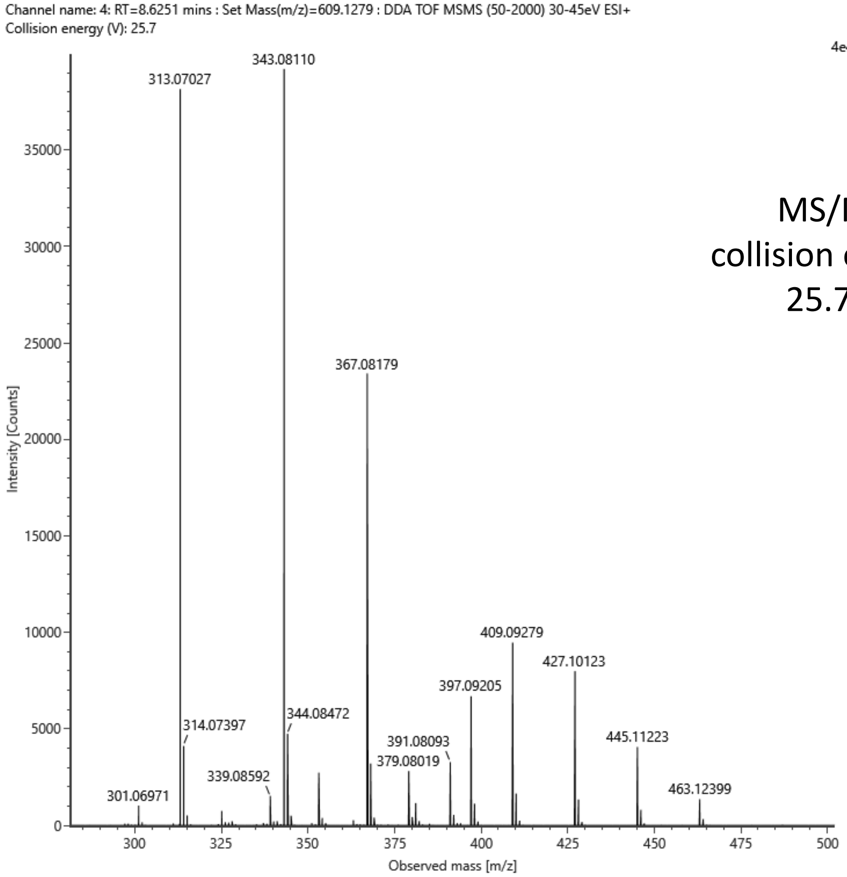

MS/MS
collision energy:
25.7 V

Compound name: C-hexosyl-chrysoeriol O-rhamnoside
ESI-MS mode: negative, Unispray ion source
m/z (experimental): 607.16634

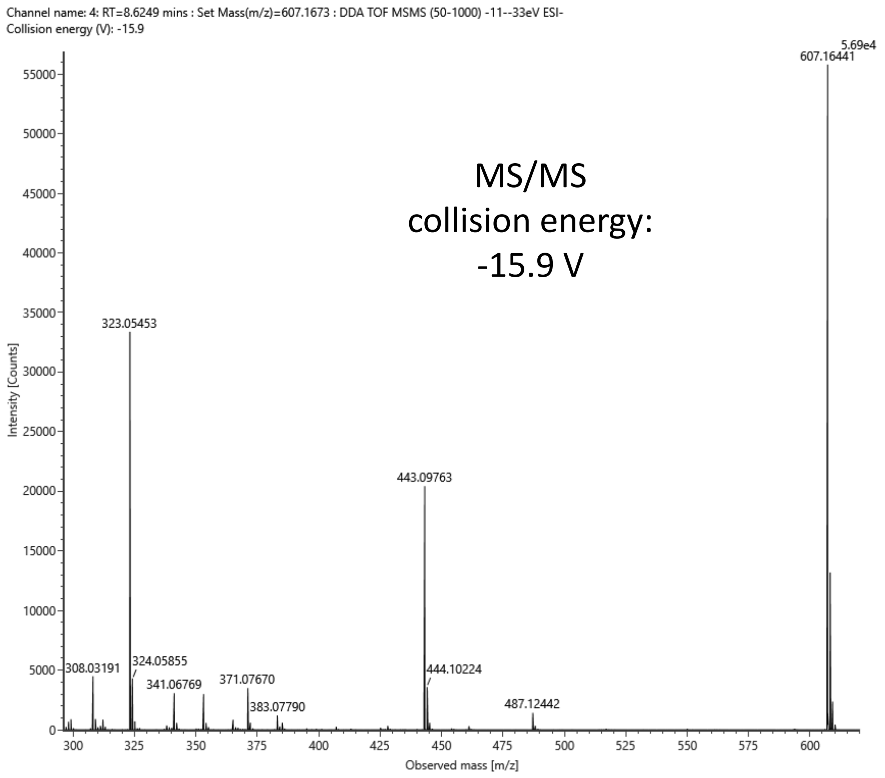

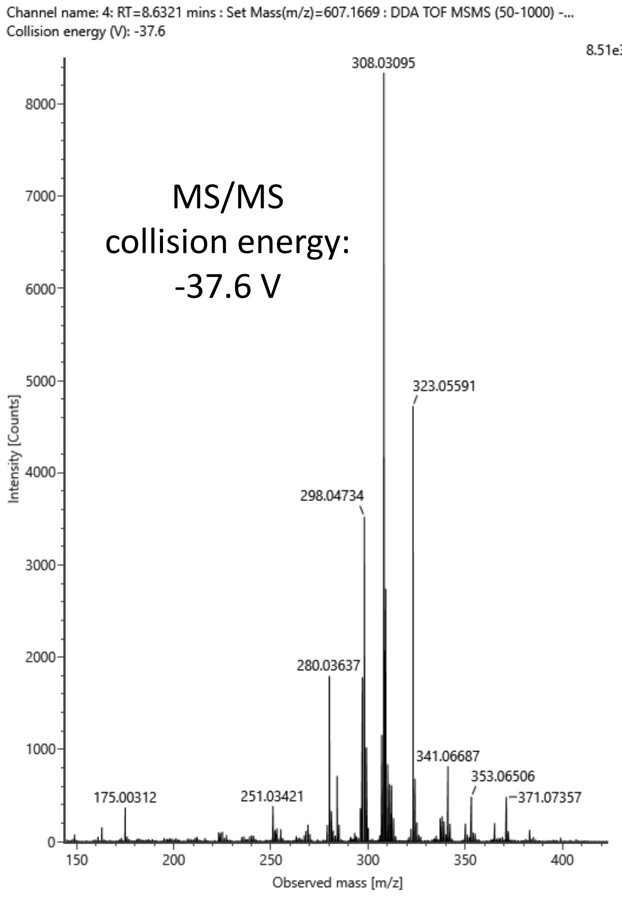

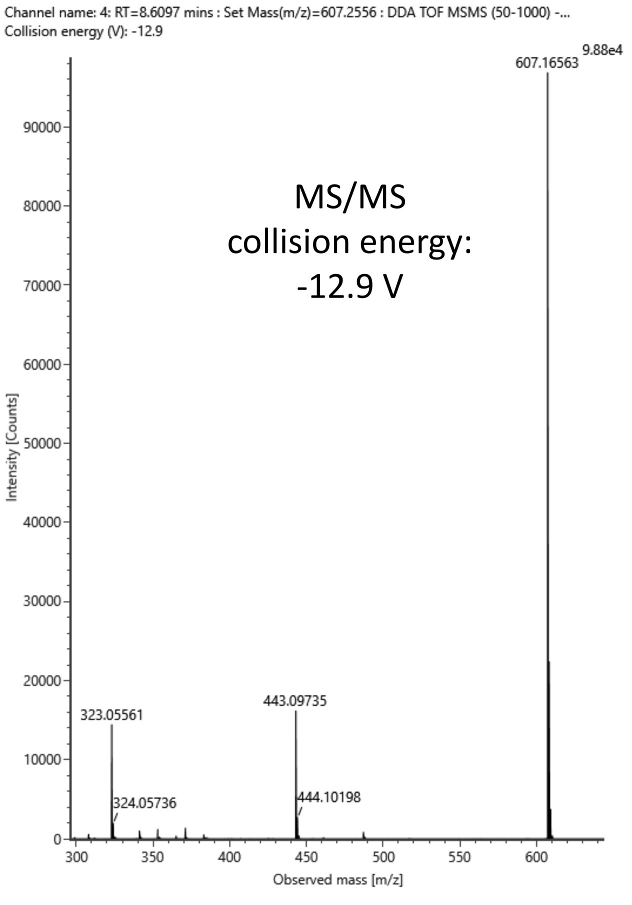

Compound name: C-hexosyl-luteolin O-feruloylpentoside
ESI-MS mode: positive, Unispray ion source
m/z (experimental): 757.19699

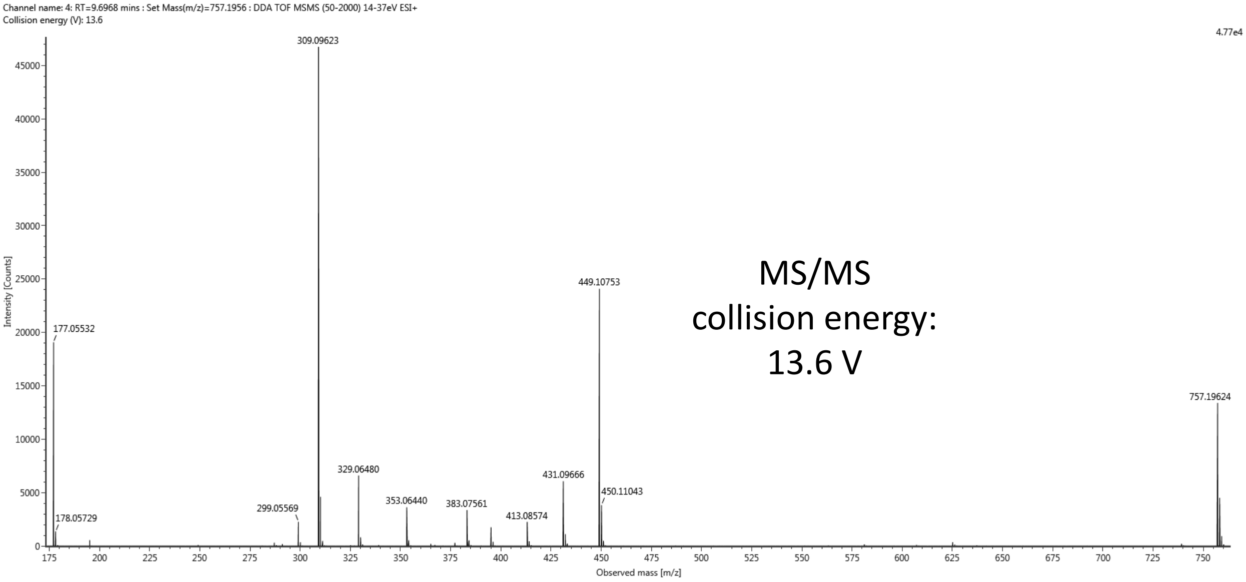

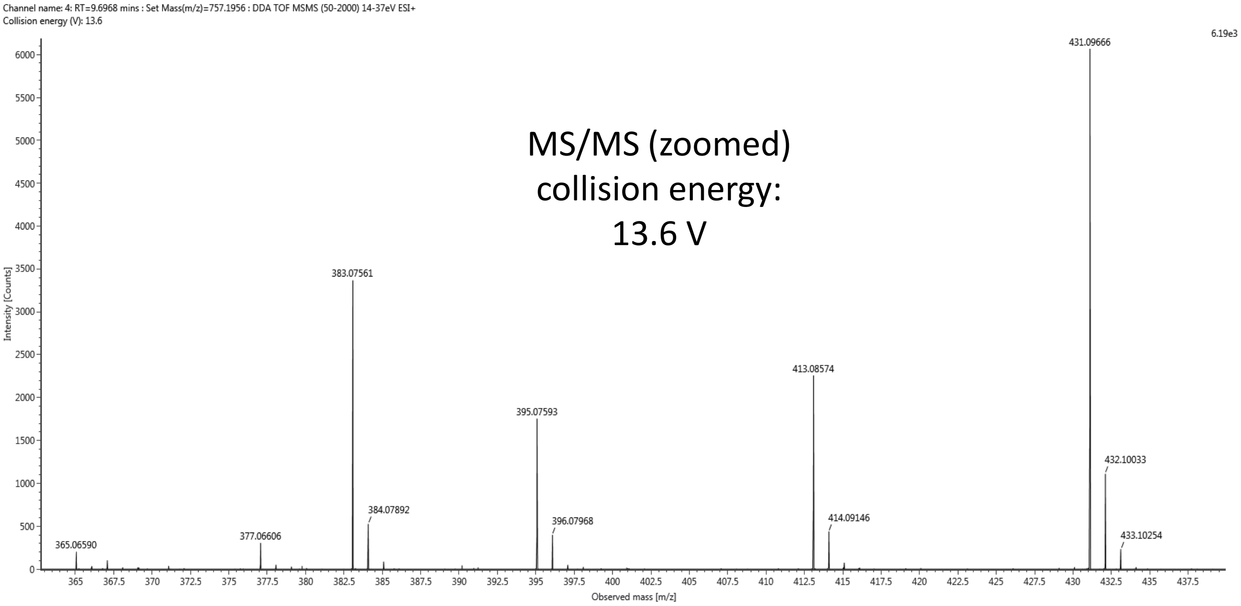

Compound name: C-hexosyl-luteolin O-feruloylpentoside
ESI-MS mode: negative, Unispray ion source
m/z (experimental): 755.18230

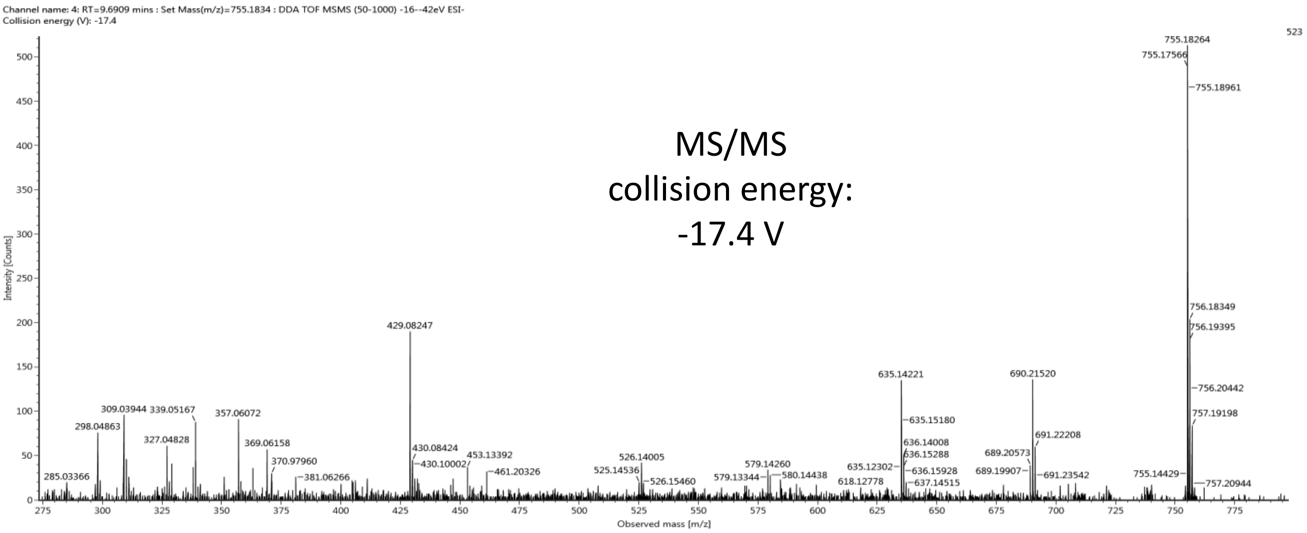

Compound name: isovitexin 2''-O-(6'''-feruloyl)glucoside
ESI-MS mode: positive, Unispray ion source
m/z (experimental): 771.21326

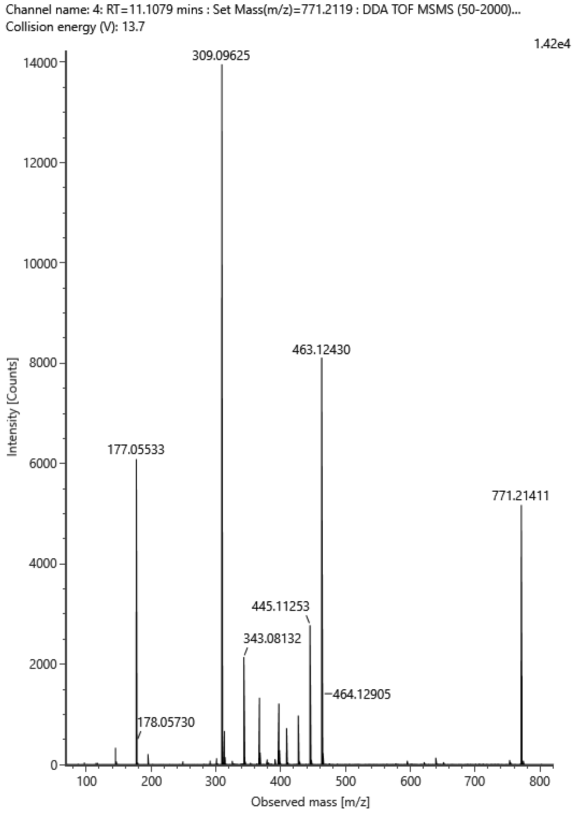

MS/MS
collision energy:
13.7 V

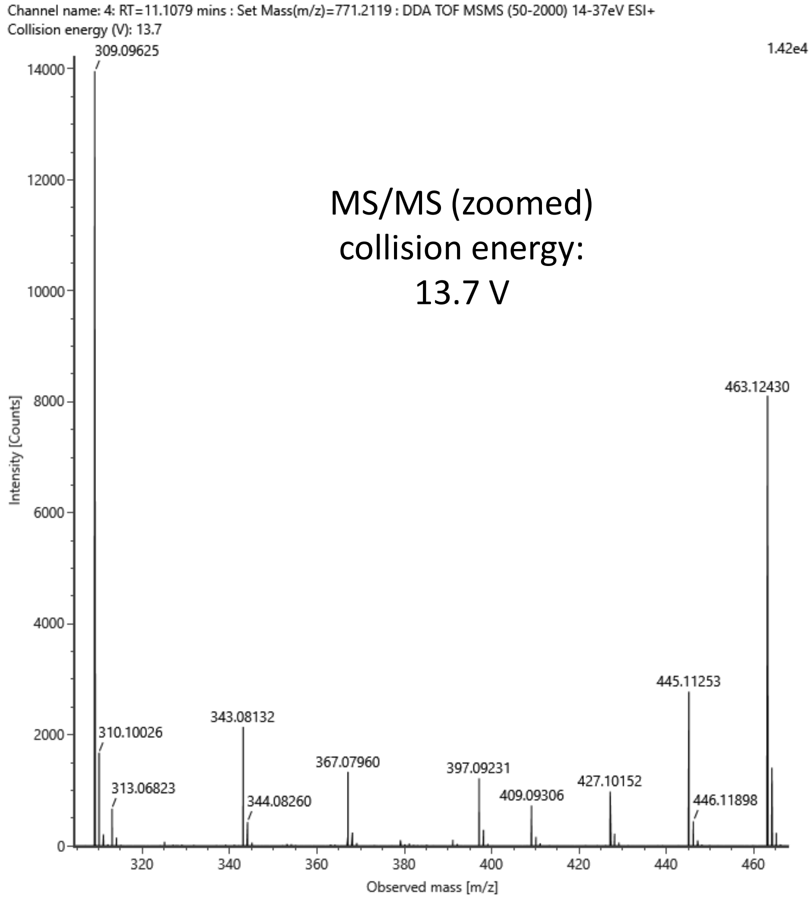

MS/MS (zoomed)
collision energy:
13.7 V

Compound name: isovitexin 2''-O-(6'''-feruloyl)glucoside
ESI-MS mode: negative, Unispray ion source
m/z (experimental): 769.19858

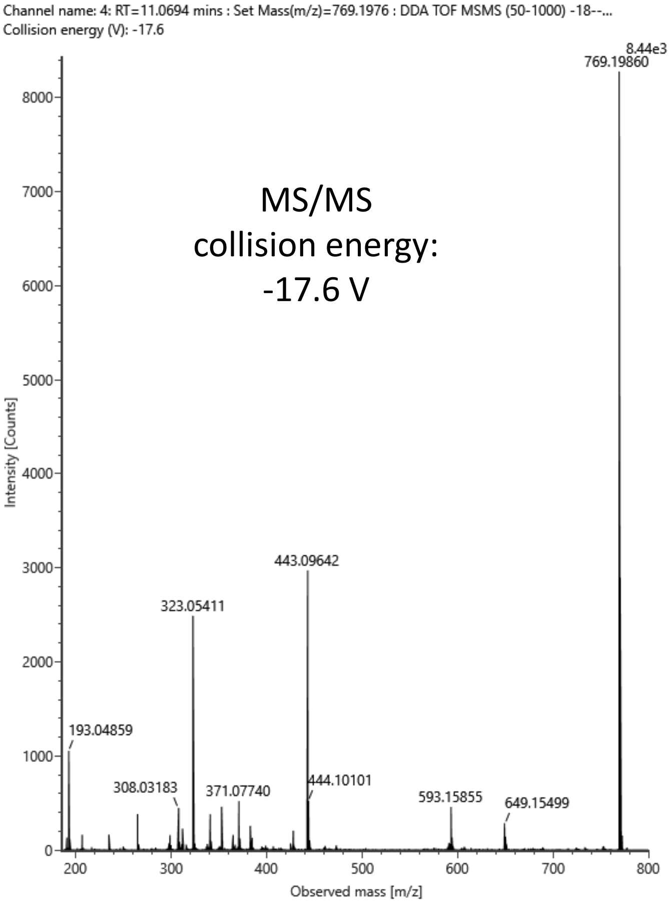

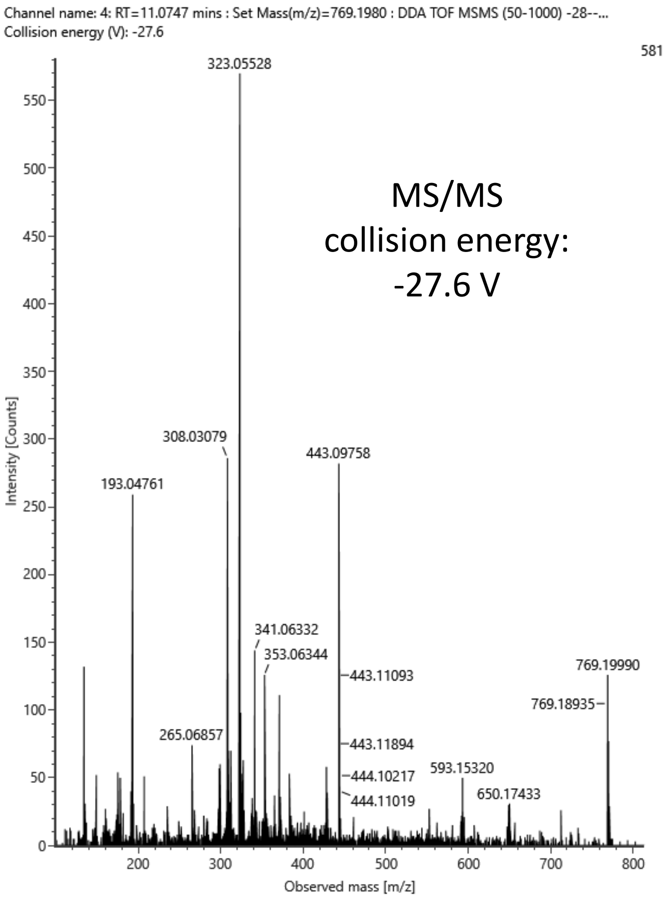

Compound name: 3'-O-methylluteolin (chrysoeriol)
ESI-MS mode: positive, Unispray ion source
m/z (experimental): 301.07079

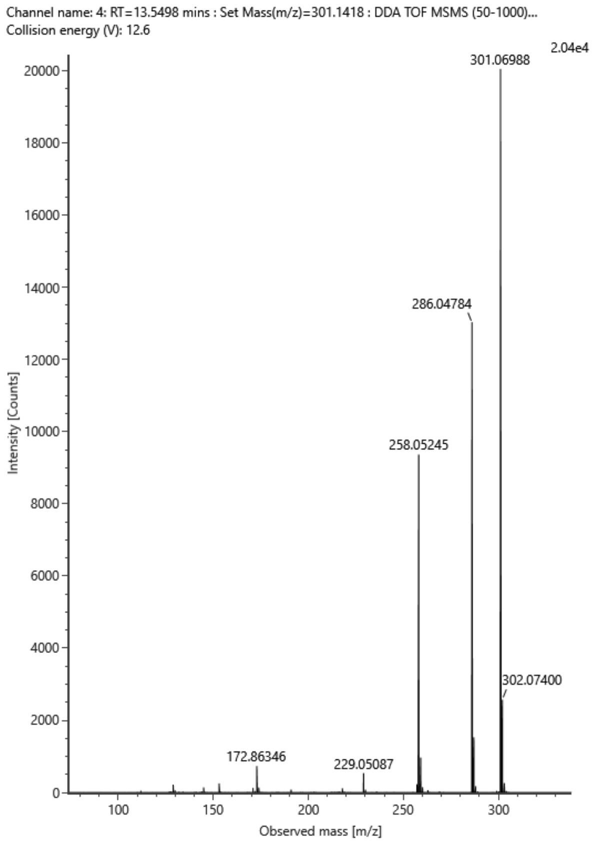

MS/MS
collision energy:
12.6 V

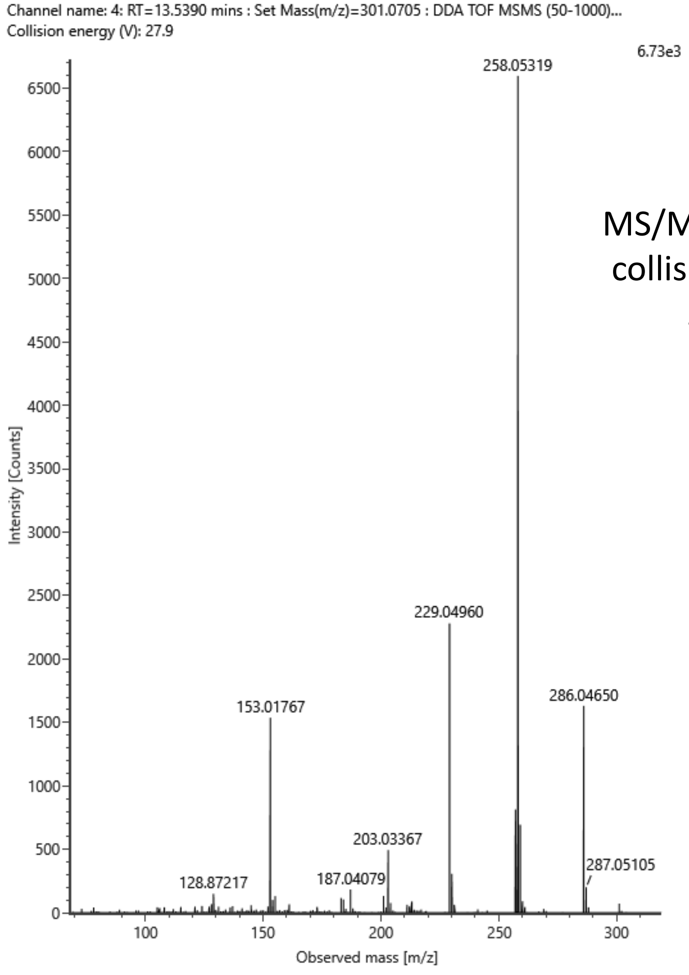

MS/MS (zoomed)
collision energy:
27.9 V

Compound name: 3'-O-methylluteolin (chrysoeriol)

ESI-MS mode: negative, Unispray ion source

m/z (experimental): 299.05627

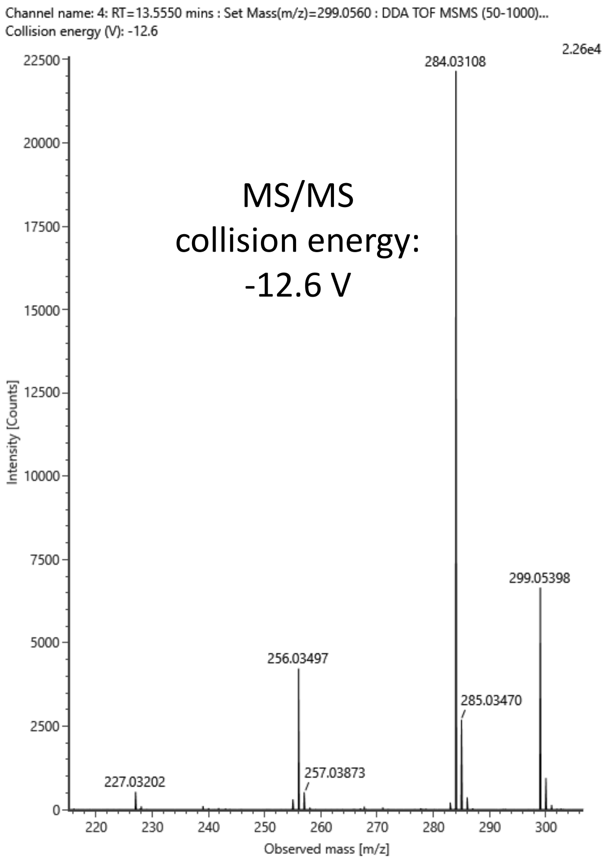

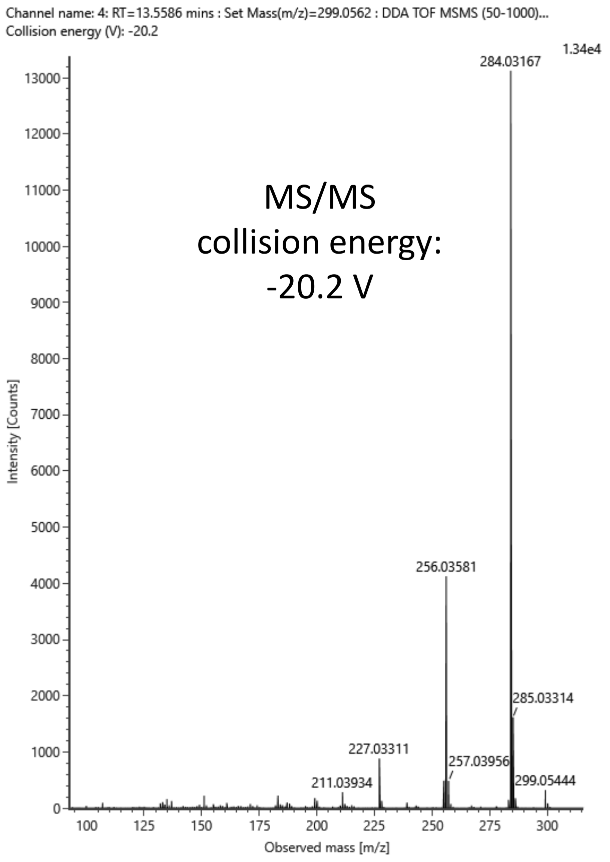

Supplement: Supplementary file 1 [file ijms-23-07969-s001.zip › ijms-1789205-supplementary/Figures S1-S7.pdf]
