# Supplementary material for: Mutations in Rht-B1 Locus May Negatively Affect Frost Tolerance in Bread Wheat
Source: Int J Mol Sci. 2022 Jul 19;23(14):7969. doi: 10.3390/ijms23147969 (PMC9324540; doi:10.3390/ijms23147969)
Supplement: Supplementary file 1 [file ijms-23-07969-s001.zip › ijms-1789205-supplementary/Tables S2-S4.pdf]

Table S2. Quantitative summary of differentiating entities among the four tested wheat genotypes.

Comparison (based on S-plot arrangement)	Positive mode experiments			Negative mode experiments		
	Sum of all differentiating entities	Sum of differentiating entities over the intensity level required for adequate signal-to-noise level MS/MS analysis	Entities assigned to the eight polyamine derivatives or the 17 flavonoids	Sum of all differentiating entities	Sum of differentiating entities over the intensity level required for adequate signal-to-noise level MS/MS analysis	Entities assigned to the 17 flavonoids
Rht-B1a vs. *Rht-B1b* Day 0	54	9	2	67	14	9
Rht-B1a vs. *Rht-B1b* Day 2	325	14	0	57	11	8
Rht-B1a vs. *Rht-B1b* Day 7	76	22	20	53	10	9
Rht-B1a vs. *Rht-B1b* Day 12	13	12	12	32	12	9
Rht-B1a vs. *Rht-B1c* Day 0	177	19	10	161	11	10
Rht-B1a vs. *Rht-B1c* Day 2	726	36	28	478	15	11
Rht-B1a vs. *Rht-B1c* Day 7	213	23	18	350	11	9
Rht-B1a vs. *Rht-B1c* Day 12	72	17	14	188	17	10
Rht-B1b vs. *Rht-B1c* Day 0	86	14	10	107	13	11
Rht-B1b vs. *Rht-B1c* Day 2	241	17	15	183	12	10
Rht-B1b vs. *Rht-B1c* Day 7	51	11	7	128	15	10
Rht-B1b vs. *Rht-B1c* Day 12	39	13	11	135	21	9
Rht-B1a vs. Béres Day 0	288	25	8	520	18	10
Rht-B1a vs. Béres Day 2	519	21	6	451	19	7
Rht-B1a vs. Béres Day 7	316	20	8	445	13	5
Rht-B1a vs. Béres Day 12	593	47	24	552	21	9
Rht-B1b vs. Béres Day 0	246	26	5	387	13	6
Rht-B1b vs. Béres Day 2	330	30	5	349	21	7
Rht-B1b vs. Béres Day 7	404	32	20	619	15	7
Rht-B1b vs. Béres Day 12	508	44	24	596	17	7
Rht-B1c vs. Béres Day 0	274	44	14	467	10	6
Rht-B1c vs. Béres Day 2	589	53	24	554	14	7
Rht-B1c vs. Béres Day 7	643	40	24	725	20	10
Rht-B1c vs. Béres Day 12	589	49	20	444	15	7

Table S3. Statistically differentiating oligohexoses (fructans) detected in the four wheat genotypes with hydrophilic interaction chromatography (HILIC). (*) refers to $[M+Na]^+$, ($^{\#}$) refers to $[M+2Na]^{2+}$.

Retention time, min	Tentative identification	Elemental composition (neutral)	Theoretical m/z	Experimental m/z	Difference, ppm
11.50	Oligo-5	C30H52O26	851.26390*	851.26530	1.64
12.16	Oligo-6	C36H62O31	1013.31673*	1013.31830	1.55
12.70	Oligo-7	C42H72O36	1175.36955*	1175.37179	1.91
13.18	Oligo-8	C48H82O41	1337.42237*	1337.42487	1.87
13.59	Oligo-9	C54H92O46	1499.47520*	1499.47753	1.55
13.98	Oligo-10	C60H102O51	1661.52802*	1661.53092	1.75
14.26	Oligo-11	C66H112O56	1823.58084*	1823.58437	1.94
14.61	Oligo-12	C72H122O61	1985.63367*	1985.63767	2.01
14.86	Oligo-13	C78H132O66	1085.33786$^{\#}$	1085.33942	1.44
15.13	Oligo-14	C84H142O71	1166.36427$^{\#}$	1166.36531	0.89
15.37	Oligo-15	C90H152O76	1247.39068$^{\#}$	1247.39281	1.71
15.64	Oligo-16	C96H162O81	1328.41709$^{\#}$	1328.41879	1.28
15.82	Oligo-17	C102H172O86	1409.44350$^{\#}$	1409.44581	1.64
16.00	Oligo-18	C108H182O91	1490.46991$^{\#}$	1490.47049	0.39

Table S4. UPLC/HPLC-PDA-Unispray-QTOF-MS instrumental setup parameters

**Acquity I-Class UPLC**		**Vion PDA IMS Unispray (+/-) -QTOF-MS**	
**UPLC column**	**Acquity BEH C18, 2.1*100 mm; 1.7 µm**	Source temperature	120°C
Eluent “A”	water with 0.1 v/v% formic acid	Desolvation temperature	550°C
Eluent “B”	acetonitrile with 0.1 v/v% formic acid	Desolvation gas	1000 L/h
Flow rate	0.5 ml/min	Cone gas	100 L/h
Column temperature	30°C	IMS<br>MS scan<br>MS scan time<br>Lock mass<br>MS/MS scan	OFF<br>100 – 2000 m/z<br>0.4 s<br>ON<br>50 – 2000 m/z
Gradient	0 – 0.5 min 4% “B” 0.5 – 10.0 min ↑20% “B” 10.0 – 15.0 min ↑40% “B” 15.0 – 20.0 min ↑80% “B” 20.0 – 20.5 min ↑100% “B” 20.5 – 23.0 min 100% “B” 23.0 – 23.5 min ↓4% “B” 23.5 – 26.0 min 4% “B”	MS^E Low collision energy	6.0 eV
Injection volume	3.0 µl	MS^E High collision energy ramp	20 – 30 eV
Sample temperature	8°C	Capillary voltage	0.30 kV
**HPLC column**	**SeQuant ZIC-cHILIC, 2.1*100 mm; 3.0 µm**	Cone voltage	40 V
Eluent “A”	water + 5 mM ammonium acetate	MS/MS collision energy	Individually set between 11.1-37.7 V and (-12.6) – (-37.7) V
Eluent “B”	(9:1 acetonitrile:water) + 5 mM ammonium acetate	**PDA detector**	
Flow rate	0.35 ml/min	Sampling rate	20 points/sec
Column temperature	30°C	Wavelength	220-600 nm
Gradient	0 – 0.5 min 100% “B” 0.5 – 20.0 min ↓40% “B” 20.0 – 22.0 min 40% “B” 22.0 – 23.0 min ↑100% “B” 20.0 – 20.5 min ↑100% “B” 23.0 – 30.0 min 100% “B”	Resolution	2.4 nm
Injection volume	2.0 µl		
Sample temperature	8°C		
